# Efficient and sustained *FOXP3* locus editing in hematopoietic stem cells as a therapeutic approach for IPEX syndrome

**DOI:** 10.1016/j.omtm.2023.101183

**Published:** 2023-12-26

**Authors:** Swati Singh, Cole M. Pugliano, Yuchi Honaker, Aidan Laird, M. Quinn DeGottardi, Ezra Lopez, Stefan Lachkar, Claire Stoffers, Karen Sommer, Iram F. Khan, David J. Rawlings

**Affiliations:** 1 Center for Immunity and Immunotherapies and the Program for Cell and Gene Therapy, Seattle Children’s Research Institute, Seattle, WA 98101, USA; 2 Department of Pediatrics, University of Washington, Seattle, WA 98101, USA; 3 Department of Immunology, University of Washington, Seattle, WA 98101, USA

**Keywords:** FOXP3, IPEX, hematopoietic stem cell, HSC editing, gene editing, CRISPR

## Abstract

Immune dysregulation, polyendocrinopathy, enteropathy, X-linked (IPEX) syndrome is a monogenic disorder caused by mutations in the *FOXP3* gene, required for generation of regulatory T (T_reg_) cells. Loss of T_reg_ cells leads to immune dysregulation characterized by multi-organ autoimmunity and early mortality. Hematopoietic stem cell (HSC) transplantation can be curative, but success is limited by autoimmune complications, donor availability and/or graft-vs.-host disease. Correction of FOXP3 in autologous HSC utilizing a homology-directed repair (HDR)-based platform may provide a safer alternative therapy. Here, we demonstrate efficient editing of *FOXP3* utilizing co-delivery of Cas9 ribonucleoprotein complexes and adeno-associated viral vectors to achieve HDR rates of >40% *in vitro* using mobilized CD34^+^ cells from multiple donors. Using this approach to deliver either a GFP or a FOXP3 cDNA donor cassette, we demonstrate sustained bone marrow engraftment of approximately 10% of HDR-edited cells in immune-deficient recipient mice at 16 weeks post-transplant. Further, we show targeted integration of FOXP3 cDNA in CD34^+^ cells from an IPEX patient and expression of the introduced FOXP3 transcript in gene-edited primary T cells from both healthy individuals and IPEX patients. Our combined findings suggest that refinement of this approach is likely to provide future clinical benefit in IPEX.

## Graphical abstract

**Figure:**
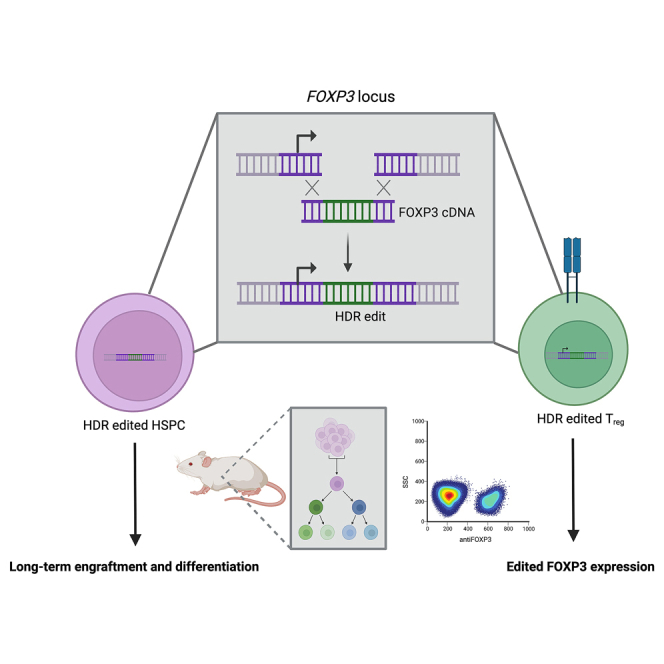

## Teaser

Singh and colleagues describe a CRISPR-Cas9-based gene editing strategy to treat IPEX syndrome, a monogenic immune disorder caused by mutations in the *FOXP3* gene. The authors demonstrate long-term *in vivo* engraftment of edited HSCs and efficient editing of IPEX patient cells.

## Introduction

Immune dysregulation, polyendocrinopathy, enteropathy, X-linked (IPEX) syndrome is a rare monogenic primary immunodeficiency, characterized by the loss of functional regulatory T (T_reg_) cells crucial for controlling immune responses against self and foreign antigens. The syndrome was first described in 1982^1^ in a family with several affected males and the responsible gene, Forkhead box P3 (FOXP3), was identified several years later.^2^^,^^3^ FOXP3 is the lineage-defining transcription factor of thymically derived T_reg_ cells and is essential for both T_reg_ cell development and function. Absent or dysfunctional T_reg_ cell in IPEX patients leads to the failure to maintain peripheral immune tolerance, resulting in the early onset of multi-system autoimmunity with features including, most commonly, severe inflammatory bowel disease, type 1 diabetes mellitus, thyroid disease, and eczema. Supportive immunosuppressive therapies can modulate disease, but are not curative and are associated with multiple complications. Alternatively, allogeneic hematopoietic stem cell transplantation (HSCT) represents a potentially curative approach that can eliminate autoimmune manifestations.^4^ However, while HSCT can be highly beneficial for IPEX, limitations in donor matching and transplant complications in the setting of severe immunologic dysregulation make implementation of this approach highly challenging.^4^^,^^5^

Prior work has demonstrated the efficacy of lentiviruses (LVs) expressing FOXP3 from a constitutively active EF1α promoter to establish a T_reg_ cell phenotype in IPEX CD4^+^ T cells.^6^ While potentially advantageous for applications where adoptive transfer of T_reg_ cells might be beneficial,^7^^,^^8^ adoptively transferred LV-treated T cells are unlikely to provide a long-term cure for IPEX due to the inability to persist *in vivo* over long periods of time. Gene therapy of murine HSCs using LV with the endogenous FOXP3 promoter driving FOXP3 cDNA expression have also shown promise by rescuing the autoimmune phenotype in scurfy mice, the murine equivalent of IPEX.^9^ This approach, however, will require LVs that can precisely replicate the complex endogenous control elements within the *FOXP3* locus capable of initiating and sustaining endogenous levels of FOXP3 expression, while limiting both vector silencing and genotoxicity risk due to the random nature of LV integration.

CRISPR-Cas9-based gene editing^10^^,^^11^ of IPEX patient CD34^+^ hematopoietic stem and progenitor cells (HSPCs) offers an alternative therapeutic option. Homology-directed repair (HDR)-based editing enables insertion of functional FOXP3 cDNA sequence into the endogenous locus while preserving adjacent sequence elements required for expression and ensuring appropriate copy number. Gene editing for IPEX is particularly appealing since locus- and lineage-specific regulation of *FOXP3* is critical for stable FOXP3 expression.^12^^,^^13^ Thus, preserving the natural genomic landscape at the locus in HDR-edited HSPCs is paramount to enable appropriate differentiation and acquisition of T_reg_ cell fate.

While *in vitro* editing has been reported at high rates in HSPCs sourced from umbilical cord blood (CB) or isolated from peripheral blood of granulocyte colony stimulating factor (G-CSF) mobilized adults (mPB), engraftment of HDR-edited HSPCs in immune deficient animals remains challenging due to the difficulty in targeting long-term repopulating HSCs (LT-HSCs); a feature that is particularly evident when using mPB HSPCs.^14^ Importantly, analysis of in IPEX patients following HSCT and female carriers of IPEX suggests that even low levels of functional FOXP3 expression may be sufficient to alleviate disease symptoms due to the high selective advantage for T_reg_ cells.^15^^,^^16^^,^^17^^,^^18^ Thus, engraftment of even a limited proportion of successfully HDR-edited HSC is predicted to provide clinical benefit in the setting of this profound immune disorder.

We have recently demonstrated highly efficient methods to edit the *FOXP3* locus in primary T cells enabling conversion to thymus T_reg_ (tT_reg_)-like cells capable of mediating immunosuppression in the setting of autoimmunity.^19^ Another group has published HDR-based editing of *FOXP3*, although the majority of experiments utilize CB-HSPCs rather than more clinically relevant mPB CD34^+^ HSPCs.^20^ Here, we present a targeted gene editing strategy to incorporate the codon diverged coding region for *FOXP3* gene in mPB HSPCs and test their ability to engraft in immune deficient mice. Further, we demonstrate efficient HDR-editing of IPEX patient CD34^+^ cells and verify the expression of codon-diverged FOXP3 transcripts in HDR-edited primary CD4^+^ T cells derived from both healthy controls and IPEX patients.

## Results

### Optimization of culture conditions for editing CD34^+^ cells at the *FOXP3* locus

**Figure 1:**
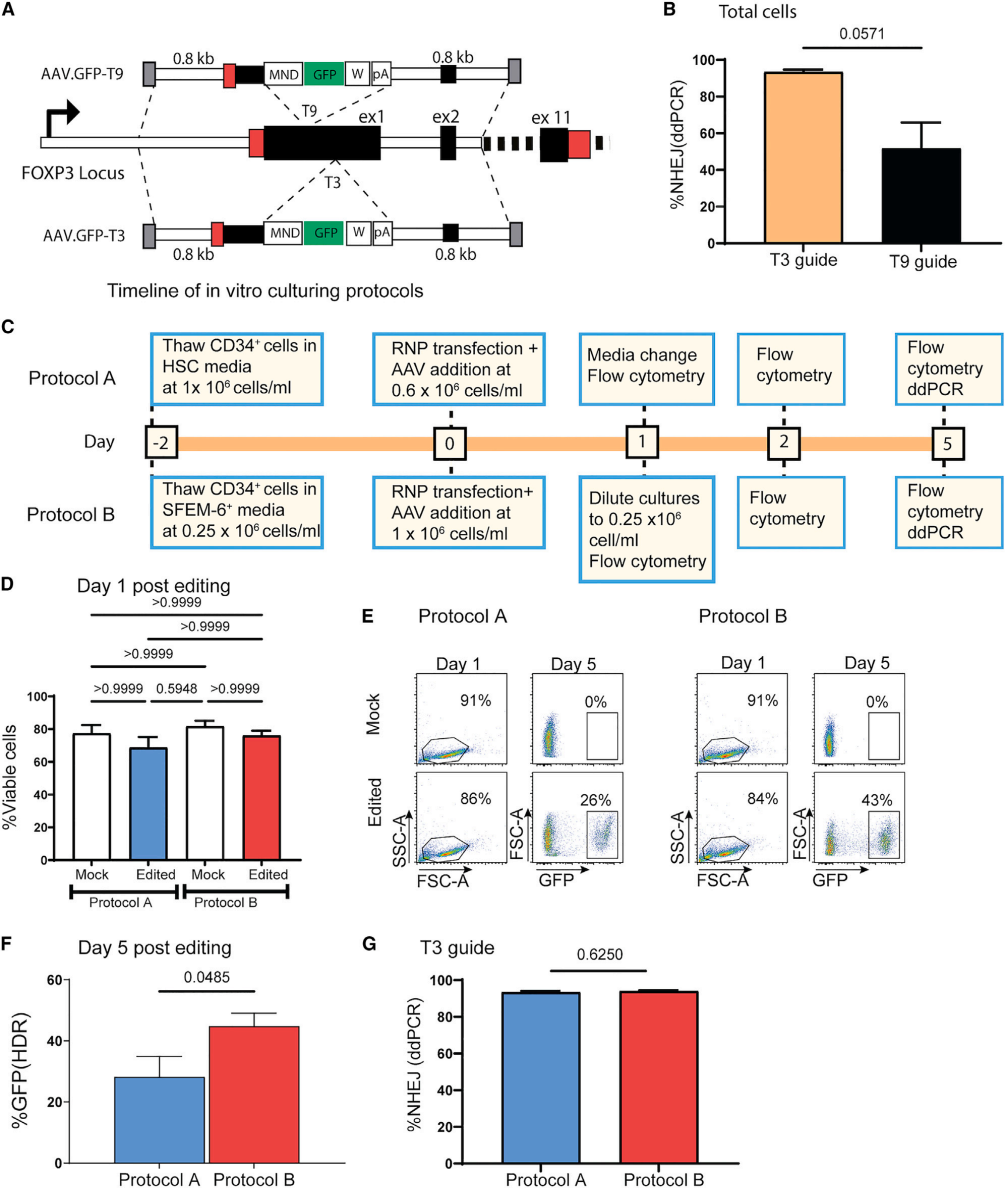
Targeted gene integration in primary human CD34^+^ HSPCs treated with CRISPR-Cas RNPs and AAV6 vectors (A) Schematic of the *FOXP3* genomic locus and the AAV6 targeting vectors specific for sgRNAs T9 or T3 designed to insert the MND promoter-driven GFP expression cassette into exon 1 of *FOXP3*. Black and red rectangles represent exons and UTR elements, respectively; the location of homology arms with respect to the *FOXP3* locus is depicted by dashed lines. pA, SV40 polyadenylation sequence; W, WPRE3 element. (B) Average allelic disruption (% NHEJ) rates observed upon transfection of adult mobilized human CD34^+^ HSPCs with T3 (n = 3 male donors, 3 independent studies) or T9 (n = 3 male donors, 3 independent studies) RNPs quantified via ddPCR. Significance determined by Mann-Whitney *U* test. (C) Timeline of procedures for *in vitro* culturing and editing of adult mobilized CD34^+^ HSPCs using protocol A (top) and B (bottom). (D) Viability measured by flow cytometry forward and side scatter 1 day after editing with T3 RNP and rAAV6 targeting vector employing either protocol A (n = 6 male donors, 12 independent studies) or protocol B (n = 5 male donors, 1 female donor, 9 independent studies). Significance determined by Kruskal-Wallis test. (E) Representative flow cytometry plots depicting cell viabilities on day 1 and %GFP^+^ cells on day 5 in HSPCs edited with protocol A (left) or B (right). (F) Targeted integration rates measured by GFP-high 5 days after editing with T3 RNP and rAAV6 targeting vector cultured with protocol A (n = 4 male donors, 4 independent studies) or protocol B (n = 5 male donors, 1 female donor, 8 independent studies). Significance determined by Mann-Whitney *U* test. (G) NHEJ 5 days after editing in cells cultured with protocol A or B and transfected with T3 RNP (n = 3 male donors in 4 independent studies). Wilcoxon matched-pairs signed ranked test. Bar graphs represent mean ± SEM.

The *FOXP3* gene comprises 11 coding exons and mutations reported in IPEX patients have been identified throughout the entire gene.^21^ To develop an editing strategy that can work as a universal cure for IPEX patients, we elected to insert a functional FOXP3 cDNA within the first coding exon, to enable correction of all IPEX patients except those bearing mutations within the promoter region.^22^^,^^23^ This strategy was also utilized to disrupt the mutant FOXP3 allele and eliminate the potential for expression of a dominant negative mutant protein. We used CRISPR single-guide RNA (sgRNA) T9,^19^ and a second sgRNA T3, to target the first coding exon of the *FOXP3* gene (Figure 1A). For comparison, the sgRNA utilized by Goodwin et al.^20^ binds at the intersection of 5′ UTR/first coding exon and 47 and 125 nucleotides away from sgRNA T9 and T3, respectively. The sgRNAs were complexed with SpyFi Cas9^24^ to form ribonucleic proteins (hereafter referred to as RNPs) then electroporated into HSPCs using the Neon electroporation system. Analysis of gDNA by droplet digital PCR (ddPCR) revealed that T3 RNP induced insertions/deletions (indels) in approximately 93%, while T9 targeted approximately 52% of *FOXP3* alleles, respectively (Figure 1B), indicating superior on-target cleavage with T3 RNP in CD34^+^ cells.

To orchestrate and efficiently track HDR, recombinant adeno-associated virus (rAAV) donor templates were first designed to insert an expression cassette containing a constitutive myeloproliferative sarcoma virus enhancer, negative control region deleted, dl587rev primer-binding site substituted (MND) promoter driven GFP cDNA sequence followed by shortened woodchuck hepatitis post-transcriptional regulatory element (WPRE3) and Simian virus 40 polyadenylation (SV40 polyA) sequences. Flanking the expression cassette, 0.8-kb homology arms centered specifically on the respective sgRNA were designed such that a minimum deletion was created after targeted integration (Figure 1A). Editing with either sgRNA and sgRNA-specific AAV HDR donor combination led to robust HDR rates. As anticipated, we observed 4-fold higher targeted modification using T3- (GFP-T3) or T9-specific (GFP-T9) AAV donors (Figures S1A and S1C) when compared with a common AAV.GFP donor that functioned with either sgRNA, findings that also demonstrated no significant negative impacts on cell viability (Figures S1B and S1D). For the GFP-T3 vector, no enhancement in HDR was observed as vector dose was increased (multiplicity of infections [MOI] from 250 to 1,000), while a dose-dependent increase was observed with the GFP-T9 AAV donor (Figures S1A and S1C). Since absolute HDR rates were considerably higher at 51% for the T3 RNP and GFP-T3 AAV (vs. 8% using T9 RNP), we elected to utilize the T3 RNP for all subsequent experiments.

HSCs are largely quiescent, thereby limiting DNA damage due to replication and mitosis. Because HDR pathways are active primarily during G2 and S phases, HSCs are more likely to undergo non-homologous end-joining (NHEJ) upon introduction of a double-strand break (DSB).^25^^,^^26^ To facilitate entry of HSC into cell cycle and repair of DSBs by HDR utilizing the AAV donor, we first pre-stimulated CD34^+^ cells in culture media with cytokines. Of note, low-density cultures have also been reported to promote HSC expansion and cycling.^27^ Additionally, SR1 and UM171, compounds reported to support HSC expansion^28^ and self-renewal,^29^ were included in protocol B. To explore the impact of alternative culture densities and media on the indel and HDR frequencies, CD34^+^ cells were plated in media using a higher cell density (protocol A) vs. lower density protocol (protocol B), as outlined in Figure 1C. Specific differences between the two protocols are summarized in Table S1. For protocol B, the nucleofection was performed using the optimized program CM-149 (Figure S2). Flow cytometry analysis 1 day after editing revealed slightly higher cell viabilities in both mock and edited cells for protocol B (mock 79%, edited 72%) vs. protocol A (mock 72%, edited 63%) (Figure 1D). To assess potential differences in inducing indel and HDR frequencies, T3 RNPs and GFP-T3 AAV donor vectors were introduced into cells cultured using both protocols followed by flow cytometry to assess HDR rates based on % GFP^+^ cells at day 5 after editing. HDR rates were also subsequently assessed via ddPCR. A significantly higher average GFP expression was observed in the CD34^+^ cells cultured with protocol B averaging 44% compared with 28% for protocol A (Figures 1E and 1F), despite equivalent percent NHEJ rates (93%) using either protocol (Figure 1G).

### Edited HSPCs engraft long-term in NBSGW mice and undergo multi-lineage differentiation

**Figure 2:**
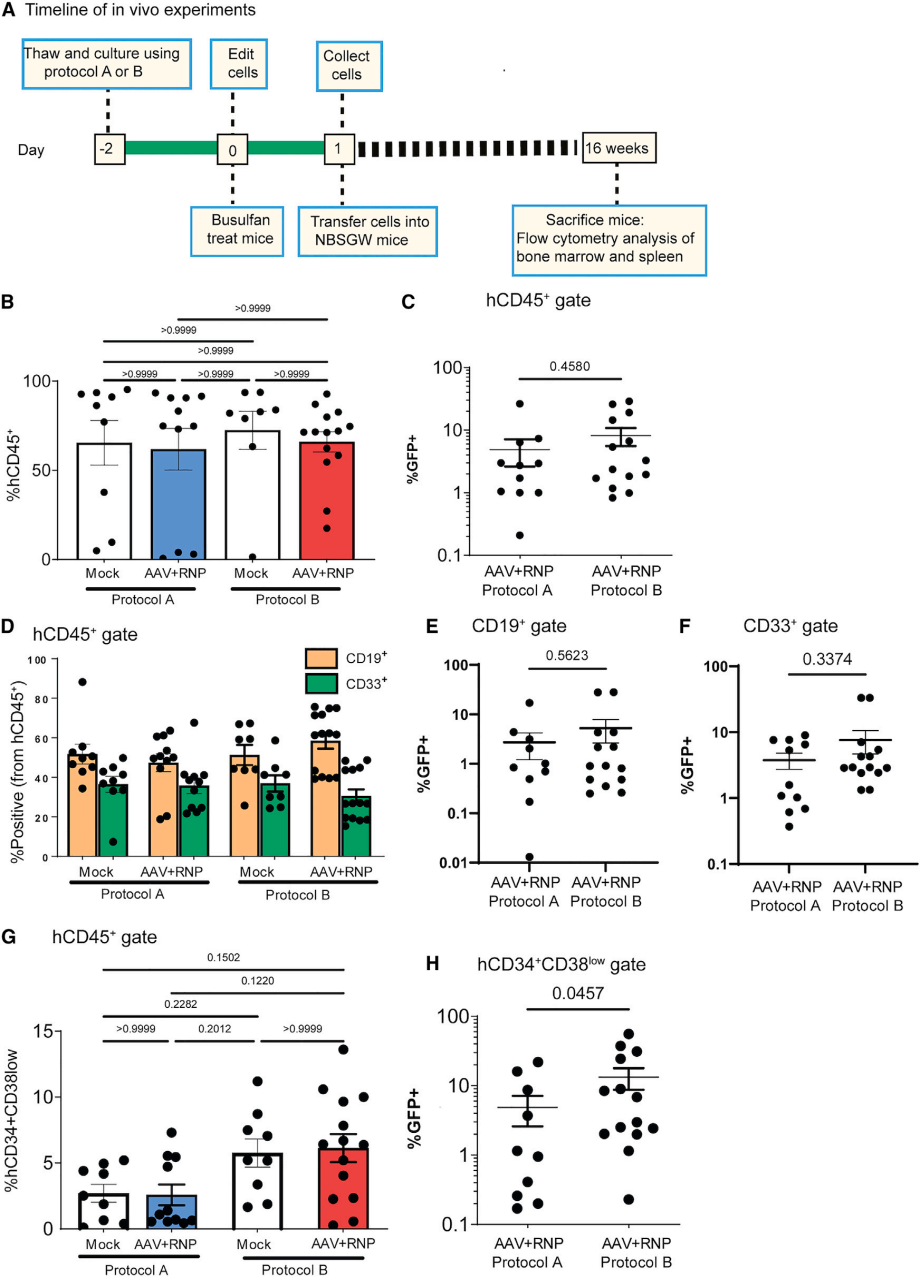
Long-term hematopoietic reconstitution from HDR-edited HSPCs in the BM of transplanted NBSGW mice (A) Procedure for *in vivo* engraftment of edited HSPCs in NBSGW mice. (B) Engraftment (%hCD45^+^) in the BM of NBSGW recipient mice 12–16 weeks after transplant of protocol A mock treated (n = 9, 2 male donors), protocol A edited (n = 11, 2 male donors), protocol B mock treated (n = 8, 1 male donor, 1 female donor), protocol B edited (n = 14, 1 male donor, 1 female donor) from two independent studies. Significance determined by Kruskal-Wallis test. (C) Percentage of hCD45^+^ HDR-edited (GFP+) cells in the BM of NBSGW recipients from (B) Significance determined by Mann-Whitney *U* test. (D) Proportion of B cells (CD19^+^) and myeloid cells (CD33^+^) in the BM of NBSGW recipients from (B). (E) Percentage of HDR-edited (GFP^+^) CD19^+^ cells from (D) Significance determined by Mann-Whitney *U* test. (F) Percentage of HDR-edited (GFP^+^) CD33^+^ cells from (D) Significance determined by Mann-Whitney *U* test. (G) Proportion of HSPCs (CD34^+^CD38low) in the BM of NBSGW recipients from (B) Significance determined by Kruskal-Wallis test. (H) Proportion of HDR-edited (GFP^+^) HSPCs (CD34^+^CD38low) from (G). Significance determined by Mann-Whitney *U* test. Bar graphs represent mean ± SEM.

To determine whether editing protocols influenced the long-term repopulation potential of CD34^+^ cells, mock or GFP-T3 AAV plus T3 RNP-edited CD34^+^ cells were transplanted into busulfan-treated, 8- to 10-week-old NBSGW recipient mice (Figure 2A). Human cell chimerism was evaluated by sacrificing the mice at 12–16 weeks after transplant and analyzing bone marrow (BM) and spleens for engrafted CD45^+^ human cells and GFP^+^ edited cells. Similar percentages of human cells were present within the BM for recipients of cells cultured with either protocol (protocol A: mock, 65%; edited 62%; and protocol B: mock 72%; edited 58%) (Figures 2B and S3A). However, the average %GFP^+^ cells observed within the BM were 1.6-fold higher when protocol B was used (average 8%; range, 0.2%–26.0%) compared with protocol A (average 5%; range, 1%–29%) (Figure 2C). The distribution of CD19^+^ B cells and CD33^+^ myeloid cells were similar across protocols and groups (Figure 2D). The %GFP^+^ cells within both compartments was slightly higher with protocol B compared with protocol A and CD33^+^ cells exhibited the highest %GFP^+^ cells (Figures 2E and 2F).

The mean human CD45^+^ cell engraftment within the spleen was also comparable between groups, ranging from 12% to 19%, with no significant differences between protocols (Figure S4A). Matching the trend observed in the BM, GFP^+^ cells were higher for protocol B compared with protocol A edited cells, averaging 10% and 4%, respectively (Figure S4B). The proportion of CD33^+^ cells were comparable across all groups. Interestingly, proportion of CD19^+^ B cells in both mock and edited groups was higher using protocol B (Figure S4C) and significantly higher %GFP^+^ cells were observed for protocol B, 5% compared with 2% for protocol A (Figure S4D).

A key obstacle for developing durable HDR-based gene editing therapies using HSPCs is the inherent quiescence of LT-HSCs that serves as a protective mechanism against endogenous stress.^30^ To assess the impact of alternative culture conditions on HDR editing in HSCs, we analyzed human cells within the BM of NBSGW mice for expression of surface markers that define a more primitive, engraftment-enriched subset of HSPCs, defined by CD34^+^CD38^low^ (Figure S3B). The proportion CD34^+^CD38^low^ HSPCs was approximately 2-fold higher with use of protocol B (5%–6%), irrespective of editing (Figure 2G). Strikingly, analysis of GFP^+^ cells within CD34^+^CD38^low^ compartment revealed a 3-fold higher proportion of HDR-edited cells for protocol B, 13% compared with 5% for protocol A (Figure 2H). These findings demonstrated that use of UM171 and SR1, in association with the low-density culturing in protocol B, enabled superior long-term engraftment of HSPCs and increased HDR editing efficiency in the CD34^+^CD38^low^ compartment and was selected for use in all ensuing studies.

### Sustained engraftment and differentiation of FOXP3 cDNA-edited HPSC in NBSGW mice

**Figure 3:**
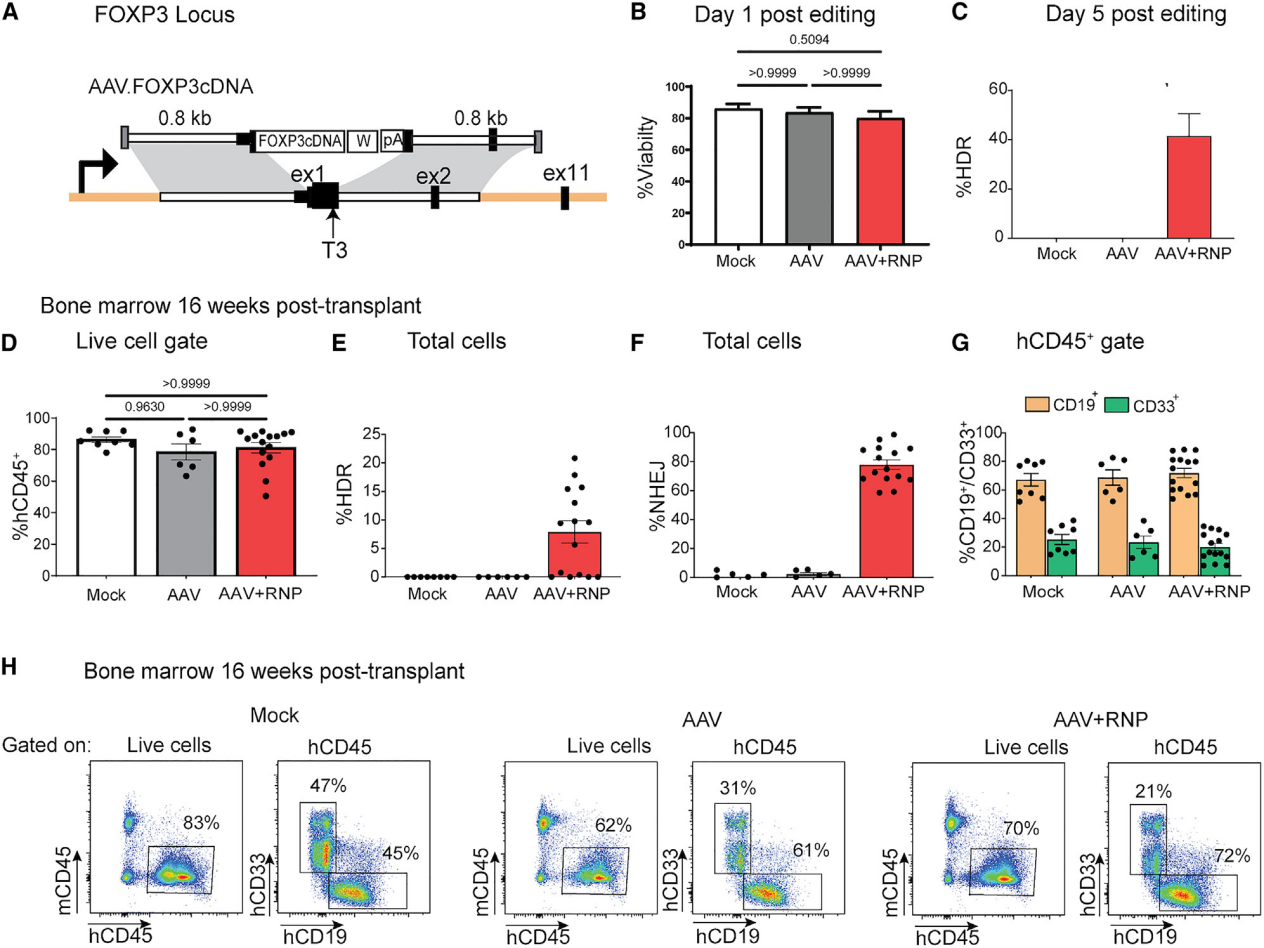
Long-term engraftment and differentiation of cDNA-edited cells in the BM of NBSGW mice after transplantation of HDR-edited HSPCs (A) Schematic of the *FOXP3* genomic locus and the rAAV6 targeting vector utilized to insert codon optimized FOXP3 cDNA at the endogenous start site of the *FOXP3* gene. FOXP3 cDNA, codon optimized cDNA sequence for FOXP3; pA, SV40 polyadenylation sequence; W, WPRE3 element. (B) Viability of HSPCs 1 day after editing with T3 RNP + AAV.FOXP3.cDNA using protocol B (n = 3 male donors, 4 independent studies). Significance determined by Kruskal-Wallis test. (C) HDR frequency determined by ddPCR of gDNA extracted from cells 5 days after editing with T3 RNP + AAV.FOXP3.cDNA (n = 3 male donors, 3 independent studies). (D) Engraftment (%hCD45^+^) in the BM of NBSGW recipient mice 16 weeks after transplant of mock (n = 8, 1 male donor, 1 female donor), AAV (n = 6, 1 male donor, 1 female donor), or AAV+RNP (n = 15, 1 male donor, 1 female donor) HSPCs cultured with protocol B from two independent studies. Significance determined by Kruskal-Wallis test. (E) HDR frequency determined by ddPCR of gDNA extracted from the BM of NBSGW recipient mice from (D). (F) NHEJ frequency determined by ddPCR of gDNA extracted from the BM of NBSGW recipient mice from (D). (G) Proportion of B cells (CD19^+^) and myeloid cells (CD33^+^) in the BM of NBSGW recipient mice from (D). (H) Representative flow cytometry data from BM of NBSGW recipient mice from (D). Bar graphs represent mean ± SEM.

We next modified our targeting AAV vector to enable insertion of a clinically relevant codon diverged FOXP3 cDNA at the endogenous start site of *FOXP3* (AAV.FOXP3cDNA) (Figure 3A). HDR rates observed upon editing mobilized CD34^+^ cells using T3 RNP increased in proportion to the dose of the AAV.FOXP3.cDNA template (100–2,200 MOI) (Figure S5B). No significant impact on cell viability was observed even at the highest AAV MOI evaluated (Figure S5A). Using these reagents and a MOI of 2,000, we achieved efficient HDR (average 42%) with minimal impact to cell viability (Figures 3B and 3C).

Mock, AAV.FOXP3.cDNA only, and AAV.FOXP3.cDNA plus RNP-treated CD34^+^ cells were transplanted into cohorts of NBSGW mice. Investigation of BM of mice sacrificed 16 weeks later revealed similar levels of average human cell chimerism—87% in mock, 79% in AAV only, and 83% in AAV plus RNP-treated transplanted mice (Figure 3D). Within the spleen, engraftment rates were 35%, 28%, and 24% for these groups, respectively (Figure S6A). Importantly, sustained HDR editing rates were observed within human cells recovered from BM (mean 8%; range, 0%–21%) and spleen (mean 5%; range, 0%–15%) (Figures 3E and S6B). We also determined the NHEJ rates in recipients of AAV plus RNP-treated cells, revealing high rates in both BM (78%) and spleen (79%) (Figures 3F and S6C). Minimal differences were observed in the percentage of CD19^+^ or CD33^+^ cells across groups, demonstrating that HDR editing with AAV.FOXP3.cDNA did not compromise *in vivo* differentiation potential (Figures 3G, S6D, and S6E).

### cDNA-edited HSPCs differentiate into T lymphocytes that maintain targeted integration

**Figure 4:**
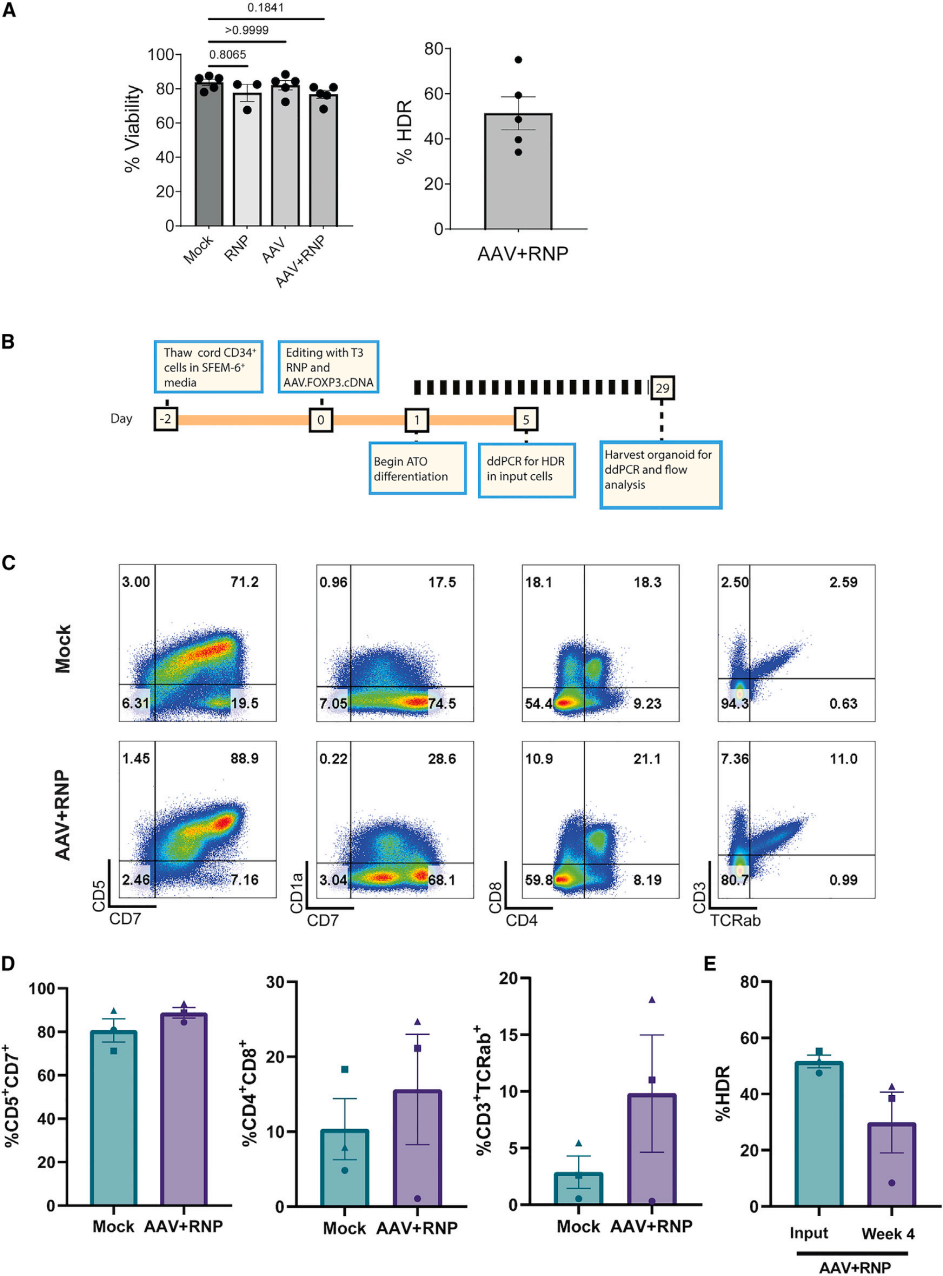
Efficient differentiation of FOXP3-edited HSPCs to T-lineage commitment (A) Viability (left) and HDR efficiency (right) in healthy donor CB CD34^+^ HSPCs mock treated (n = 4 male, 1 female), RNP treated (n = 3 male), AAV.FOXP3.cDNA-treated (n = 4 male, 1 female), or RNP + AAV.FOXP3.cDNA edited (n = 4 male, 1 female). Significance determined by Kruskal-Wallis test. (B) Timeline of CB CD34^+^ HSPC differentiation toward T lymphocyte lineage in artificial thymic organoid culture system. (C) Representative plots of mock-treated and edited CB CD34^+^ HSPC-derived cells after 28 days in ATO system. CD5 vs. CD7, CD1a vs. CD7, CD4 vs. CD8, and CD3 vs. TCRαß plots are gated by FSC vs. SSC, SSC-A vs. SSC-W, hCD45^+^ CD34^–^ and CD14^–^CD56^–^CD19^–^ to exclude monocytes, natural killer cells, and B cells. (D) Proportion of pre T-1 (CD5^+^CD7^+^), CD4^+^CD8^+^, and CD3^+^TCRab cells at the termination of ATO differentiation (gated on hCD45^+^CD34^–^ and CD14^–^CD56^–^CD19^–^) (n = 3 male donors, 2 independent studies). (E) Proportion of HDR-edited cells at the initiation and termination of ATO differentiation quantified by ddPCR (n = 3 male donors, 2 independent studies). Bar graphs represent mean ± SEM.

Next, we sought to determine whether cDNA edited CD34^+^ HSPCs retain the potential to develop into naive CD4 T cells, progenitors capable of differentiation into the T_reg_ cell lineage *in vivo* in response to critical differentiation cues. As the adult NBSGW model used in Figures 2 and 3 does not support robust T cell differentiation, we utilized two parallel differentiation strategies: OP9-DL1 co-culture^31^ and artificial thymic organoids^32^ to differentiate cDNA-edited human HSPCs toward T cell lineage *in vitro*. OP9-DL1 cells are stromal cells (derived from the M-CSF deficient op/op mouse) engineered to express the Notch ligand Delta-like1 (DL1) that can be used to facilitate differentiation of HSPCs into T lymphocytes in response to a combination of key cytokines and notch-dependent signals. As CB-CD34^+^ cells are more amenable to these differentiation protocols, we cultured and edited healthy donor CB-CD34^+^ in protocol B conditions. We observed cell viabilities comparable to mPB CD34 ^+^ 24 h after editing (Figure 4A, left). Notably, the average HDR editing rates were moderately higher in CB-CD34^+^ HSPCs than observed in mPB CD34^+^ HSPCs (Figure 4A, right). One day after editing, mock-treated and AAV.FOXP3.cDNA-edited CD34^+^ were put into differentiation systems as outlined in Figures 4B and S7A. At the same time, mock-treated and edited cells were analyzed by flow cytometry for surface expression of CD34, CD45, CD19, CD56, CD14, CD5, CD7, CD1a, CD3, and TCRαß to confirm CD34^+^ purity and the absence of contaminating T cells or other lin^+^ cells (Figure S7B). A small amount of mock-treated and edited cells were kept in culture until 5 days after editing to determine the HDR rate in edited cells that were introduced into the differentiation systems.

Cultures were maintained for 28 days then analyzed by flow cytometry for T lineage differentiation (Figures 4C and S7C). After 28 days in co-culture, differentiated cells predominantly reached CD34^–^CD5^+^CD7^+^ pre-T-1 stage (Figures 4D and S7D). Importantly, there was no observable differences between the proportion of pre-T-1 cells in mock-treated compared with edited conditions. Importantly, in the ATO system, we observed a proportion of cells reaching the CD4^+^ CD8^+^ double-positive and CD4^+^ or CD8^+^ single-positive developmental stages. In addition, we observed a modest amount of CD3^+^TCRab^+^ cells in comparable efficiency across mock-treated and AAV+RNP-treated conditions (Figure 4D), consistent with others finding of superior differentiation in the ATO system.^32^ Importantly, the proportion of HDR-edited cells at termination of OP9-DL1 differentiation closely matched the input HDR frequency (Figure S7E). We observed greater variation in the proportion of HDR-edited cells recovered after differentiation in the ATO system (Figure 4E). We believe the primary cause of high variability observed in the ATO system reflects the much lower number of input cells used (5,000) relative to OP9-DL1 (250,000) and potential skewing due to limited numbers of lymphoid progenitors and HDR-edited lymphoid progenitors in this smaller input sample.

Together, these results demonstrate that mock-edited and HDR-edited CB-CD34^+^ HSPCs exhibit similar T lineage differentiation capacity and that the overall proportion of AAV.FOXP3.cDNA HDR-edited cells remains stable during commitment to the T lineage.

### Assessment of potential off-target cleavage sites for FOXP3 sgRNA T3

The off-target sites for T3 guide were predicted *in silico* by CCtop-CRISPR-Cas9 target online predictor (Table S2). The top five off-target predicted sites were then interrogated using the Miseq platform. Two of the off-target sites (OT1 and OT4) were within the exons of genes. The top off-target site was Disheveled Binding Antagonist of Beta Catenin 2 (*DACT2*), a protein involved in intracellular signaling pathways during development. The OT4 site is located within the Exostosin Like Glycosyltransferase 1 gene (*EXTL1*), a member of the multiple exostoses family of glycosyltransferases involved in the chain polymerization of heparan sulfate and heparin. The off-target NHEJ rates at both OT1 and OT4 were 0.1%, equivalent to that observed for the mock sample that received no RNPs. The highest off-target cleavage (0.9%) was seen at the OT-II in Solute Carrier Family 2 Member 1 gene, which is the major glucose transporter in the mammalian blood-brain barrier (Figure S6A).

High rates of NHEJ were observed at the *FOXP3* locus (83%), which were equivalent to those determined by the ddPCR assay (94%). The majority of the NHEJ events at the FOXP3 locus were deletions (80%), followed by insertions (15%) and substitutions (10%) (Figures S8A and S8B). A high percentage of NHEJ edits were six nucleotide deletions that constituted 15% of the total NHEJ events (Figures S8C and S8D). Thus, the off-target cleavage rates were significantly lower at <1% for the five off-target sites, while high NHEJ edits were observed at the *FOXP3* locus, confirming that our sgRNAs is largely specific for the target locus.

### Efficient editing of IPEX patient CD34^+^ cells

**Figure 5:**
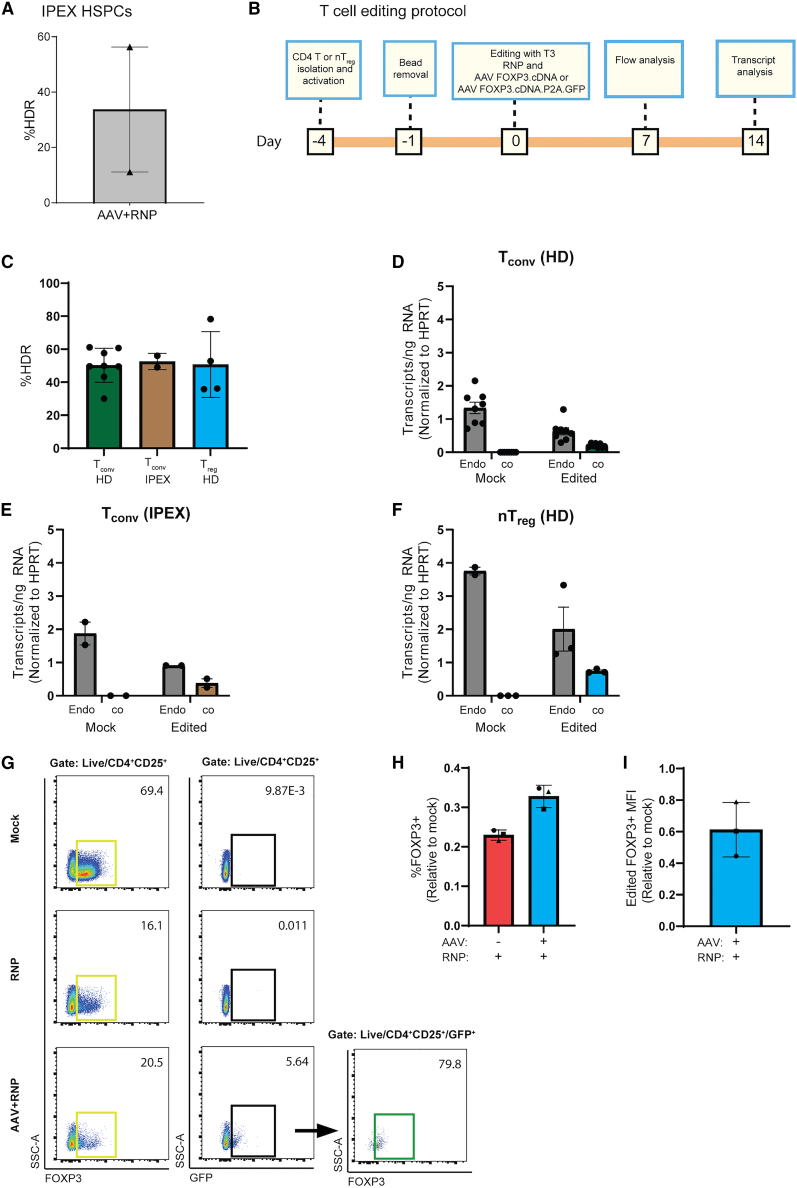
Molecular characterization of IPEX patient HSPCs and T_conv_ cells and healthy donor T_reg_ cells and T_conv_ cells edited with FOXP3 cDNA (A) HDR efficiency in RNP + AAV.FOXP3.cDNA edited IPEX patient HSPCS (n = 1 donor, 2 independent studies). (B) Timeline and protocol for T_reg_ cells and T_conv_ editing and characterization studies. (C) RNP + AAV.FOXP3.cDNA editing efficiencies in healthy donor-derived T_conv_ cells (n = 8 male donors, 6 independent studies), healthy donor-derived T_reg_ cells (4 male donors, 4 independent studies), and IPEX patient-derived T_conv_ (n = 2 male donors, 2 independent studies) quantified by ddPCR. (D–F) Endogenous (endo) and codon optimized (co) FOXP3 transcript levels in mock-treated and RNP + AAV.FOXP3.cDNA edited. (D) Healthy donor-derived T_conv_ cells (n = 8 male donors, 6 independent studies). (E) IPEX patient T_conv_ cells (n = 2 male donors, 2 independent studies). (F) Healthy donor-derived T_reg_ cells (n = 3 male donors, 2 independent studies) (transcripts quantified by ddPCR and normalized to HPRT control transcript). (G) Representative flow plots of FOXP3^+^ and GFP^+^ populations in nT_reg_ cells isolated from healthy donors. Comparison of unstained mock treated and FOXP3-stained mock-treated, RNP-treated, and RNP + AAV.FOXP3.cDNA.GFP-treated 7 days after editing. (H) Proportion of FOXP3 knockout (RNP-treated nT_reg_ cells) and FOXP3 restoration (RNP + AAV.FOXP3.cDNA.GFP edited) relative to mock treated. Gated on CD4^+^CD25^+^, representative plot highlighted in yellow in (G) (n = 3 male donors, 2 independent studies). (I) Mean fluorescence intensity (MFI) of edited FOXP3 (RNP + AAV.FOXP3.cDNA.GFP-treated nT_reg_ cells, gated on CD4^+^CD25^+^GFP^+^) compared with endogenous FOXP3^+^ MFI (mock-treated, gated on CD4^+^CD25^+^). Representative plot highlighted in green in (G). (n = 3 male donors, 2 independent studies). Bar graphs represent mean ± SEM.

We obtained a small CB specimen from an IPEX patient bearing an I363V missense mutation located in the FOXP3 forkhead domain that renders the protein incapable of establishing the T_reg_ cell transcriptional program. CD34^+^ cells were isolated from the frozen sample and editing reagents introduced after 2 days of culturing and pre-stimulation using protocol B. Notably, ddPCR analysis revealed average HDR editing rate of 34%, although significant differences were observed between the two studies performed, likely reflecting the limited cell numbers and relatively lower viability of the CB sample (Figure 5A).

### Characterization of FOXP3 cDNA-edited IPEX patient T cells and healthy donor natural T_reg_ cells

To evaluate expression of the FOXP3 cDNA cassette, we purified and edited healthy donor conventional T (T_conv_) cells, IPEX patient T_conv_ cells, and healthy donor natural T_reg_ (nT_reg_) cells following the experimental timeline outlined in Figure 5B and AAV.FOXP3.cDNA vector diagrammed in Figure 3A. Five days after editing, gDNA was extracted and HDR was quantified via ddPCR. HDR rates were similar across groups: T_conv_ cells (healthy) 50%, T_conv_ cells (IPEX) 52%, and nT_reg_ cells (healthy) 56% (Figure 5C). Fourteen days after editing, we extracted RNA, synthesized cDNA, and quantified transcript levels via ddPCR. Because we utilized a codon-diverged FOXP3 sequence in the AAV.FOXP3.cDNA vector, we were able to distinguish between endogenous and codon-diverged FOXP3 expression. As we achieved approximately 50% editing rates, we predicted approximately equal proportions of coFOXP3 and endoFOXP3 transcripts. However, in edited T_conv_ cells (healthy), T_conv_ cells (IPEX), and nT_reg_ cells (healthy) we measured a ratio of coFOXP3:endogenous FOXP3 transcripts of 0.32, 0.42, and 0.37 respectively (Figures 5D–5F). The reduced level of coFOXP3 transcripts in comparison with endogenous transcripts likely reflects differences in RNA processing efficiency of intron-less FOXP3 cDNA donor vs. the endogenous transcripts.

Next, we performed studies designed to identify FOXP3 protein expression mediated by the FOXP3 cDNA cassette following editing of an enriched nT_reg_ cell population. To specifically identify FOXP3 expression in HDR-edited nT_reg_ cells, we created a FOXP3.cDNA.P2A.GFP donor cassette where cis-linked GFP expression would permit precise flow-based identification of HDR-edited cells. Seven days after editing, we quantified GFP and FOXP3 levels in mock-treated, FOXP3 RNP (knockout), and FOXP3.GFP-edited nT_reg_ cells (Figure 5G). As predicted, GFP expression was observed only in the FOXP3.GFP-edited nT_reg_ cell population. We observed efficient (∼77%) FOXP3 knockout in the RNP-treated nT_reg_ cells and a low level of FOXP3 restoration in FOXP3.GFP-edited nT_reg_ cells (Figure 5H). Importantly, within the GFP^+^ population in the FOXP3.GFP-edited nT_reg_ cell population, the majority of cells were FOXP3^+^; and this population exhibited approximately 60% the mean fluorescence intensity of wildtype FOXP3 (Figure 5I). We attempted to perform suppression assays using fluorescence-activated cell sorting (FACS)-sorted GFP^+^ populations. However, due to limited cell yields and purity loss during expansion, we did not obtain sufficient viable GFP^+^ T_reg_ cells for functional assays.

Together, these data demonstrate *FOXP3* promoter-mediated transcription of coFOXP3 in HDR-edited nT_reg_ cells from healthy control subjects and T_conv_ cells isolated from both IPEX patients and healthy control subjects; we also directly demonstrate expression of exogenous FOXP3 protein in HDR-edited healthy control nT_reg_ cells, albeit at sub-endogenous levels.

## Discussion

IPEX syndrome, caused by *FOXP3* mutations, is a devastating disease that leads to substantial mortality. The requirement for strict epigenetic regulation of *FOXP3* necessitates development of a therapeutic approach that preserves the endogenous control elements required to orchestrate both thymus-dependent T_reg_ cell lineage differentiation and maintenance of the T_reg_ cell program *in vivo*. One approach for the treatment of IPEX is enforced expression of FOXP3 in CD4^+^ T cells by delivering its coding sequence driven via a robust promoter.^19^ While such autologous engineered T_reg_ cells may provide temporary clinical benefit, replicating the critical steps in Treg cell lineage programming and selection and generation over time requires thymic repopulation with gene-corrected lymphoid progenitors derived from LT-HSC. Thus, to achieve a cure for IPEX necessitates editing of CD34^+^ HSC and seamless introduction of the FOXP3 cDNA under transcriptional control of the endogenous promoter. Through optimization of CD34^+^ culture and HDR editing protocols, we demonstrate efficient insertion of FOXP3 cDNA at the *FOXP3* locus and sustained engraftment of cDNA-edited cells *in vivo*. We show that this approach is feasible using clinically relevant, mobilized CD34^+^ HSPCs from multiple healthy donors as well as in CD34^+^ cells derived from an IPEX subject. Further, we show that cDNA-edited CD34^+^ cells retain the capacity to differentiate into T lineage cells and retain the FOXP3 cDNA cassette *in vitro*. Finally, using healthy donor and IPEX patient T lymphocytes edited with the FOXP3 cDNA cassette, we find FOXP3 cDNA transcripts and FOXP3 protein is expressed at levels that may provide a selective advantage *in vivo*, similar to the expansion of donor-derived T_reg_ cells observed in IPEX patients following non-myeloablative HSCT.^15^^,^^16^^,^^17^^,^^18^

Culturing and editing conditions can have a significant impact on the HDR rates and engraftment potential of HSPCs. Culturing CD34^+^ cells using a low-density editing protocol and small molecules that support HSC survival (protocol B) enabled higher rates of HDR in the edited cells (>40%), while no differences were observed in the rates of NHEJ edits between the two protocols. Upon transplantation of edited cells into humanized mice, a 2-fold higher engraftment of HDR-edited cells was observed with protocol B compared with protocol A edited cells. A similar low-density protocol was previously reported to improve HDR rates within the LT-HSC population by enforcing G2M or S phases of the cell cycle and facilitated higher engraftment of edited cells *in vivo*.^27^ We also observed a 2-fold higher percentage of HSC-enriched HSPCs and a 3-fold higher proportion of HDR-edited (GFP^+^) cells within the HSC-enriched HSPC gate recovered from mice further corroborating this hypothesis. Normal lineage distribution was observed with cells edited with either protocol compared with mock controls, suggesting that editing did not negatively impact differentiation *in vivo*.

Recently reported methods to improve editing and engraftment of HDR-edited LT-HSCs include co-transfection of select mRNAs during nuclease delivery. Ferrari et al.^33^ have shown improved engraftment of gene-edited LT-HSCs by introduction of dominant negative P53 inhibitor along with adenoviral protein Ad5-E4orf6/7. Transient expression of Ad5-E4orf6/7 triggered an E2F-driven pleiotropic response that facilitated cell-cycle progression and expression of genes encoding for the HDR apparatus leading to increased editing within primitive CD34^+^ cells. In parallel, the dominant active P53 inhibitor (GSE56),^34^ helped to preserve HSC survival or engraftment upon transplantation in immune deficient mice. While not tested in human HSC, another possible approach to enhance HDR is fusion of dominant-negative mutant of 53BP1 to Cas9.^35^ 53BP1 enhances HDR by limiting DNA end resection and hindering recruitment of *BRCA1* to the DNA cleavage site.^36^^,^^37^ By fusing Cas9 activity with 53BP1 inhibition, the authors were able to locally retard NHEJ at the site of the introduced DSB without causing global 53BP1 inhibition. In another study, co-delivery of an engineered ubiquitin variant of an inhibitor of 53BP1 (i53)^38^ mRNA and GSE56^34^ mRNA with editing reagents enhanced long term correction in X-MEN patient BM-derived CD34^+^ cells.^39^ Robust engraftment of HDR-edited patient cells was observed in the BM of intrahepatic-transplanted, irradiated, newborn NSGS mice in a modulator-dependent manner. While effectively demonstrating modulator efficacy, it remains unclear whether this model accurately predicts HDR-edited HSPC engraftment capability in a clinical setting.

To validate expression and assess the levels of the introduced codon optimized transcript, we edited primary T lymphocytes and nT_reg_ cells from healthy donors. Expression of codon optimized transcripts was readily detected in both HDR-edited T cells and T_reg_ cells. Exogenous cDNA expression was highest in HDR edited T_reg_ cells likely due to the open chromatin landscape in the region compared with conventional CD4^+^ T cells.^40^^,^^41^ Our HDR editing methodology also performed similarly using CD4^+^ T lymphocytes derived from two independent IPEX subjects including CB T cells (isolated from a subject with a I363V mutation) and peripheral blood CD4^+^ T (from a subject with a polyA region mutation). Despite efficient editing in both healthy control and IPEX patient-derived T cells, exogenous cDNA expression levels comprised between 32% and 42% of wildtype FOXP3. Consistent with our findings, in a separate study by Goodwin et al.,^20^ introduction of a FOXP3.cDNA.LNGFR cassette into T_reg_ cells, resulted in sub-endogenous levels of FOXP3 protein expression in edited T_reg_ cells compared with control T_reg_ cells. Lower or absent protein expression has been reported when fully spliced cDNA is introduced into the first coding exon of a target gene.^20^^,^^42^ This approach precludes the splicing process, often required for optimal transcription and translation. Intronic sequences harbor regulatory elements and their interaction with the splicing machinery can play a critical role in modulating initiation/processivity by RNA polymerase II, pre-mRNA processing, and/or mRNA export.^43^ We speculate that, by redesigning AAV HDR cDNA donors to include alternate post-transcriptional elements such as the full WPRE element, a stronger polyadenylation signal and/or candidate intronic elements, that codon optimized FOXP3 transcript and protein expression will reach endogenous expression levels. Such modifications in donor design are likely to be required to achieve consistent T_reg_ cell function.

Investigation of the FOXP3 T3 sgRNA cut site using NGS revealed the indel spectrum in edited cells. Larger deletions (>3 nucleotides) were favored over fewer than three nucleotide deletions which accounted for only 6% of the total NHEJ events. The indel signature of sgRNAs has been utilized by Tatiossian et al.^44^ to predict the outcome of HDR frequency and further corroborated using donor templates demonstrating that a larger proportion of nucleotide deletions of more than three nucleotides favor HDR upon template introduction, as was seen with this specific sgRNA. Analysis of the top five predicted off-target sites revealed less than 1% off-target DSBs. Moving the FOXP3 T3 sgRNA toward therapeutic application, however, will necessitate additional unbiased off-target assessments such as GUIDE-seq or alternative methodologies.^45^

An HDR editing strategy similar to that described in our study was utilized to target *FOXP3* locus in T cells (from healthy and IPEX donors) and healthy donor CB-derived HSPCs using CRISPR sgRNAs and rAAV6 vectors.^20^ Compared with our findings, Goodwin et al.^20^ demonstrated a lower level of engraftment of HDR edited CB progenitors. Consistent with this limited engraftment, purified edited vs. unedited T cells derived from engrafted animals failed to demonstrate suppressive activity *in vitro*. In contrast, we focused primarily on editing and transplantation of HSPCs derived from apheresis of G-CSF-mobilized healthy donors—the HSC cell source anticipated to be utilized for clinical application. In parallel, as in Goodwin et al.,^20^ we demonstrate successful HDR editing in T cells from both healthy and IPEX donors. Importantly, here we also demonstrate successful editing of CD34^+^ cells from IPEX patients. As an alternative therapeutic approach, LV-mediated gene delivery of a FOXP3 expression cassette (utilizing the proximal *FOXP3* promoter and conserved non-coding sequences and FOXP3 cDNA) into murine HSCs, followed by transplantation of purified T cells into neonatal *scurfy* mice (the murine equivalent of IPEX) was shown to limit disease.^9^ However, heterogeneous expression correlating with viral copy number (VCN) was observed and high VCNs (>3) were required to reach a therapeutic threshold of transgene expression. Further, the critical conserved non-coding sequence 2 within the LV sequences did not retain endogenous methylation dynamics. Finally, LV gene therapy has other potential disadvantages including inability to control VCN or integration site, position-effect variegation, and the potential of insertional mutagenesis.

In summary, we demonstrate efficient HDR-based editing of the *FOXP3* locus in control and IPEX patient CD34^+^ HSPCs. Further, we show that control HDR-edited HSPCs are capable of sustained engraftment *in vivo* in humanized mice. This editing methodology sets the foundation for developing a definitive therapy for IPEX patients. Incorporation of recent advances in the field and additional HDR donor design changes will likely assist in improving outcomes and paving the way for clinical translation.

## Materials and methods

Reagent source and category numbers listed in Tables S3–S10 and rAAV6 sequences are listed in Table S11.

### Experimental model and subject details

#### Cell lines

##### OP9-DL1 cells

The OP9-DL1 stromal cells were cultured in Alpha MEM with Nucleosides (STEMCELL Technologies, Vancouver, Canada) supplemented with 20% fetal bovine serum (FBS) (Omega Scientific, Tarzana, CA, USA).

##### MS5-hDLL4 cells

MS5 murine stromal cells transduced with a lentiviral vector encoding human DLL4 were a provided by Dr. Gay Crooks (UCLA). Stable expression of DLL4 was confirmed by flow cytometry after multiple weeks in culture. MS5-DLL4 cells were cultured in DMEM (Gibco, Thermo Fisher Scientific, Waltham, MA, USA) + 10% FBS (Omega Scientific).

#### Primary cells

##### Mobilized peripheral HSPCs and CB CD34^+^ cells from healthy donors

Human CD34^+^ HSPCs enriched from mobilized PBMCs were obtained from the Cooperative Centers of Excellence in Hematology, Fred Hutchinson Cancer Research Center (supported by NIDDK Grant DK106829). CB from healthy donors was purchased from Bloodworks Northwest (Seattle, WA); CD34^+^ cells were isolated from CB using human CD34 MicroBead Kit (Miltenyi Biotec, Bergisch Gladback, Germany).

##### PBMC-derived CD4^+^ T lymphocytes and tT_reg_ cells from healthy donors

PBMCs were obtained from the Cooperative Centers of Excellence in Hematology, Fred Hutchinson Cancer Research Center. Human primary CD4^+^ T cells and tT_reg_ cells were isolated from thawed PBMCs using negative selection for CD4 and positive selection for CD4^+^CD127^low^CD25^+^ enrichment, respectively (both from STEMCELL Technologies). T cells were cultured in T cell media (RPMI 1640; Gibco) with 20% FBS (Omega Scientific), 10 mM HEPES (Gibco), 2 mM Glutamax (Gibco), 55 μM β-mercaptoethanol (Sigma-Aldrich, St. Louis, MO, USA) supplemented with IL-2 (50 ng/mL, Peprotech, Thermo Fisher Scientific).

#### IPEX patient samples

CB samples from IPEX patient with I363V mutation and PBMCs from IPEX patient with a polyA mutation (AAUAAA>AAUGAA within the endogenous poly A sequence) were obtained after informed consent using protocols approved by the institutional Review Board of Seattle Children’s Research Institute.

#### Mouse strains

NBSGW mice (NOD.Cg-*Kit*^*W−41J*^*Tyr*^+^*Prkdc*^*scid*^*Il2rg*^*tm1Wjl*^/ThomJ, Stock 026622, Jackson Laboratory, Bar Harbor, ME, USA) used for the experiments were either purchased from Jackson Laboratory or inbred and maintained in the specific pathogen-free animal facility of the Seattle Children’s Research Institute according to Institutional Animal Care and Use Committee and approved protocols.

### Method details

#### sgRNA selection

CRISPR gRNAs targeting *FOXP3* exon 1 were identified using CCTop- CRISPR-Cas9 target online predictor (https://crispr.cos.uni-heidelberg.de/). Top cleaving sgRNAs (T3 and T9) identified from an initial screen were selected for testing in HSPC CD34^+^ cells. The guides were synthesized as chemically modified 2′-O-methyl analogs with 3′ phosphorothioate internucleotide linkages in the first three 5′ and 3′ terminal residues (Synthego, Redwood City, CA, USA).

#### AAV6 donor templates and vector production

In-Fusion HD cloning kit (Takara, Kusatsu, Japan) was used to insert PCR amplified fragments into pAAV.GFP (a gift from John T. Gray, Addgene plasmid #32395) replacing the GFP and α-globin polyadenylation site in the original vector. T3 and T9 FOXP3.MND.GFP-targeting vectors contain the MND promoter^46^ upstream of a GFP cDNA, followed by WPRE3^47^ and SV40 polyadenylation signal elements; this expression cassette was flanked 5′ and 3′ by 0.6 or 0.8 kb *FOXP3* homology arms. The FOXP3.cDNA vector contains the codon-optimized FOXP3 cDNA cassette followed by WPRE3 and SV40pA, flanked by 0.8 kb *FOXP3* homology arms on either side. The FOXP3 cDNA.P2A.GFP vector contains the codon-optimized FOXP3 cDNA cassette followed by a P2A ribosomal skip sequence followed by a promoter-less GFP cDNA sequence with WPRE3 and SV40pA elements. The FOXP3 cDNA.P2A.GFP contains the same 0.8-kb homology arms as the FOXP3 cDNA construct.

AAV6 stocks were produced by transient transfection of HgT1-Adeno, Repcap6,^48^ and vector plasmid into HEK 293T cells as previously described.^49^ Briefly, 48 h after transfection of the vector and helper plasmids, the cells were harvested, pelleted, and frozen thawed three times. The lysate was then treated with benzonase nuclease, loaded onto an iodixanol density gradient and subjected to ultracentrifugation at 67,000 g in Ti70 rotor (Beckman Coulter, Brea, CA, USA). The virus was extracted from the 60%–40% iodixanol interface, aliquoted, and stored at −80°C. The titers of the AAV stocks were determined by qPCR using primers and probes specific for the viral inverted terminal repeats.^50^

#### CD34^+^ cell culture and editing

For culturing CD34^+^ cells using protocol A, cells were seeded into six-well tissue-culture plates at the density of 1 × 10^6^ cells/mL in HSC-6^+^ media composed of CellGenix GMP SCGM media (CellGenix, Sartorius, Gottingen, Germany) with 100 ng/mL each of the following recombinant human cytokines: thrombopoietin, stem cell factor, FLT3 ligand, and IL-6 (all from PeproTech), at 37°C, 5% CO_2_, and 5% O_2_. Forty-eight hours later, 2 × 10^5^ CD34^+^ cells were electroporated with ribonucleoprotein (RNP) complexes containing 7.5 pmol Cas9 and 13.8 pmol sgRNA, using either a Neon transfection system (Life Technologies, Thermo Fisher Scientific) or Lonza 4-D nucleofector (Lonza, Basel, Switzerland). The cells were transferred to pre-warmed media at the density of 0.8 ×10^6^ cells/mL following electroporation and AAVs added at MOIs ranging from 0.1 to 2K viral genomes (vg)/cell.

For protocol B, cells were seeded at a density of 0.25 × 10^6^ cells/mL in SFEM-6^+^ media (SFEMII as basal media supplemented with the same cytokines as protocol A plus 1 μM StemRegenin1 [STEMCELL Technologies] and 35 nM UM171 [ApexBio, Houston, TX, USA]). Forty-eight hours later, RNPs were nucleofected into 2 × 10^5^ cell using Lonza 4-D nucleofector (Lonza) at the same concentration as protocol A. The cells were plated at 1 × 10^6^ cells/mL concentration post nucleofection and transduced with AAV at MOIs ranging from 0.1 to 2K vg/cell. Sixteen hours after transfection, cells were diluted to a density of 2.5 × 10^5^ cells/mL.

The cells were cultured for 5 days after dual delivery of RNPs and AAV. SpyFi Cas9 (Aldevron, Madison, WI, USA) nuclease was used in both protocols. Flow cytometry analysis was performed 1, 2, and 5 days after editing, following which the cells were pelleted and gDNA extraction performed using Qiagen Dneasy Blood and tissue Kit (Qiagen, Hilden, Germany). Editing was performed using the same protocol as above for IPEX I363V cord-derived CD34^+^ cells.

#### Primary and patient-derived CD4^+^ T and nT_reg_ cell culture and editing

Human primary CD4^+^ T cells and nT_reg_ cells isolated from thawed PBMCs as described above were activated with Dynabeads Human T-expander beads CD3/CD28 (Gibco) at a 3:1 bead to cell ratio for 72 h. After beads were removed, the cells further rested overnight in T cell media followed by nucleofection of 20 pmol Cas9 and 50 pmol sgRNA complexes using Lonza 4D-nucleofector. Donor AAVs were added to the cultures immediately post nucleofection at 15%–20% of the culture volume. After an approximately 24-h incubation at 37°C, fresh media was added to cultures to dilute the AAV to 7.5%–10% of the culture volume. Cells were then split every 2–3 days until day 14 after editing. CD4^+^ T cells from a FOXP3 poly A mutation and IPEX I363V IPEX patients were also edited as described above for healthy donors.

For nT_reg_ cell phenotyping, cells were surface stained for flow cytometry with the following antibodies: CD4-BV605, CD25-PECy7, and CD127-BV510. Intracellular FOXP3 staining with FOXP3-PE antibody was performed after fixation and permeabilization with True-Nuclear Transcription Factor buffer set (BioLegend, San Diego, CA, USA).

#### ddPCR analysis for determination of NHEJ rates

PCR amplicons spanning the guide cleavage site were generated with the NHEJ probe binding to the guide cleavage site. A control amplicon of similar size was generated from another region of the *FOXP3* gene. The PCR reactions were partitioned into droplets using a QX200 Droplet Generator (Bio-Rad, Hercules, CA, USA). Amplification was performed using ddPCR Supermix for Probes without UTP (Bio-Rad), 900 nM of primers (IDT, Coralville, IA, USA), 250 nM probe (IDT), and 50 ng genomic DNA. Droplets were analyzed using the QX200 ddPCR System (Bio-Rad) and analyzed using QuantaSoft software (Bio-Rad). The NHEJ rates were calculated using the formula:

$${(\left(\text{signal}\;\text{from}\;\text{NHEJ}\;\text{probe}/\text{signal}\;\text{from}\;\text{control}\;\text{probe}\right)\;}_{\text{mock}\;\text{sample}}{-\;\left(\text{signal}\;\text{from}\;\text{NHEJ}\;\text{probe}/\text{signal}\;\text{from}\;\text{control}\;\text{probe}\right)\;}_{\text{RNP}\;\text{treated}\;\text{sample}})*100\text{.}$$

#### ddPCR analysis for determination of targeted integration

Genomic DNA was extracted from cultured cells and an HDR amplicon was generated by in-out ddPCR using one primer within the AAV construct and another outside the region of homology. An amplicon for either *ActB* (1.3 kb) or *CCR5* (1.5 kb) was generated to serve as the control. Probes for both amplicons were labeled with FAM and the reactions were performed in separate wells. The ddPCR was performed as described in the section above.

#### RNA extraction and transcript analysis in edited CD4^+^ T lymphocytes

RNA was extracted using RNeasy mini kit (Qiagen) from cultured T lymphocytes 14 days after editing. Complementary DNA was synthesized using Maxima First Strand cDNA Synthesis Kit (Thermo Fisher Scientific) utilizing 10 ng input RNA. Two microliters cDNA was used in three separate ddPCR reactions to detect codon optimized FOXP3, endogenous FOXP3, and control HPRT transcripts using in-house designed or Taqman gene expression assays (Thermo Fisher Scientific). All reactions were performed in duplicates. Transcript concentration was quantified with the following formula:

$${coFOXP3}_{Mock,Treg}=\frac{{coFOXP3}_{Mock,Treg}}{{HPRT}_{Mock,Treg}}$$

$${Endogenous\;FOXP3}_{Mock,Treg}=\frac{{Endogenous\;FOXP3}_{Mock,Treg}}{{HPRT}_{Mock,Treg}}$$

#### Xenotransplantation of edited CD34^+^ cells into NBSGW mice

NBSGW mice (NOD.Cg-*Kit*^*W−41J*^ *Tyr* ^+^ *Prkdc*^*scid*^ *Il2rg*^*tm1Wjl*^/ThomJ, Stock 026622, Jackson Laboratory) used for the experiments were either purchased from Jackson Laboratory or inbred and maintained in the specific pathogen-free animal facility of Seattle Children’s Research Institute according to Institutional Animal Care and Use Committee and approved protocols. Mock-treated or edited HSPCs treated with either protocol A or B were transplanted to NBSGW recipient mice one day after editing. The recipient mice were treated with 12.5 mg/kg clinical grade Busulfan (Otsuka America Pharmaceutical, Rockville, MD, USA) intraperitoneally 24 h prior to human stem cell transfer followed by retro-orbital injections of 1–2 × 10^6^ CD34^+^ cells per animal. The transplanted mice were sacrificed 12–16 weeks after transfer, and cells harvested from BM and spleens were analyzed using flow cytometry on LSR II flow cytometer (BD Biosciences, San Jose, CA, USA). To assess engraftment of edited cells in various hematopoietic lineages within the BM and spleen, cells were stained with the following fluorophore-conjugated antibodies: human and mouse CD45, CD33, and CD19. To assess the HSC phenotype, cells were stained with the following fluorophore-conjugated antibodies: CD34, CD38, CD90, and CD133.

#### *In vitro* differentiation of cord-derived CD34^+^ cells in OP9-DL1 monolayer

CB CD34^+^ cells were thawed and edited as described previously for adult mobilized CD34^+^ cells. One day post-editing, 250,000 CD34^+^ cells were cocultured on confluent OP9-DL1 stromal cells in Alpha MEM with Nucleosides (STEMCELL Technologies) supplemented with 20% FBS and recombinant human cytokines (Peprotech) IL-7 (10 ng/mL) and FLT3L (10 ng/mL). Input cells were simultaneously analyzed for surface expression of hCD45, CD34, CD14, CD56, CD19, CD1a, CD7, CD3, TCRαß, CD4, and CD8. We kept 150,000 CD34^+^ in stem cell media and gDNA was extracted 5 days to quantify input cell HDR by ddPCR. CD34^+^ co-cultures were re-plated onto fresh stromal cells every 3–4 days. After 28 days of co-culture, the cord-derived cells were analyzed for surface expression of hCD45, CD34, CD14, CD56, CD19, CD1a, CD7, CD3, TCRαß, CD4, and CD8. Genomic DNA was extracted from culture and targeted integration of FOXP3 cDNA was quantified by ddPCR.

#### *In vitro* differentiation of cord-derived CD34^+^ cells in ATO system

CB CD34^+^ cells were thawed and edited as described previously for adult mobilized CD34^+^ cells. One day after editing, 5E4 CD34^+^ were differentiated in artificial thymic organoid as previously described.^32^ Input cells were simultaneously analyzed for surface expression of hCD45, CD34, CD14, CD56, CD19, CD1a, CD7, CD3, TCRαß, CD4, snf CD8. CD34^+^ cells were also kept in stem cell media and gDNA was extracted 5 days after editing to quantify input cell HDR by ddPCR. After four weeks, ATO cultures were harvested and analyzed by FACs for T lineage differentiation and ddPCR for HDR.

#### Off- and on-target cleavage validation using Miseq

Off-target cleavage sites for guide T3 were determined using CCTop- CRISPR-Cas9 target online predictor. The top five predicted off target sites (Table S2) along with the target *FOXP3* site were amplified using 200 ng input DNA from two edited donor CD34^+^ cells using MiSeq oligos and PrimeSTAR GXL DNA polymerase (Clontech, Takara). The above-mentioned amplifications were also performed on donor CD34^+^ in parallel without delivering any editing reagents to serve as a control. The samples were purified using Agencourt AMPure XP (Beckman Coulter) and analyzed on PAGE gel. The samples were quantified on Qubit (Thermo Fisher Scientific), pooled and analyzed on MiSeq 500 CycleV2 kit (Illumina, San Diego, CA, USA). Data mining was performed with Crispresso2 algorithm.^51^

### Quantification and statistical analysis

Statistical analysis was performed using GraphPad Prism software (GraphPad).

## Data and code availability

Materials described here will be provided upon request upon execution of a material transfer agreement with Seattle Children’s Research Institute. Human cells will not be provided. Data generated and analyzed in this work are available from the corresponding author on reasonable request.

## Supplemental information

Document S1. Figures S1–S8 and Tables S1–S10

Table S11. rAAV6 sequences

Document S2. Article plus supplemental information
