# Supplementary material for: Efficient and sustained FOXP3 locus editing in hematopoietic stem cells as a therapeutic approach for IPEX syndrome
Source: Mol Ther Methods Clin Dev. 2023 Dec 26;32(1):101183. doi: 10.1016/j.omtm.2023.101183 (PMC10818254; doi:10.1016/j.omtm.2023.101183)
Supplement: Document S1. Figures S1–S8 and Tables S1–S10 [file mmc1.pdf]

OMTM, Volume *32*

Supplemental information

Efficient and sustained *FOXP3* locus editing in hematopoietic stem cells as a therapeutic approach for IPEX syndrome

Swati Singh, Cole M. Pugliano, Yuchi Honaker, Aidan Laird, M. Quinn DeGottardi, Ezra Lopez, Stefan Lachkar, Claire Stoffers, Karen Sommer, Iram F. Khan, and David J. Rawlings

Supplemental Materials

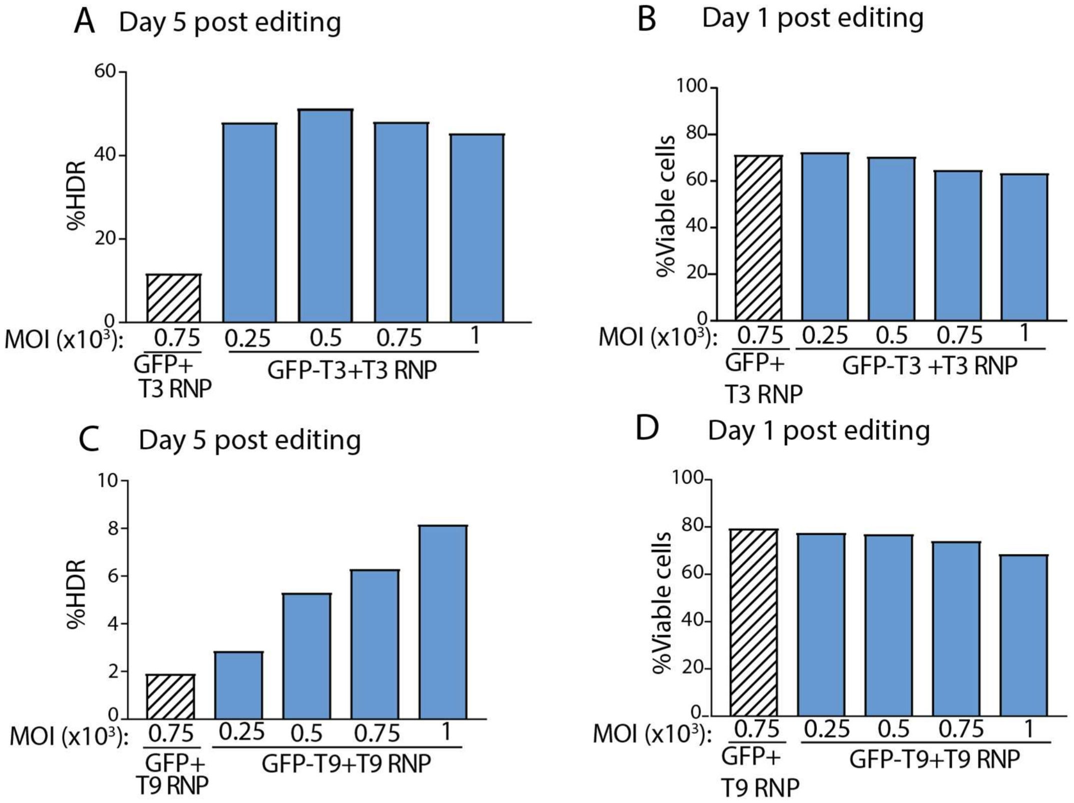

Figure S1. Improved editing rates with gRNA specific AAV donor templates. (A, B) Bar graphs depicting HDR rates 5 days post-editing **(A)** or cell viabilities determined by forward and side scatter one day post editing **(B)** using T3 RNP along with the common AAV.GFP donor (black striped bars) or the T3 specific AAV (GFP-T3; blue bars) at the indicated MOIs. The common AAV.GFP donor harbored a 0.2 kb deletion to disrupt the binding sites for both sgRNAs. **(C, D)** Bar graphs depicting HDR rates five days post-editing **(C)** or cell viabilities determined by forward and side scatter one day post editing **(D)** using T9 RNP along with the common AAV.GFP donor (black striped bars) or the T9 specific AAV (GFP-T9; blue bars) at the indicated MOIs.

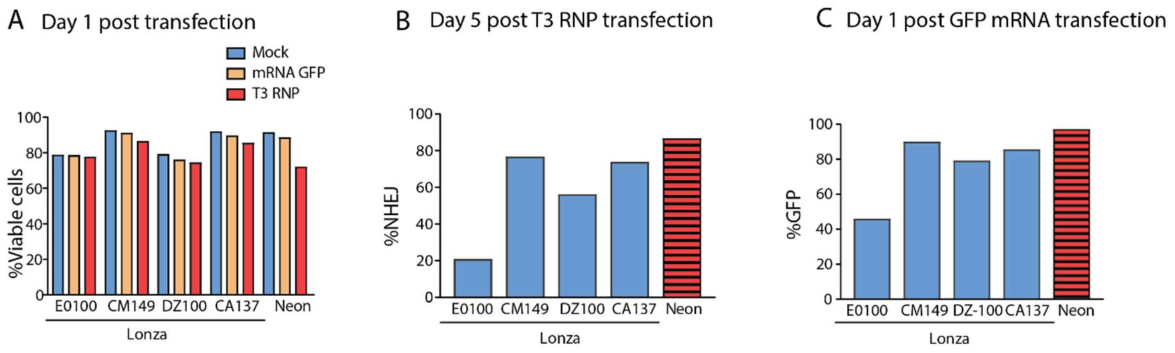

Figure S2. Comparison of cell viabilities and transfection efficiencies when nucleofecting human CD34⁺ cells with Lonza or electroporating with Neon. Adult CD34⁺ cells were cultured in HSC media for 48 hours using protocol A, followed by electroporation using Neon or nucleofection with Lonza. The cells were either mock transfected, transfected with 1µg of GFP mRNA or with T3 RNP. Program CM149 allowed highest transfection of GFP mRNA in greater than 90% of the cells without any impact on cell viability and was thus employed for all future nucleofections. **(A)** Bar graphs show cell viability one day post transfection as assessed by flow cytometry forward and side scatter. **(B)** Bar graphs showing % NHEJ assessed by ddPCR of gDNA from CD34⁺ cells 5 days post transfection with T3 RNP. **(C)** Bar graphs showing % transfection assessed by flow cytometry one day post-delivery of GFP mRNA.

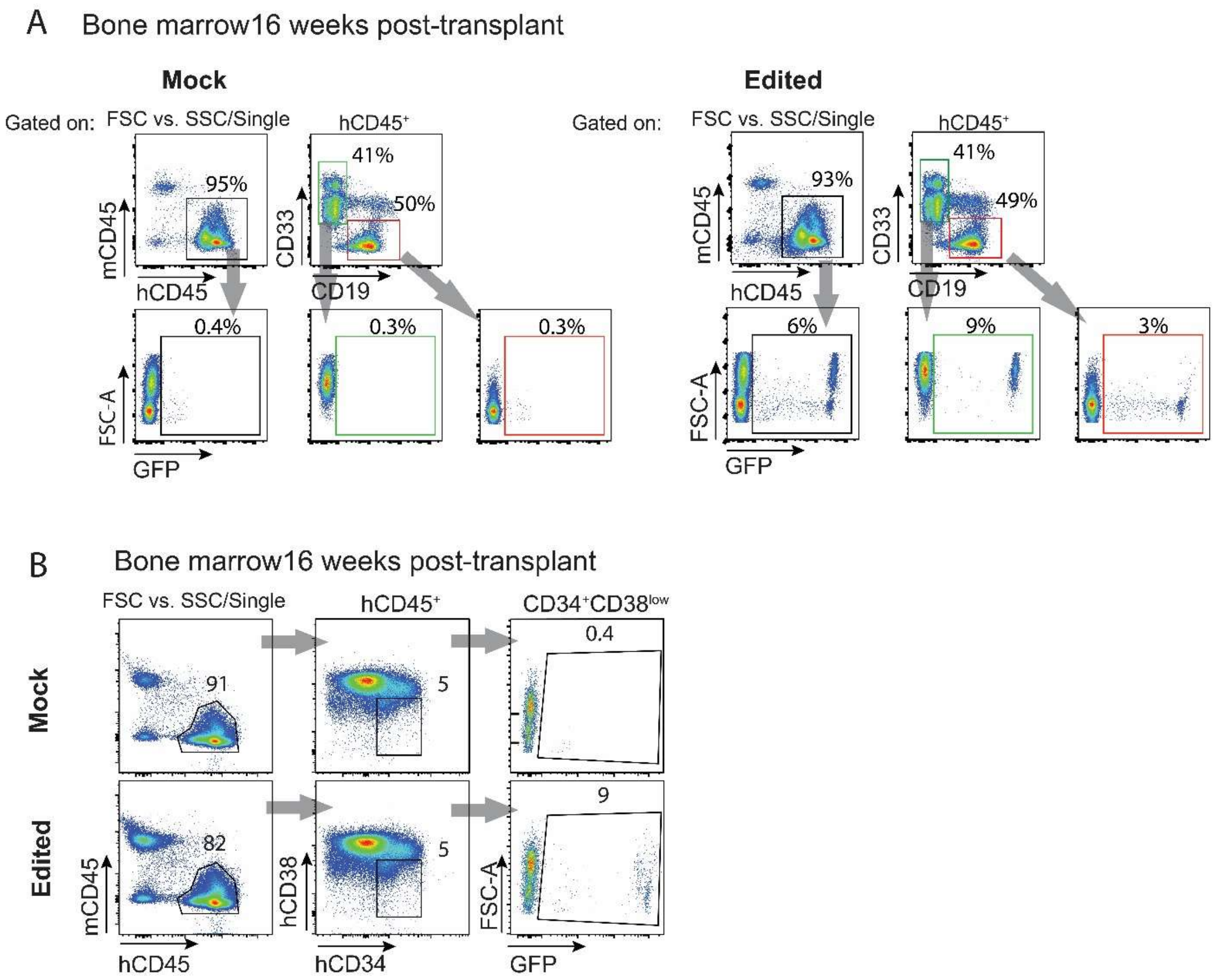

Figure S3. Gating strategy for analysis of bone marrow in GFP-T3 in vivo studies (Figure 2). **(A)** Gating strategy in mock-treated (left) and edited (right) animals to quantify human chimerism (Figure 2B) and B cell/myeloid compartments (Figure 2D). HDR frequency (GFP+) in bulk hCD45+ (Figure 2C), B cell (Figure 2E), and myeloid (Figure 2F) compartments shown with arrows drawn from parental gate. hCD45 vs. mCD45 was first gated on FSC vs. SSC and SSC-A vs. SSC-W, CD19 vs. CD33 was gated from hCD45+. **(B)** Gating strategy in mock-treated (top) and edited (bottom) animals to quantify engraftment of HSPCs (CD34+CD38low) (Figure 2G) and HDR frequency in HSPCs (Figure 2E). hCD45 vs. mCD45 was first gated on FSC vs. SSC and SSC-A vs. SSC-W, CD34 vs. CD38 was gated on CD45+, and GFP+ was gated on CD34+CD38low.

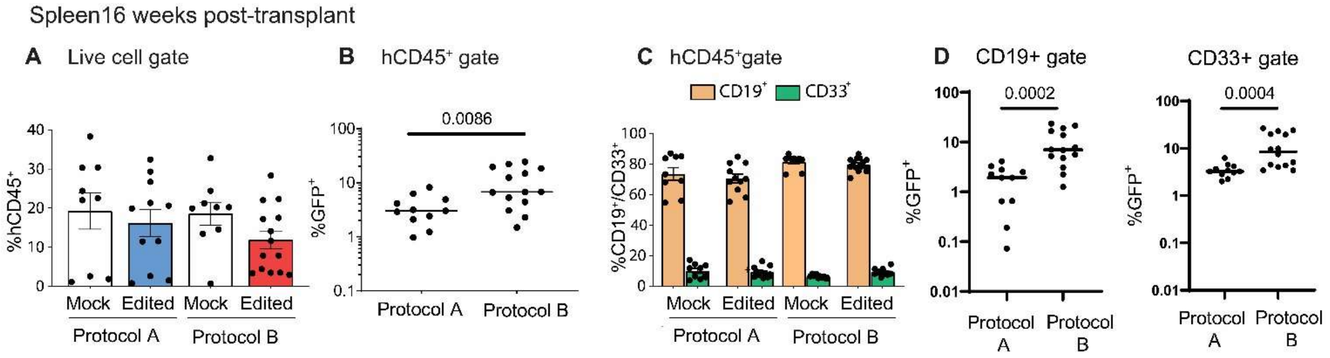

Figure S4. Long-term engraftment of GFP-T3 edited HSPCs in the spleen of NBSGW mice. **(A)** Bar graphs showing hCD45+ engraftment in spleens of NBSGW mice, 12-16 weeks post adoptive transfer of mock or edited CD34+ cells cultured using protocol A or B. Mean +/- SEM. **(B)** HDR frequency in bulk (hCD45+) cells recovered from spleens of NBSGW mice. Mann-Whitney U-test Median. **(C)** Bar graphs depicting distribution of B (CD19+) and myeloid (CD33+) cells within human CD45+ cells recovered from mice. Mean +/- SEM. **(D)** HDR frequency in B cell (left) or myeloid (right) compartments recovered from spleens of NBSGW mice. Mann-Whitney U-test. Median.

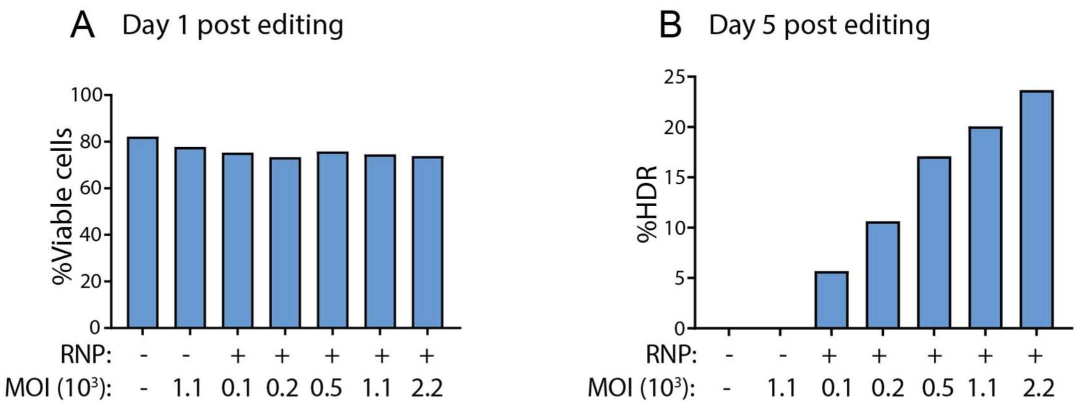

Figure S5. Optimization of AAV.FOXP3. cDNA MOI for achieving optimal cell viability and targeting efficiency. (A) Bar graphs presenting proportion of viable cells one day post-editing with T3 RNP and AAV.FOXP3.cDNA vector at the indicated MOIs. **(B)** HDR editing frequency determined by ddPCR of gDNA collected 5 days post-editing.

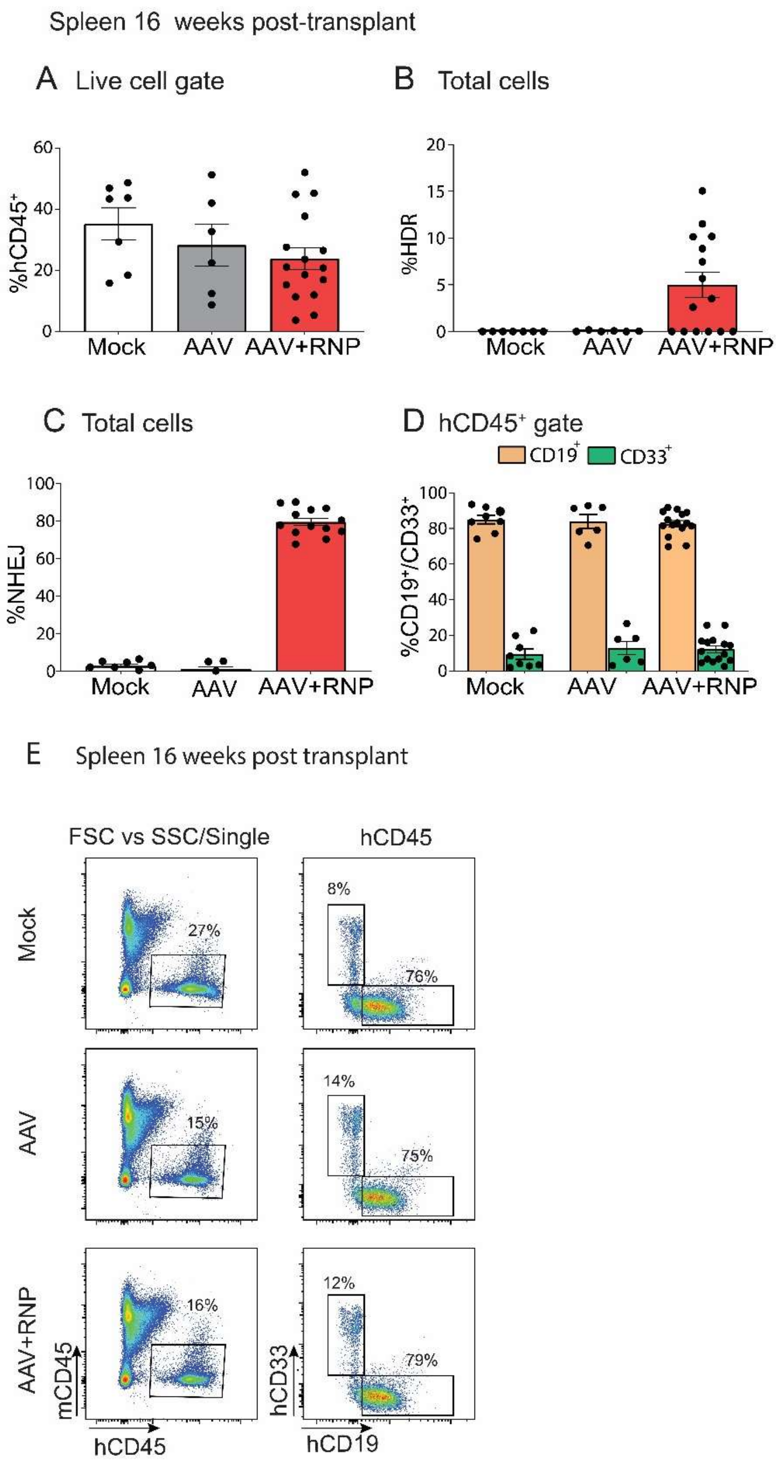
Spleen 16 weeks post-transplant
A Live cell gate
B Total cells
%hCD45+
%HDR
Mock
AAV
AAV+RNP
C Total cells
D hCD45+ gate
CD19+
CD33+
%NHEJ
%CD19+/CD33+
E Spleen 16 weeks post transplant
FSC vs SSC/Single
hCD45
Mock
AAV
AAV+RNP
27%
15%
16%
8%
76%
14%
75%
12%
79%
mCD45
hCD45
hCD33
hCD19

Figure S6. Long term engraftment of cDNA-edited HSPCs in the spleens of NBSGW mice. (A) Bar graphs depicting percent engraftment of human CD45+ cells in the spleens of NBSGW mice 16 weeks post transplantation. **(B-C)** Bars graphs showing percentage of bone marrow cells containing alleles with HDR- **(B)** and NHEJ-edits **(C)** as determined by ddPCR of splenic gDNA. **(D)** Bar graphs depict distribution of CD19+ B cells and CD33+ cells within the human $CD45^+$ splenic fraction. **(E)** Representative flow cytometry plots of splenocytes from NBSGW mice transplanted with $CD34^+$ cells receiving the indicated edits. All data are presented as mean +/- SEM.

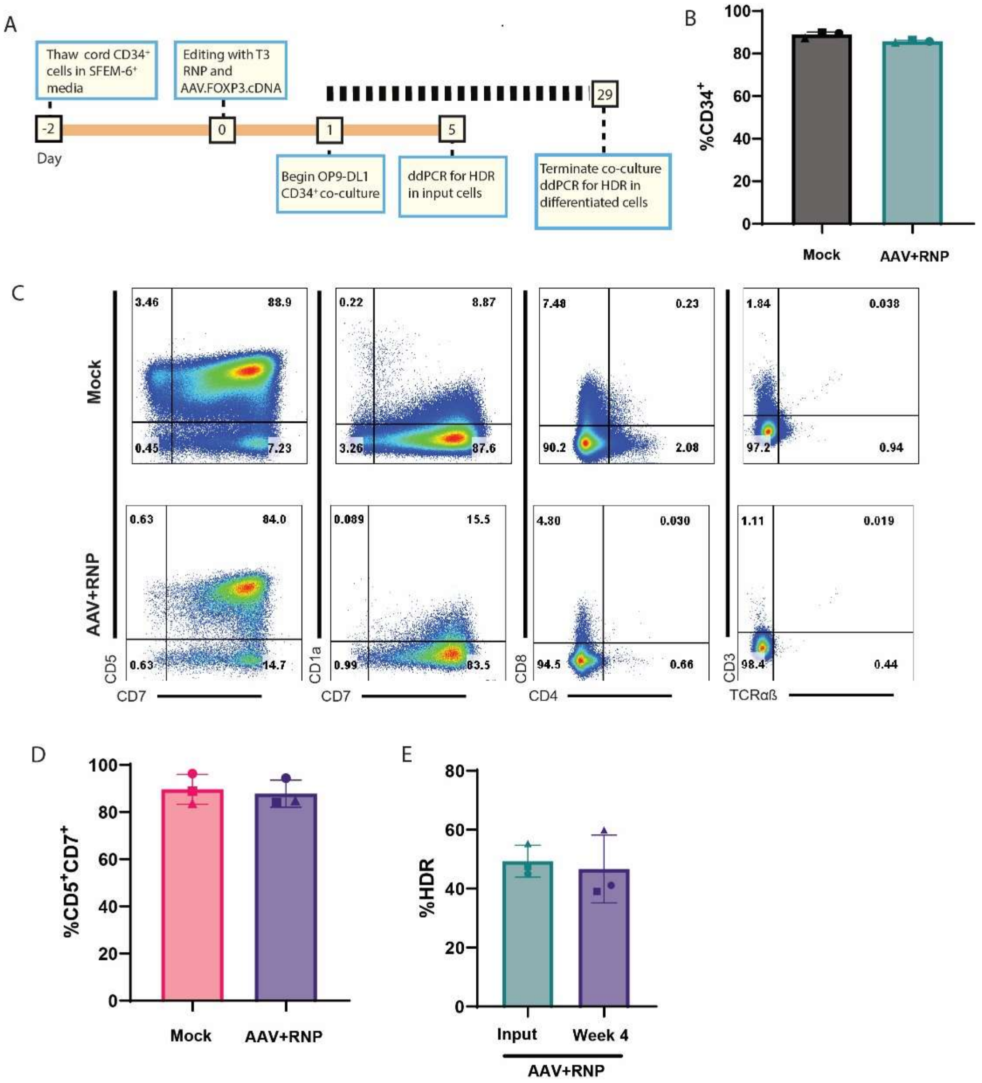

Figure S7. OP9-DL1 differentiation of AAV.FOXP3.cDNA edited CB-CD34$^+$. **(A)** Timeline of CB CD34$^+$ HSPC differentiation towards T lymphocyte lineage in OP9-DL1 in vitro differentiation system. **(B)** Proportion of HSPCs (CD34$^+$) at the initiation of OP9-DL1 differentiation. **(C)** Phenotype of differentiated mock and edited CB-CD34$^+$ cells after 28 days of differentiation in the ATO system. CD5 vs. CD7, CD1a vs. CD7, CD4 vs. CD8, and CD3 vs. TCRαß plots are gated by FSC vs SSC, SSC-A vs. SSC-W, hCD45+ CD34- and CD14-CD56-CD19- to exclude monocytes, NK cells, and B cells. **(D)** Proportion of pre T-1 cells (CD5+CD7+) at the termination of OP9-DL1 differentiation (gated on hCD45+CD34- and CD14-CD56-CD19-). **(E)** Proportion of HDR-edited cells at the initiation and termination of OP9-DL1 differentiation quantified by ddPCR Data represents three CD34$^+$ donors in two independent studies. Bar graphs represent mean +/- SEM.

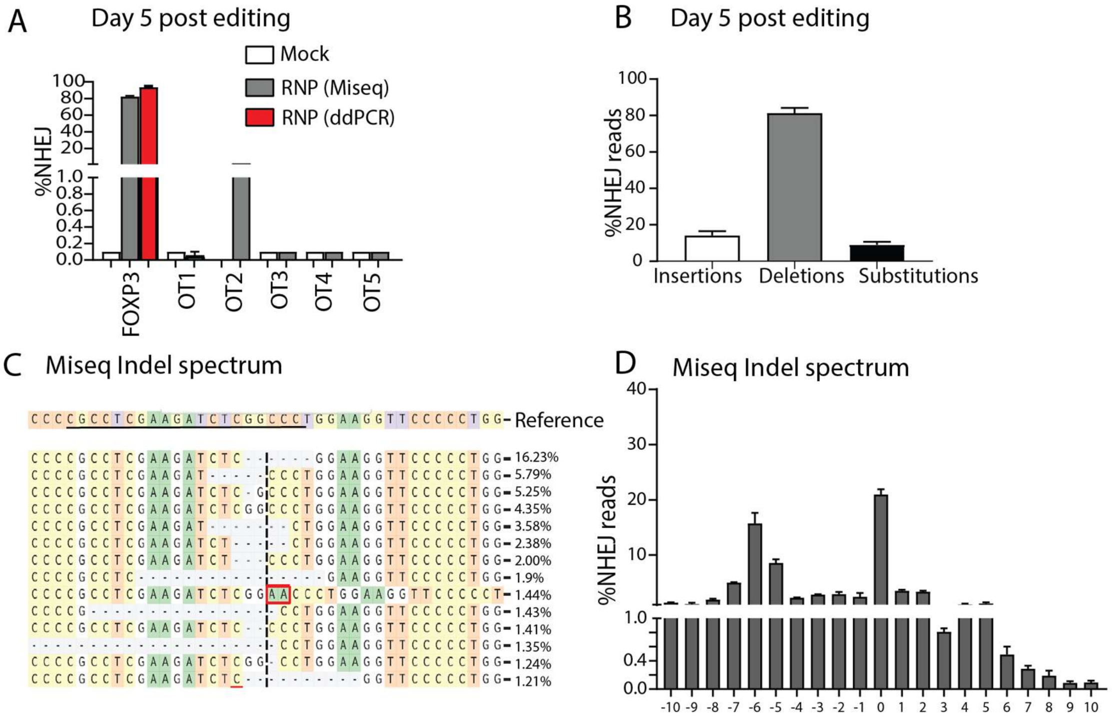

Figure S8. Analysis of on and off-target cleavage of sgRNA T3 in HSPCs using Miseq. (A) Graphs showing on and off target cleavage at *FOXP3* and the top 5 top off target sites for sgRNA T3. NHEJ rates determined by ddPCR at *FOXP3* are shown for comparison. N=3 independent donors for FOXP3 and N=2 for OT1 through 5. Genomic DNA collected 5 days post nucleofection was used for the on and off target analysis. **(B)** Graphs showing distribution of indels at *FOXP3* locus using Miseq platform. N=3 independent $CD34^+$ donors. **(C)** Summary of frequently observed indels in $CD34^+$ cells nucleofected by T3 RNP as determined by Miseq. The sequence of T3 sgRNA is underlined. **(D)** Bar graphs showing frequencies of indels in RNP nucleofected $CD34^+$ cells. N=3 independent $CD34^+$ donors.

Table S1: Summary of $CD34^{+}$ HSPCs editing protocols

Parameter	Protocol A	Protocol B
Culture media	SCGM with TPO, SCF, IL-6, Flt-3L (100ng/ml)	SFEMII with TPO, SCF, IL-6, Flt-3L (100ng/ml), SR1(1uM), UM171(35nM)
Pre-stimulation time	48 hr	48 hr
Cell density at the time of plating	1 x 10^6 cells/ml	2.5 x 10^5 cells/ml
Method of transfection	Electroporation or nucleofection	Nucleofection
Time of AAV addition	Immediately post electroporation	Immediately post electroporation
Plating density of cells post transfection	0.8 ×10^6 cells/ml	1×10^6 cells/ml

Table S2: Off target sites predicted for sgRNA T3 using CCtop

Coordinates	Target sequence	PAM	Region	Gene name	Distance from nearest gene
chr6:168307902-168307924	CACCGTGG[AGATCTCGGCCC]	GGG	Exon	DACT2	0
chr9:133485387-133485409	CCCCTCGG[AGACCTCGGCCC]	TGG	-	SLC2A6	6250
chr14:37584848-37584870	CGCGGCGG[AGCTCTCGGCCC]	GGG	-	FOXA1	5114
chr1:26022747-26022769	TGCCTCCC[AGACCTCGGCCC]	GGG	Exon	EXTL1	0
chr4:2439391-2439413	GACCTCGA[AGATCTGGGCCC]	AGG	Intron	CFAP99	1227

Table S3 – Antibodies

**Reagent or resource**	**Source**	**Identifier**
Human CD45 eFluor450, clone HI30	ThermoFisher Scientific	48045941
Mouse CD45 APC, clone 30-F11	ThermoFisher Scientific	17-0451-82
Human CD33 PE, clone WM53	BD Biosciences	555450
Human CD19 PE Cy7, clone HIB19	ThermoFisher Scientific	25019942
Human CD34 APCCy7, clone 581	BioLegend	343514
Human CD38 PerCPCy5.5, clone HIT2	BD Biosciences	551400
Human CD90 APC, clone 5E10	BD Biosciences	561971
Human CD133 PE, clone AC133	Miltenyi Biotec	130-113-108
Human CD4 eFluor450, clone OKT4	ThermoFisher Scientific	48004842
Human CD8 PerCPCy5.5, clone RPA-T8	BD Biosciences	560662
Human CD25 PECy7, clone 2A3	BD-Biosciences	335789
Human CD127 BV510, HIL-7R-M21	BD-Biosciences	563086
Human FoxP3 PE, clone 254D	Biolegend	320208
Human CD4 Alexa700, clone OKT4	ThermoFisher Scientific	50-168-56
Human CD3 BV786, clone SK7	BioLegend	344842
Human CD4 BV605, clone RPA-T4	BioLegend	300556

Human CD8 Alexa700, clone SK1	BioLegend	344724
Human CD19 PECy7, clone HIB19	BioLegend	302215
Human CD56 PECy7, clone HCD56	BioLegend	318318
Human CD14 PECy7, clone 61D3	ThermoFisher Scientific	56-0149-42
Human TCRab, PE, clone IP26	BioLegend	306708
Human CD5 APC, clone L17F12	BioLegend	364016
Human CD7 FITC, clone CD7-6B7	BioLegend	343104
Human CD1a PerCPCy5.5, clone HI149	BioLegend	300130
Human CD8 Alexa700, clone RPA-T8	BD Biosciences	557945

Table S4 - Experimental model/strains

**Resource or reagent**	**Source**	**Identifier**
NBSGW<br><br>NOD.Cg-KitW-41J Tyr +<br><br>Prkdcscid Il2rgtm1Wjl/ThomJ	Jackson Laboratory	Stock 026622

Table S5– Biological samples - cell lines

**Resource or reagent**	**Source**	**Identifier**

OP9-DL1 cells	Dr. Irwin Bernstein (University of Washington)	N/A
MS5-DLL4 cells	Dr. Gay Crooks (University of California Los Angeles)	N/A

Table S6 – Biological samples – primary cells

**Resource or reagent**	**Source**	**Identifier**
Mobilized peripheral blood $CD34^+$ HSPCs	Fred Hutch Co-operative Center for Excellence in Hematology	N/A
Cord blood $CD34^+$ HSPCs	STEMCELL Technologies	70008.5
Cord blood $CD34^+$ HSPCs from IPEX patient	Seattle Children's Hospital	N/A
Peripheral blood mononuclear cells	Fred Hutch Co-operative Center for Excellence in Hematology	N/A
Peripheral blood mononuclear cells from IPEX patient	Seattle Children's Hospital	N/A

Table S7– Oligonucleotides and probes

**Reagent or resource**	**Source**	**Identifier**

NHEJ forward CACGTGTGACTCCTTTCCC	IDT	N/A
NHEJ reverse CACGTGTGACTCCTTTCCC	IDT	N/A
T3_NHEJ probe AGCTGGGCGAGGCTCCT	IDT	N/A
T9_NHEJ probe AGCTGGGCGAGGCTCCT	IDT	N/A
NHEJ Control forward CGACACTTCACCCCTTTTCT	IDT	N/A
NHEJ Control reverse CTCCCCAATGTGCCTATGAG	IDT	N/A
NHEJ Control Probe GTGGCGGTGACTGGGATGGC	IDT	N/A
HDR forward GAGCAAAGACCCCAACGAGA	IDT	N/A
HDR reverse GACTTGGGGGTTCTGTGAAG	IDT	N/A
HDR GFP probe GCATGGACGAGCTGTACAAG	IDT	N/A
HDR cDNA forward GAACCCTGAACGAGATCTACC	IDT	N/A
HDR cDNA reverse CTGTGGTTCAGCCTGACTCG	IDT	N/A
HDR cDNA probe TCCTGGTTAGTTCTTGCCAC	IDT	N/A
HDR control *ActB* forward ACTCTGCAGGTTCTATTTGC	IDT	N/A
HDR control *ActB* reverse AATGATCTGAGGAGGGAAGG	IDT	N/A
HDR probe *ActB* ATCAAGGTGGGTGTCTTTCC	IDT	N/A
HDR control *CCR5* forward AAAGATTTGCAGAGAGATGAGT	IDT	N/A
HDR control *CCR5* reverse GCCAAGCAATGAAGTTTTGT	IDT	N/A
HDR probe *CCR5* CCTGGGCAACATAGTGTGATC	IDT	N/A

FOXP3co transcript forward<br><br>CAGCTGGTCCTGGAAAAAGA	IDT	N/A
FOXP3co transcript reverse<br><br>AACATCCGGGTAAACCAGTG	IDT	N/A
FOXP3co transcript probe TCCTGCACAACATGGACTAC	IDT	N/A
TaqMan Gene Expression Assay (FOXP3 endogenous)	ThermoFisher Scientific	Hs01085831_g1
TaqMan Gene Expression Assay (HPRT)	ThermoFisher Scientific	Hs99999909_m1

Table S8 – Reagent list

**Reagent**	**Source**	**Identifier**
Recombinant human TPO	Peprotech	300-18
Recombinant human SCF	Peprotech	300-07
Recombinant Human Flt3-Ligand	Peprotech	300-19
Recombinant human IL-6	Peprotech	200-06D
Recombinant human IL-7	Peprotech	200-07
StemRegenin1	STEMCELL Technologies	72344
UM171	ApexBio	NC0806207
HEPES	Gibco	15630080
Glutamax	Gibco	35050061
B-mercaptoethanol	Sigma-Aldrich	M6250
DNeasy Blood and tissue Kit	Qiagen	69506
CellGenix® GMP SCGM	CellGenix	20802-0500

Stem Cell Growth Medium		
Stemspan SFEMII	STEMCELL Technologies	09655
RPMI-1640	Gibco	11875093
Alpha MEM with Nucleosides	STEMCELL Technologies	36450
DMEM	Gibco	11965092
Fetal bovine serum	Omega Scientific	FB-11
Busulfan (Busulfan injections)	Otsuka America Pharmaceutical	59148-070-90
CRISPRevolution sgRNA EZ Kit	Synthego	N/A
SpyFi Cas9 Nuclease	Aldevron	N/A
Agencourt AMPure XP	Beckman Coulter	A63881
MiSeq 500 CycleV2 kit	Illumina	MS-102-2003
PrimeSTAR GXL DNA polymerase	Clontech	R050B
ddPCR Supermix for Probes without UTP	Bio-Rad	1863025
Droplet Generation Oil for Probes	Bio-Rad	1863005
Droplet reader oil	Bio-Rad	1863004
RNeasy mini kit	Qiagen	74106
Maxima First Strand cDNA Synthesis Kit for qRT-PCR	Thermofisher Scientific	FERK1672
CD34 MicroBead Kit, human	Miltenyi Biotech	130-046-702
EasySep™ Human $CD4^+$ T cell enrichment kit	STEMCELL Technologies	19052

EasySep™ Human CD4+CD127lowCD25⁺ Regulatory T Cell Isolation Kit	STEMCELL Technologies	18063
True-Nuclear Transcription Factor buffer set	BioLegend	424401
Dynabeads Human T-Expander CD3/CD28	Gibco	11141D

Table S9 – Software and algorithm

**Reagent or resource**	**Source**	**Identifier**
QuantaSoft Analysis Software	Bio-Rad	1864003
Prism 7.03 or later software package	Graph Pad Software	N/A
CRISPResso	[51]	N/A

Table S10 – Other materials

**Reagent or resource**	**Source**	**Identifier**
QX200 Droplet reader	Bio-Rad	1864001
QX200 Droplet Generator	Bio-Rad	N/A
LSRII	BD Biosciences	N/A
BD FACSAria II cell sorter	BD Biosciences	N/A
