# Supplementary material for: Efficient and sustained FOXP3 locus editing in hematopoietic stem cells as a therapeutic approach for IPEX syndrome
Source: Mol Ther Methods Clin Dev. 2023 Dec 26;32(1):101183. doi: 10.1016/j.omtm.2023.101183 (PMC10818254; doi:10.1016/j.omtm.2023.101183)
Supplement: Document S2. Article plus supplemental information [file mmc3.pdf]

Molecular Therapy
Methods & Clinical Development
Original Article

Efficient and sustained *FOXP3* locus editing in hematopoietic stem cells as a therapeutic approach for IPEX syndrome

Swati Singh,[1,4] Cole M. Pugliano,[1,4] Yuchi Honaker,[1] Aidan Laird,[1] M. Quinn DeGottardi,[1] Ezra Lopez,[1] Stefan Lachkar,[1] Claire Stoffers,[1] Karen Sommer,[1] Iram F. Khan,[1] and David J. Rawlings[1,2,3]

[1]Center for Immunity and Immunotherapies and the Program for Cell and Gene Therapy, Seattle Children's Research Institute, Seattle, WA 98101, USA; [2]Department of Pediatrics, University of Washington, Seattle, WA 98101, USA; [3]Department of Immunology, University of Washington, Seattle, WA 98101, USA

Immune dysregulation, polyendocrinopathy, enteropathy, X-linked (IPEX) syndrome is a monogenic disorder caused by mutations in the *FOXP3* gene, required for generation of regulatory T (T_{reg}) cells. Loss of T_{reg} cells leads to immune dysregulation characterized by multi-organ autoimmunity and early mortality. Hematopoietic stem cell (HSC) transplantation can be curative, but success is limited by autoimmune complications, donor availability and/or graft-vs.-host disease. Correction of FOXP3 in autologous HSC utilizing a homology-directed repair (HDR)-based platform may provide a safer alternative therapy. Here, we demonstrate efficient editing of *FOXP3* utilizing co-delivery of Cas9 ribonucleoprotein complexes and adeno-associated viral vectors to achieve HDR rates of >40% *in vitro* using mobilized $CD34^+$ cells from multiple donors. Using this approach to deliver either a GFP or a FOXP3 cDNA donor cassette, we demonstrate sustained bone marrow engraftment of approximately 10% of HDR-edited cells in immune-deficient recipient mice at 16 weeks post-transplant. Further, we show targeted integration of FOXP3 cDNA in $CD34^+$ cells from an IPEX patient and expression of the introduced FOXP3 transcript in gene-edited primary T cells from both healthy individuals and IPEX patients. Our combined findings suggest that refinement of this approach is likely to provide future clinical benefit in IPEX.

INTRODUCTION

Immune dysregulation, polyendocrinopathy, enteropathy, X-linked (IPEX) syndrome is a rare monogenic primary immunodeficiency, characterized by the loss of functional regulatory T (T_{reg}) cells crucial for controlling immune responses against self and foreign antigens. The syndrome was first described in 1982[1] in a family with several affected males and the responsible gene, Forkhead box P3 (FOXP3), was identified several years later.[2,3] FOXP3 is the lineage-defining transcription factor of thymically derived T_{reg} cells and is essential for both T_{reg} cell development and function. Absent or dysfunctional T_{reg} cell in IPEX patients leads to the failure to maintain peripheral immune tolerance, resulting in the early onset of multi-system autoimmunity with features including, most commonly, severe inflammatory bowel disease, type 1 diabetes mellitus, thyroid disease, and eczema. Supportive immunosuppressive therapies can modulate disease, but are not curative and are associated with multiple complications. Alternatively, allogeneic hematopoietic stem cell transplantation (HSCT) represents a potentially curative approach that can eliminate autoimmune manifestations.[4] However, while HSCT can be highly beneficial for IPEX, limitations in donor matching and transplant complications in the setting of severe immunologic dysregulation make implementation of this approach highly challenging.[4,5]

Prior work has demonstrated the efficacy of lentiviruses (LVs) expressing FOXP3 from a constitutively active EF1α promoter to establish a T_{reg} cell phenotype in IPEX $CD4^+$ T cells.[6] While potentially advantageous for applications where adoptive transfer of T_{reg} cells might be beneficial,[7,8] adoptively transferred LV-treated T cells are unlikely to provide a long-term cure for IPEX due to the inability to persist *in vivo* over long periods of time. Gene therapy of murine HSCs using LV with the endogenous FOXP3 promoter driving FOXP3 cDNA expression have also shown promise by rescuing the autoimmune phenotype in scurfy mice, the murine equivalent of IPEX.[9] This approach, however, will require LVs that can precisely replicate the complex endogenous control elements within the *FOXP3* locus capable of initiating and sustaining endogenous levels of FOXP3 expression, while limiting both vector silencing and genotoxicity risk due to the random nature of LV integration.

CRISPR-Cas9-based gene editing[10,11] of IPEX patient $CD34^+$ hematopoietic stem and progenitor cells (HSPCs) offers an alternative therapeutic option. Homology-directed repair (HDR)-based editing enables insertion of functional FOXP3 cDNA sequence into the endogenous locus while preserving adjacent sequence elements

Received 14 June 2022; accepted 20 December 2023;
https://doi.org/10.1016/j.omtm.2023.101183.

[4]These authors contributed equally

Correspondence: David J. Rawlings, Center for Immunity and Immunotherapies, Seattle Children's Research Institute, 1900 Ninth Avenue, Seattle, WA 98101, USA.
E-mail: drawling@uw.edu

This is an open access article under the CC BY license (http://creativecommons.org/licenses/by/4.0/).

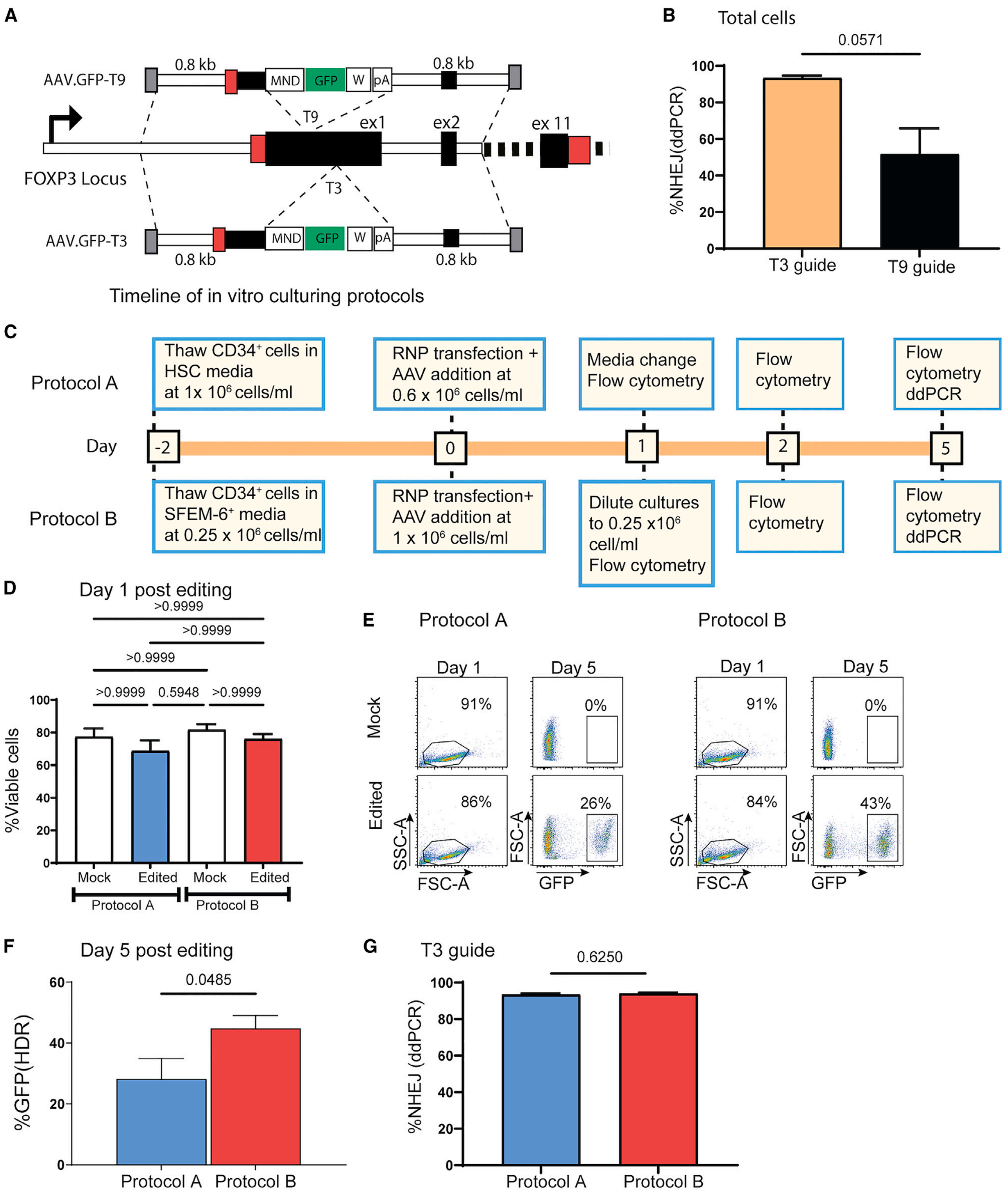

Figure 1. Targeted gene integration in primary human CD34+ HSPCs treated with CRISPR-Cas RNPs and AAV6 vectors

(A) Schematic of the *FOXP3* genomic locus and the AAV6 targeting vectors specific for sgRNAs T9 or T3 designed to insert the MND promoter-driven GFP expression cassette into exon 1 of *FOXP3*. Black and red rectangles represent exons and UTR elements, respectively; the location of homology arms with respect to the *FOXP3* locus is

(legend continued on next page)

required for expression and ensuring appropriate copy number. Gene editing for IPEX is particularly appealing since locus- and lineage-specific regulation of *FOXP3* is critical for stable FOXP3 expression.[12,13] Thus, preserving the natural genomic landscape at the locus in HDR-edited HSPCs is paramount to enable appropriate differentiation and acquisition of T_{reg} cell fate.

While *in vitro* editing has been reported at high rates in HSPCs sourced from umbilical cord blood (CB) or isolated from peripheral blood of granulocyte colony stimulating factor (G-CSF) mobilized adults (mPB), engraftment of HDR-edited HSPCs in immune deficient animals remains challenging due to the difficulty in targeting long-term repopulating HSCs (LT-HSCs); a feature that is particularly evident when using mPB HSPCs.[14] Importantly, analysis of in IPEX patients following HSCT and female carriers of IPEX suggests that even low levels of functional FOXP3 expression may be sufficient to alleviate disease symptoms due to the high selective advantage for T_{reg} cells.[15–18] Thus, engraftment of even a limited proportion of successfully HDR-edited HSC is predicted to provide clinical benefit in the setting of this profound immune disorder.

We have recently demonstrated highly efficient methods to edit the *FOXP3* locus in primary T cells enabling conversion to thymus T_{reg} (tT_{reg})-like cells capable of mediating immunosuppression in the setting of autoimmunity.[19] Another group has published HDR-based editing of *FOXP3*, although the majority of experiments utilize CB-HSPCs rather than more clinically relevant mPB $CD34^+$ HSPCs.[20] Here, we present a targeted gene editing strategy to incorporate the codon diverged coding region for *FOXP3* gene in mPB HSPCs and test their ability to engraft in immune deficient mice. Further, we demonstrate efficient HDR-editing of IPEX patient $CD34^+$ cells and verify the expression of codon-diverged FOXP3 transcripts in HDR-edited primary $CD4^+$ T cells derived from both healthy controls and IPEX patients.

RESULTS

Optimization of culture conditions for editing $CD34^+$ cells at the *FOXP3* locus

The *FOXP3* gene comprises 11 coding exons and mutations reported in IPEX patients have been identified throughout the entire gene.[21] To develop an editing strategy that can work as a universal cure for IPEX patients, we elected to insert a functional FOXP3 cDNA within the first coding exon, to enable correction of all IPEX patients except those bearing mutations within the promoter region.[22,23] This strategy was also utilized to disrupt the mutant FOXP3 allele and eliminate the potential for expression of a dominant negative mutant protein. We used CRISPR single-guide RNA (sgRNA) T9,[19] and a second sgRNA T3, to target the first coding exon of the *FOXP3* gene (Figure 1A). For comparison, the sgRNA utilized by Goodwin et al.[20] binds at the intersection of 5′ UTR/first coding exon and 47 and 125 nucleotides away from sgRNA T9 and T3, respectively. The sgRNAs were complexed with SpyFi Cas9[24] to form ribonucleic proteins (hereafter referred to as RNPs) then electroporated into HSPCs using the Neon electroporation system. Analysis of gDNA by droplet digital PCR (ddPCR) revealed that T3 RNP induced insertions/deletions (indels) in approximately 93%, while T9 targeted approximately 52% of *FOXP3* alleles, respectively (Figure 1B), indicating superior on-target cleavage with T3 RNP in $CD34^+$ cells.

To orchestrate and efficiently track HDR, recombinant adeno-associated virus (rAAV) donor templates were first designed to insert an expression cassette containing a constitutive myeloproliferative sarcoma virus enhancer, negative control region deleted, dl587rev primer-binding site substituted (MND) promoter driven GFP cDNA sequence followed by shortened woodchuck hepatitis post-transcriptional regulatory element (WPRE3) and Simian virus 40 polyadenylation (SV40 polyA) sequences. Flanking the expression cassette, 0.8-kb homology arms centered specifically on the respective sgRNA were designed such that a minimum deletion was created after targeted integration (Figure 1A). Editing with either sgRNA and sgRNA-specific AAV HDR donor combination led to robust HDR rates. As anticipated, we observed 4-fold higher targeted modification using T3- (GFP-T3) or T9-specific (GFP-T9) AAV donors (Figures S1A and S1C) when compared with a common AAV.GFP donor that functioned with either sgRNA, findings that also demonstrated no significant negative impacts on cell viability (Figures S1B and S1D). For the GFP-T3 vector, no enhancement in HDR was observed as vector dose was increased (multiplicity of infections [MOI] from 250 to 1,000), while a dose-dependent increase was observed with the GFP-T9 AAV donor (Figures S1A and S1C). Since absolute HDR rates were considerably higher at 51% for the T3 RNP and GFP-T3 AAV (vs. 8% using T9 RNP), we elected to utilize the T3 RNP for all subsequent experiments.

HSCs are largely quiescent, thereby limiting DNA damage due to replication and mitosis. Because HDR pathways are active primarily during G2 and S phases, HSCs are more likely to undergo non-homologous end-joining (NHEJ) upon introduction of a double-strand

depicted by dashed lines. pA, SV40 polyadenylation sequence; W, WPRE3 element. (B) Average allelic disruption (% NHEJ) rates observed upon transfection of adult mobilized human $CD34^+$ HSPCs with T3 (n = 3 male donors, 3 independent studies) or T9 (n = 3 male donors, 3 independent studies) RNPs quantified via ddPCR. Significance determined by Mann-Whitney *U* test. (C) Timeline of procedures for *in vitro* culturing and editing of adult mobilized $CD34^+$ HSPCs using protocol A (top) and B (bottom). (D) Viability measured by flow cytometry forward and side scatter 1 day after editing with T3 RNP and rAAV6 targeting vector employing either protocol A (n = 6 male donors, 12 independent studies) or protocol B (n = 5 male donors, 1 female donor, 9 independent studies). Significance determined by Kruskal-Wallis test. (E) Representative flow cytometry plots depicting cell viabilities on day 1 and $\%GFP^+$ cells on day 5 in HSPCs edited with protocol A (left) or B (right). (F) Targeted integration rates measured by GFP-high 5 days after editing with T3 RNP and rAAV6 targeting vector cultured with protocol A (n = 4 male donors, 4 independent studies) or protocol B (n = 5 male donors, 1 female donor, 8 independent studies). Significance determined by Mann-Whitney *U* test. (G) NHEJ 5 days after editing in cells cultured with protocol A or B and transfected with T3 RNP (n = 3 male donors in 4 independent studies). Wilcoxon matched-pairs signed ranked test. Bar graphs represent mean ± SEM.

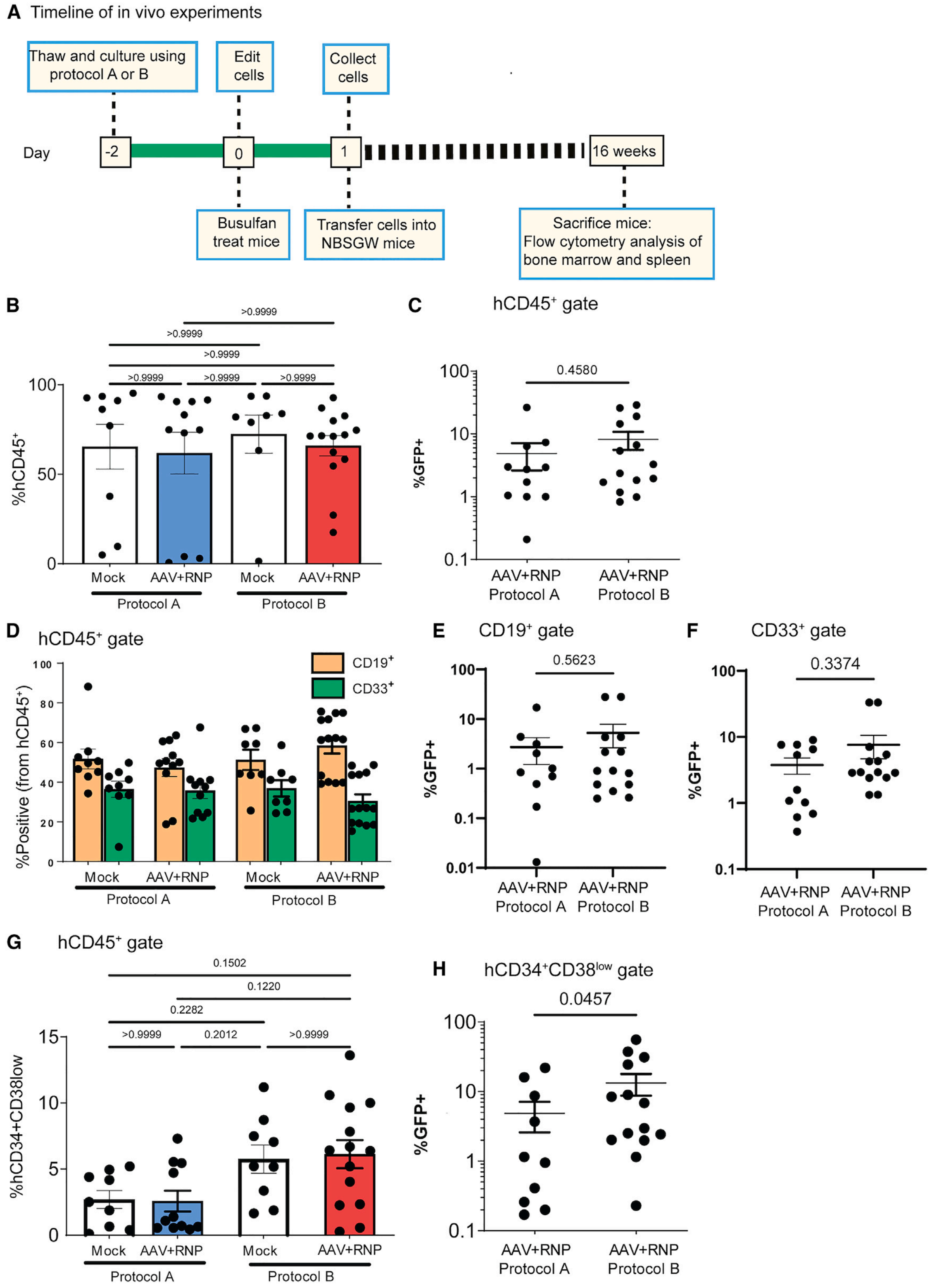

(legend on next page)

break (DSB).[25,26] To facilitate entry of HSC into cell cycle and repair of DSBs by HDR utilizing the AAV donor, we first pre-stimulated CD34$^+$ cells in culture media with cytokines. Of note, low-density cultures have also been reported to promote HSC expansion and cycling.[27] Additionally, SR1 and UM171, compounds reported to support HSC expansion[28] and self-renewal,[29] were included in protocol B. To explore the impact of alternative culture densities and media on the indel and HDR frequencies, CD34$^+$ cells were plated in media using a higher cell density (protocol A) vs. lower density protocol (protocol B), as outlined in Figure 1C. Specific differences between the two protocols are summarized in Table S1. For protocol B, the nucleofection was performed using the optimized program CM-149 (Figure S2). Flow cytometry analysis 1 day after editing revealed slightly higher cell viabilities in both mock and edited cells for protocol B (mock 79%, edited 72%) vs. protocol A (mock 72%, edited 63%) (Figure 1D). To assess potential differences in inducing indel and HDR frequencies, T3 RNPs and GFP-T3 AAV donor vectors were introduced into cells cultured using both protocols followed by flow cytometry to assess HDR rates based on % GFP$^+$ cells at day 5 after editing. HDR rates were also subsequently assessed via ddPCR. A significantly higher average GFP expression was observed in the CD34$^+$ cells cultured with protocol B averaging 44% compared with 28% for protocol A (Figures 1E and 1F), despite equivalent percent NHEJ rates (93%) using either protocol (Figure 1G).

Edited HSPCs engraft long-term in NBSGW mice and undergo multi-lineage differentiation

To determine whether editing protocols influenced the long-term repopulation potential of CD34$^+$ cells, mock or GFP-T3 AAV plus T3 RNP-edited CD34$^+$ cells were transplanted into busulfan-treated, 8- to 10-week-old NBSGW recipient mice (Figure 2A). Human cell chimerism was evaluated by sacrificing the mice at 12–16 weeks after transplant and analyzing bone marrow (BM) and spleens for engrafted CD45$^+$ human cells and GFP$^+$ edited cells. Similar percentages of human cells were present within the BM for recipients of cells cultured with either protocol (protocol A: mock, 65%; edited 62%; and protocol B: mock 72%; edited 58%) (Figures 2B and S3A). However, the average %GFP$^+$ cells observed within the BM were 1.6-fold higher when protocol B was used (average 8%; range, 0.2%–26.0%) compared with protocol A (average 5%; range, 1%–29%) (Figure 2C). The distribution of CD19$^+$ B cells and CD33$^+$ myeloid cells were similar across protocols and groups (Figure 2D). The %GFP$^+$ cells within both compartments was slightly higher with protocol B compared with protocol A and CD33$^+$ cells exhibited the highest %GFP$^+$ cells (Figures 2E and 2F).

The mean human CD45$^+$ cell engraftment within the spleen was also comparable between groups, ranging from 12% to 19%, with no significant differences between protocols (Figure S4A). Matching the trend observed in the BM, GFP$^+$ cells were higher for protocol B compared with protocol A edited cells, averaging 10% and 4%, respectively (Figure S4B). The proportion of CD33$^+$ cells were comparable across all groups. Interestingly, proportion of CD19$^+$ B cells in both mock and edited groups was higher using protocol B (Figure S4C) and significantly higher %GFP$^+$ cells were observed for protocol B, 5% compared with 2% for protocol A (Figure S4D).

A key obstacle for developing durable HDR-based gene editing therapies using HSPCs is the inherent quiescence of LT-HSCs that serves as a protective mechanism against endogenous stress.[30] To assess the impact of alternative culture conditions on HDR editing in HSCs, we analyzed human cells within the BM of NBSGW mice for expression of surface markers that define a more primitive, engraftment-enriched subset of HSPCs, defined by CD34$^+$CD38^{low} (Figure S3B). The proportion CD34$^+$CD38^{low} HSPCs was approximately 2-fold higher with use of protocol B (5%–6%), irrespective of editing (Figure 2G). Strikingly, analysis of GFP$^+$ cells within CD34$^+$CD38^{low} compartment revealed a 3-fold higher proportion of HDR-edited cells for protocol B, 13% compared with 5% for protocol A (Figure 2H). These findings demonstrated that use of UM171 and SR1, in association with the low-density culturing in protocol B, enabled superior long-term engraftment of HSPCs and increased HDR editing efficiency in the CD34$^+$CD38^{low} compartment and was selected for use in all ensuing studies.

Sustained engraftment and differentiation of FOXP3 cDNA-edited HPSC in NBSGW mice

We next modified our targeting AAV vector to enable insertion of a clinically relevant codon diverged FOXP3 cDNA at the endogenous start site of *FOXP3* (AAV.FOXP3cDNA) (Figure 3A). HDR rates observed upon editing mobilized CD34$^+$ cells using T3 RNP increased in proportion to the dose of the AAV.FOXP3.cDNA template (100–2,200 MOI) (Figure S5B). No significant impact on cell viability was observed even at the highest AAV MOI evaluated (Figure S5A). Using these reagents and a MOI of 2,000, we achieved efficient HDR (average 42%) with minimal impact to cell viability (Figures 3B and 3C).

Figure 2. Long-term hematopoietic reconstitution from HDR-edited HSPCs in the BM of transplanted NBSGW mice

(A) Procedure for *in vivo* engraftment of edited HSPCs in NBSGW mice. (B) Engraftment (%hCD45$^+$) in the BM of NBSGW recipient mice 12–16 weeks after transplant of protocol A mock treated (n = 9, 2 male donors), protocol A edited (n = 11, 2 male donors), protocol B mock treated (n = 8, 1 male donor, 1 female donor), protocol B edited (n = 14, 1 male donor, 1 female donor) from two independent studies. Significance determined by Kruskal-Wallis test. (C) Percentage of hCD45$^+$ HDR-edited (GFP+) cells in the BM of NBSGW recipients from (B) Significance determined by Mann-Whitney *U* test. (D) Proportion of B cells (CD19$^+$) and myeloid cells (CD33$^+$) in the BM of NBSGW recipients from (B). (E) Percentage of HDR-edited (GFP$^+$) CD19$^+$ cells from (D) Significance determined by Mann-Whitney *U* test. (F) Percentage of HDR-edited (GFP$^+$) CD33$^+$ cells from (D) Significance determined by Mann-Whitney *U* test. (G) Proportion of HSPCs (CD34$^+$CD38low) in the BM of NBSGW recipients from (B) Significance determined by Kruskal-Wallis test. (H) Proportion of HDR-edited (GFP$^+$) HSPCs (CD34$^+$CD38low) from (G). Significance determined by Mann-Whitney *U* test. Bar graphs represent mean ± SEM.

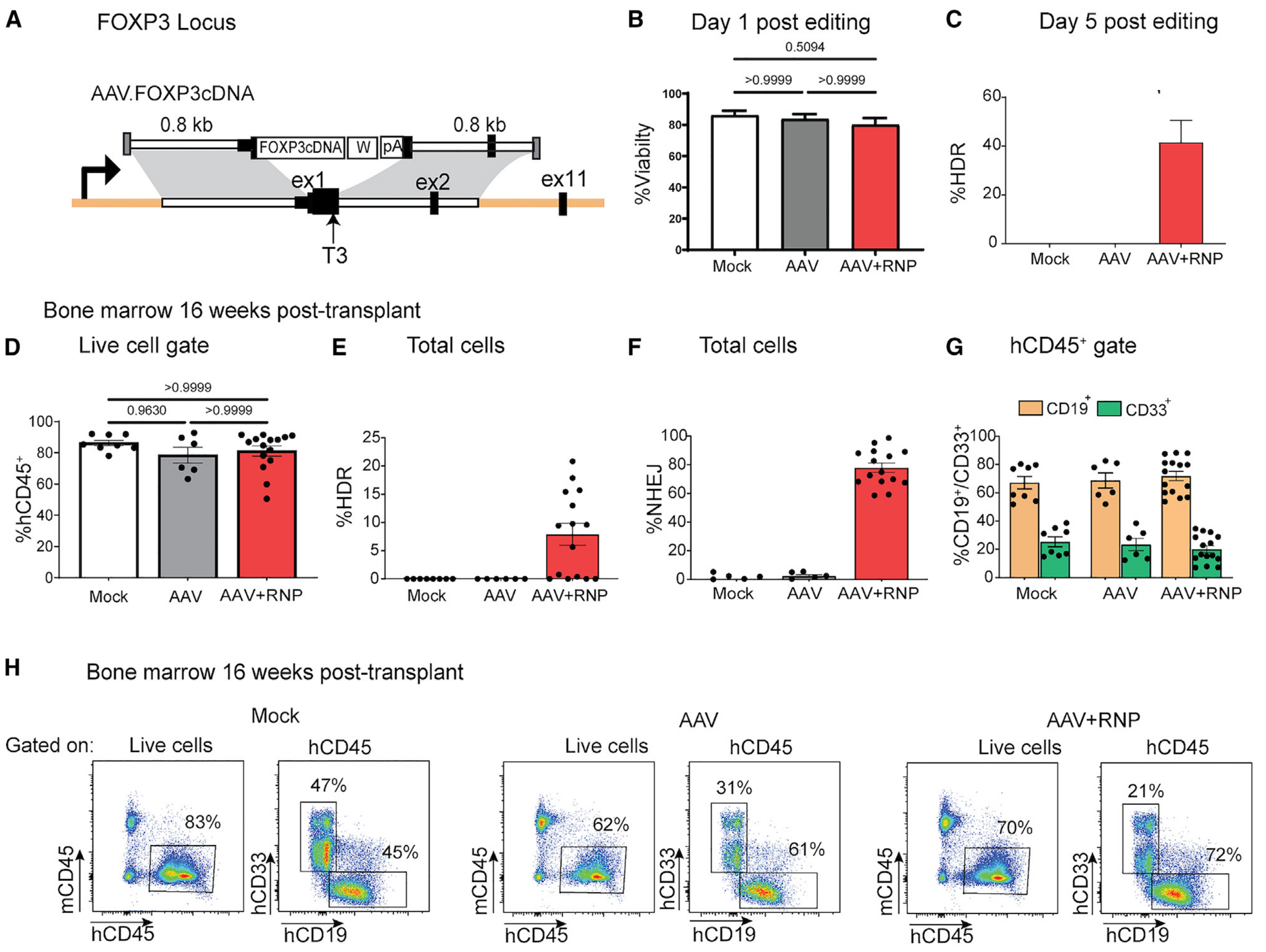

Figure 3. Long-term engraftment and differentiation of cDNA-edited cells in the BM of NBSGW mice after transplantation of HDR-edited HSPCs

(A) Schematic of the *FOXP3* genomic locus and the rAAV6 targeting vector utilized to insert codon optimized FOXP3 cDNA at the endogenous start site of the *FOXP3* gene. FOXP3 cDNA, codon optimized cDNA sequence for FOXP3; pA, SV40 polyadenylation sequence; W, WPRE3 element. (B) Viability of HSPCs 1 day after editing with T3 RNP + AAV.FOXP3.cDNA using protocol B (n = 3 male donors, 4 independent studies). Significance determined by Kruskal-Wallis test. (C) HDR frequency determined by ddPCR of gDNA extracted from cells 5 days after editing with T3 RNP + AAV.FOXP3.cDNA (n = 3 male donors, 3 independent studies). (D) Engraftment (%hCD45$^+$) in the BM of NBSGW recipient mice 16 weeks after transplant of mock (n = 8, 1 male donor, 1 female donor), AAV (n = 6, 1 male donor, 1 female donor), or AAV+RNP (n = 15, 1 male donor, 1 female donor) HSPCs cultured with protocol B from two independent studies. Significance determined by Kruskal-Wallis test. (E) HDR frequency determined by ddPCR of gDNA extracted from the BM of NBSGW recipient mice from (D). (F) NHEJ frequency determined by ddPCR of gDNA extracted from the BM of NBSGW recipient mice from (D). (G) Proportion of B cells (CD19$^+$) and myeloid cells (CD33$^+$) in the BM of NBSGW recipient mice from (D). (H) Representative flow cytometry data from BM of NBSGW recipient mice from (D). Bar graphs represent mean ± SEM.

Mock, AAV.FOXP3.cDNA only, and AAV.FOXP3.cDNA plus RNP-treated CD34$^+$ cells were transplanted into cohorts of NBSGW mice. Investigation of BM of mice sacrificed 16 weeks later revealed similar levels of average human cell chimerism—87% in mock, 79% in AAV only, and 83% in AAV plus RNP-treated transplanted mice (Figure 3D). Within the spleen, engraftment rates were 35%, 28%, and 24% for these groups, respectively (Figure S6A). Importantly, sustained HDR editing rates were observed within human cells recovered from BM (mean 8%; range, 0%–21%) and spleen (mean 5%; range, 0%–15%) (Figures 3E and S6B). We also determined the NHEJ rates in recipients of AAV plus RNP-treated cells, revealing high rates in both BM (78%) and spleen (79%) (Figures 3F and S6C). Minimal differences were observed in the percentage of CD19$^+$ or CD33$^+$ cells across groups, demonstrating that HDR editing with AAV.FOXP3.cDNA did not compromise *in vivo* differentiation potential (Figures 3G, S6D, and S6E).

cDNA-edited HSPCs differentiate into T lymphocytes that maintain targeted integration

Next, we sought to determine whether cDNA edited CD34$^+$ HSPCs retain the potential to develop into naive CD4 T cells, progenitors capable of differentiation into the T_{reg} cell lineage *in vivo* in response to critical differentiation cues. As the adult NBSGW model used in Figures 2 and 3 does not support robust T cell

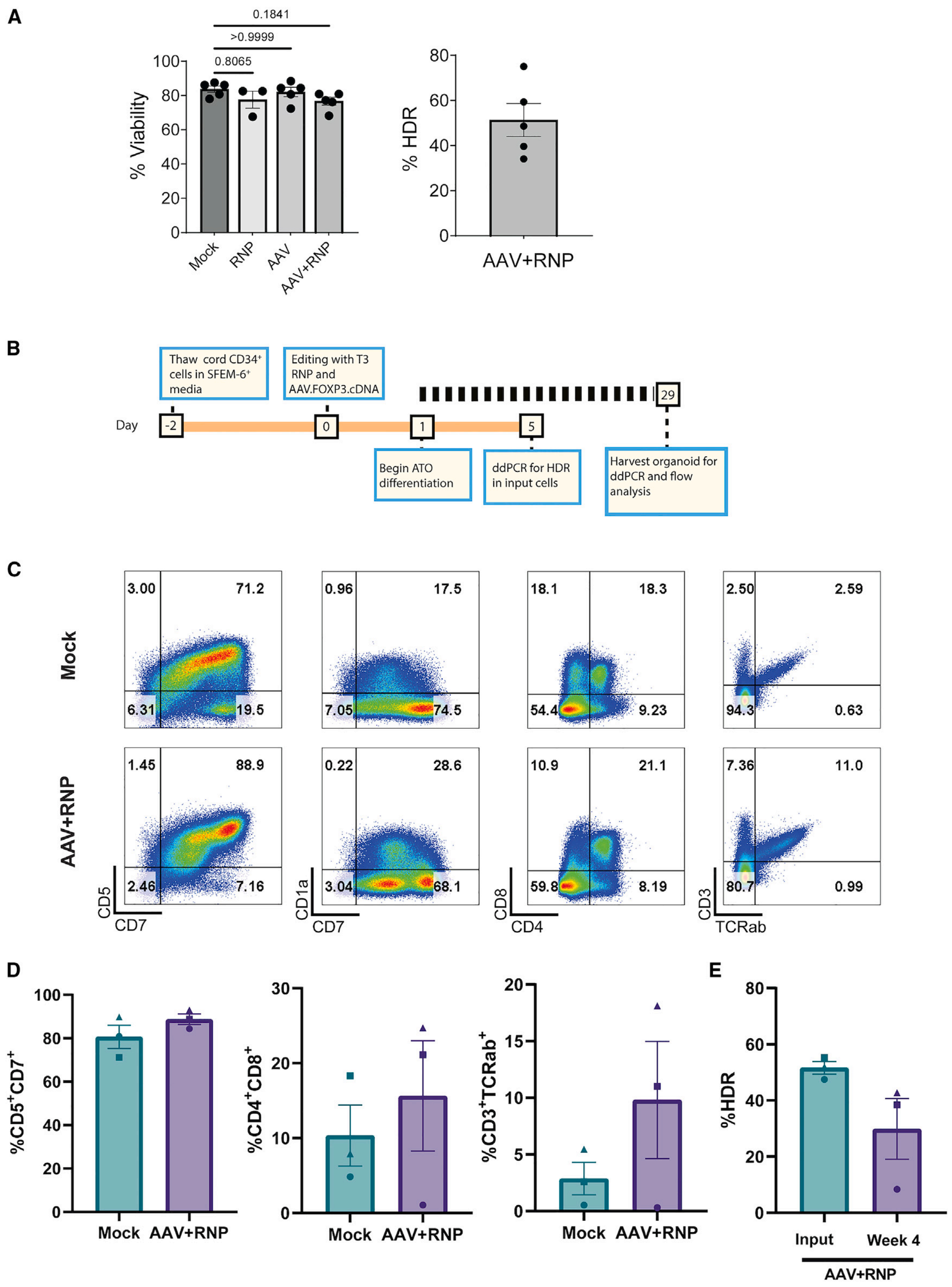

Figure 4. Efficient differentiation of FOXP3-edited HSPCs to T-lineage commitment

(A) Viability (left) and HDR efficiency (right) in healthy donor CB CD34+ HSPCs mock treated (n = 4 male, 1 female), RNP treated (n = 3 male), AAV.FOXP3.cDNA-treated (n = 4 male, 1 female), or RNP + AAV.FOXP3.cDNA edited (n = 4 male, 1 female). Significance determined by Kruskal-Wallis test. (B) Timeline of CB CD34+ HSPC differentiation

(legend continued on next page)

differentiation, we utilized two parallel differentiation strategies: OP9-DL1 co-culture[31] and artificial thymic organoids[32] to differentiate cDNA-edited human HSPCs toward T cell lineage *in vitro*. OP9-DL1 cells are stromal cells (derived from the M-CSF deficient op/op mouse) engineered to express the Notch ligand Delta-like1 (DL1) that can be used to facilitate differentiation of HSPCs into T lymphocytes in response to a combination of key cytokines and notch-dependent signals. As CB-CD34$^+$ cells are more amenable to these differentiation protocols, we cultured and edited healthy donor CB-CD34$^+$ in protocol B conditions. We observed cell viabilities comparable to mPB CD34 $^+$ 24 h after editing (Figure 4A, left). Notably, the average HDR editing rates were moderately higher in CB-CD34$^+$ HSPCs than observed in mPB CD34$^+$ HSPCs (Figure 4A, right). One day after editing, mock-treated and AAV.FOXP3.cDNA-edited CD34$^+$ were put into differentiation systems as outlined in Figures 4B and S7A. At the same time, mock-treated and edited cells were analyzed by flow cytometry for surface expression of CD34, CD45, CD19, CD56, CD14, CD5, CD7, CD1a, CD3, and TCRαß to confirm CD34$^+$ purity and the absence of contaminating T cells or other lin$^+$ cells (Figure S7B). A small amount of mock-treated and edited cells were kept in culture until 5 days after editing to determine the HDR rate in edited cells that were introduced into the differentiation systems.

Cultures were maintained for 28 days then analyzed by flow cytometry for T lineage differentiation (Figures 4C and S7C). After 28 days in co-culture, differentiated cells predominantly reached CD34$^-$CD5$^+$CD7$^+$ pre-T-1 stage (Figures 4D and S7D). Importantly, there was no observable differences between the proportion of pre-T-1 cells in mock-treated compared with edited conditions. Importantly, in the ATO system, we observed a proportion of cells reaching the CD4$^+$ CD8$^+$ double-positive and CD4$^+$ or CD8$^+$ single-positive developmental stages. In addition, we observed a modest amount of CD3$^+$TCRab$^+$ cells in comparable efficiency across mock-treated and AAV+RNP-treated conditions (Figure 4D), consistent with others finding of superior differentiation in the ATO system.[32] Importantly, the proportion of HDR-edited cells at termination of OP9-DL1 differentiation closely matched the input HDR frequency (Figure S7E). We observed greater variation in the proportion of HDR-edited cells recovered after differentiation in the ATO system (Figure 4E). We believe the primary cause of high variability observed in the ATO system reflects the much lower number of input cells used (5,000) relative to OP9-DL1 (250,000) and potential skewing due to limited numbers of lymphoid progenitors and HDR-edited lymphoid progenitors in this smaller input sample.

Together, these results demonstrate that mock-edited and HDR-edited CB-CD34$^+$ HSPCs exhibit similar T lineage differentiation capacity and that the overall proportion of AAV.FOXP3.cDNA HDR-edited cells remains stable during commitment to the T lineage.

Assessment of potential off-target cleavage sites for FOXP3 sgRNA T3

The off-target sites for T3 guide were predicted *in silico* by CCtop-CRISPR-Cas9 target online predictor (Table S2). The top five off-target predicted sites were then interrogated using the Miseq platform. Two of the off-target sites (OT1 and OT4) were within the exons of genes. The top off-target site was Disheveled Binding Antagonist of Beta Catenin 2 (*DACT2*), a protein involved in intracellular signaling pathways during development. The OT4 site is located within the Exostosin Like Glycosyltransferase 1 gene (*EXTL1*), a member of the multiple exostoses family of glycosyltransferases involved in the chain polymerization of heparan sulfate and heparin. The off-target NHEJ rates at both OT1 and OT4 were 0.1%, equivalent to that observed for the mock sample that received no RNPs. The highest off-target cleavage (0.9%) was seen at the OT-II in Solute Carrier Family 2 Member 1 gene, which is the major glucose transporter in the mammalian blood-brain barrier (Figure S6A).

High rates of NHEJ were observed at the *FOXP3* locus (83%), which were equivalent to those determined by the ddPCR assay (94%). The majority of the NHEJ events at the FOXP3 locus were deletions (80%), followed by insertions (15%) and substitutions (10%) (Figures S8A and S8B). A high percentage of NHEJ edits were six nucleotide deletions that constituted 15% of the total NHEJ events (Figures S8C and S8D). Thus, the off-target cleavage rates were significantly lower at <1% for the five off-target sites, while high NHEJ edits were observed at the *FOXP3* locus, confirming that our sgRNAs is largely specific for the target locus.

Efficient editing of IPEX patient CD34$^+$ cells

We obtained a small CB specimen from an IPEX patient bearing an I363V missense mutation located in the FOXP3 forkhead domain that renders the protein incapable of establishing the T_{reg} cell transcriptional program. CD34$^+$ cells were isolated from the frozen sample and editing reagents introduced after 2 days of culturing and prestimulation using protocol B. Notably, ddPCR analysis revealed average HDR editing rate of 34%, although significant differences were observed between the two studies performed, likely reflecting the limited cell numbers and relatively lower viability of the CB sample (Figure 5A).

Characterization of FOXP3 cDNA-edited IPEX patient T cells and healthy donor natural T_{reg} cells

To evaluate expression of the FOXP3 cDNA cassette, we purified and edited healthy donor conventional T (T_{conv}) cells, IPEX patient

toward T lymphocyte lineage in artificial thymic organoid culture system. (C) Representative plots of mock-treated and edited CB CD34$^+$ HSPC-derived cells after 28 days in ATO system. CD5 vs. CD7, CD1a vs. CD7, CD4 vs. CD8, and CD3 vs. TCRαß plots are gated by FSC vs. SSC, SSC-A vs. SSC-W, hCD45$^+$ CD34$^-$ and CD14$^-$CD56$^-$CD19$^-$ to exclude monocytes, natural killer cells, and B cells. (D) Proportion of pre T-1 (CD5$^+$CD7$^+$), CD4$^+$CD8$^+$, and CD3$^+$TCRab cells at the termination of ATO differentiation (gated on hCD45$^+$CD34$^-$ and CD14$^-$CD56$^-$CD19$^-$) (n = 3 male donors, 2 independent studies). (E) Proportion of HDR-edited cells at the initiation and termination of ATO differentiation quantified by ddPCR (n = 3 male donors, 2 independent studies). Bar graphs represent mean ± SEM.

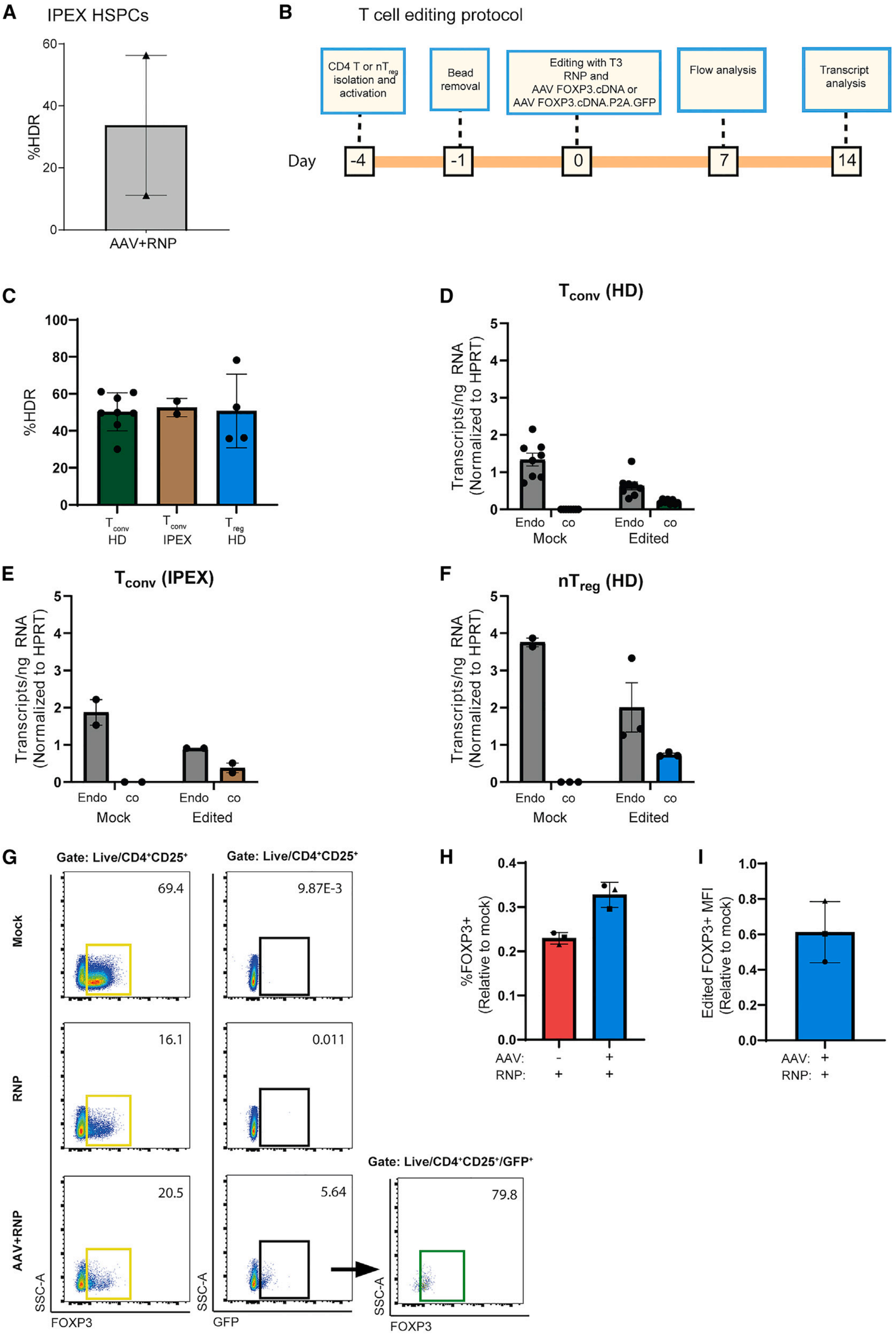

(legend on next page)

T_{conv} cells, and healthy donor natural T_{reg} (nT_{reg}) cells following the experimental timeline outlined in Figure 5B and AAV.FOXP3.cDNA vector diagrammed in Figure 3A. Five days after editing, gDNA was extracted and HDR was quantified via ddPCR. HDR rates were similar across groups: T_{conv} cells (healthy) 50%, T_{conv} cells (IPEX) 52%, and nT_{reg} cells (healthy) 56% (Figure 5C). Fourteen days after editing, we extracted RNA, synthesized cDNA, and quantified transcript levels via ddPCR. Because we utilized a codon-diverged FOXP3 sequence in the AAV.FOXP3.cDNA vector, we were able to distinguish between endogenous and codon-diverged FOXP3 expression. As we achieved approximately 50% editing rates, we predicted approximately equal proportions of coFOXP3 and endoFOXP3 transcripts. However, in edited T_{conv} cells (healthy), T_{conv} cells (IPEX), and nT_{reg} cells (healthy) we measured a ratio of coFOXP3:endogenous FOXP3 transcripts of 0.32, 0.42, and 0.37 respectively (Figures 5D–5F). The reduced level of coFOXP3 transcripts in comparison with endogenous transcripts likely reflects differences in RNA processing efficiency of intron-less FOXP3 cDNA donor vs. the endogenous transcripts.

Next, we performed studies designed to identify FOXP3 protein expression mediated by the FOXP3 cDNA cassette following editing of an enriched nT_{reg} cell population. To specifically identify FOXP3 expression in HDR-edited nT_{reg} cells, we created a FOXP3.cDNA.P2A.GFP donor cassette where cis-linked GFP expression would permit precise flow-based identification of HDR-edited cells. Seven days after editing, we quantified GFP and FOXP3 levels in mock-treated, FOXP3 RNP (knockout), and FOXP3.GFP-edited nT_{reg} cells (Figure 5G). As predicted, GFP expression was observed only in the FOXP3.GFP-edited nT_{reg} cell population. We observed efficient (~77%) FOXP3 knockout in the RNP-treated nT_{reg} cells and a low level of FOXP3 restoration in FOXP3.GFP-edited nT_{reg} cells (Figure 5H). Importantly, within the GFP^+ population in the FOXP3.GFP-edited nT_{reg} cell population, the majority of cells were $FOXP3^+$; and this population exhibited approximately 60% the mean fluorescence intensity of wild-type FOXP3 (Figure 5I). We attempted to perform suppression assays using fluorescence-activated cell sorting (FACS)-sorted GFP^+ populations. However, due to limited cell yields and purity loss during expansion, we did not obtain sufficient viable GFP^+ T_{reg} cells for functional assays.

Together, these data demonstrate *FOXP3* promoter-mediated transcription of coFOXP3 in HDR-edited nT_{reg} cells from healthy control subjects and T_{conv} cells isolated from both IPEX patients and healthy control subjects; we also directly demonstrate expression of exogenous FOXP3 protein in HDR-edited healthy control nT_{reg} cells, albeit at sub-endogenous levels.

DISCUSSION

IPEX syndrome, caused by *FOXP3* mutations, is a devastating disease that leads to substantial mortality. The requirement for strict epigenetic regulation of *FOXP3* necessitates development of a therapeutic approach that preserves the endogenous control elements required to orchestrate both thymus-dependent T_{reg} cell lineage differentiation and maintenance of the T_{reg} cell program *in vivo*. One approach for the treatment of IPEX is enforced expression of FOXP3 in $CD4^+$ T cells by delivering its coding sequence driven via a robust promoter.[19] While such autologous engineered T_{reg} cells may provide temporary clinical benefit, replicating the critical steps in Treg cell lineage programming and selection and generation over time requires thymic repopulation with gene-corrected lymphoid progenitors derived from LT-HSC. Thus, to achieve a cure for IPEX necessitates editing of $CD34^+$ HSC and seamless introduction of the FOXP3 cDNA under transcriptional control of the endogenous promoter. Through optimization of $CD34^+$ culture and HDR editing protocols, we demonstrate efficient insertion of FOXP3 cDNA at the *FOXP3* locus and sustained engraftment of cDNA-edited cells *in vivo*. We show that this approach is feasible using clinically relevant, mobilized $CD34^+$ HSPCs from multiple healthy donors as well as in $CD34^+$ cells derived from an IPEX subject. Further, we show that cDNA-edited $CD34^+$ cells retain the capacity to differentiate into T lineage cells and retain the FOXP3 cDNA cassette *in vitro*. Finally, using healthy donor and IPEX patient T lymphocytes edited with the FOXP3 cDNA cassette, we find FOXP3 cDNA transcripts and FOXP3 protein is expressed at levels that may provide a selective advantage *in vivo*, similar to the expansion of donor-derived T_{reg} cells observed in IPEX patients following non-myeloablative HSCT.[15–18]

Culturing and editing conditions can have a significant impact on the HDR rates and engraftment potential of HSPCs. Culturing $CD34^+$ cells using a low-density editing protocol and small molecules that support HSC survival (protocol B) enabled higher rates of HDR in the edited cells (>40%), while no differences were observed in the

Figure 5. Molecular characterization of IPEX patient HSPCs and T_{conv} cells and healthy donor T_{reg} cells and T_{conv} cells edited with FOXP3 cDNA

(A) HDR efficiency in RNP + AAV.FOXP3.cDNA edited IPEX patient HSPCS (n = 1 donor, 2 independent studies). (B) Timeline and protocol for T_{reg} cells and T_{conv} editing and characterization studies. (C) RNP + AAV.FOXP3.cDNA editing efficiencies in healthy donor-derived T_{conv} cells (n = 8 male donors, 6 independent studies), healthy donor-derived T_{reg} cells (4 male donors, 4 independent studies), and IPEX patient-derived T_{conv} (n = 2 male donors, 2 independent studies) quantified by ddPCR. (D–F) Endogenous (endo) and codon optimized (co) FOXP3 transcript levels in mock-treated and RNP + AAV.FOXP3.cDNA edited. (D) Healthy donor-derived T_{conv} cells (n = 8 male donors, 6 independent studies). (E) IPEX patient T_{conv} cells (n = 2 male donors, 2 independent studies). (F) Healthy donor-derived T_{reg} cells (n = 3 male donors, 2 independent studies) (transcripts quantified by ddPCR and normalized to HPRT control transcript). (G) Representative flow plots of $FOXP3^+$ and GFP^+ populations in nT_{reg} cells isolated from healthy donors. Comparison of unstained mock treated and FOXP3-stained mock-treated, RNP-treated, and RNP + AAV.FOXP3.cDNA.GFP-treated 7 days after editing. (H) Proportion of FOXP3 knockout (RNP-treated nT_{reg} cells) and FOXP3 restoration (RNP + AAV.FOXP3.cDNA.GFP edited) relative to mock treated. Gated on $CD4^+CD25^+$, representative plot highlighted in yellow in (G) (n = 3 male donors, 2 independent studies). (I) Mean fluorescence intensity (MFI) of edited FOXP3 (RNP + AAV.FOXP3.cDNA.GFP-treated nT_{reg} cells, gated on $CD4^+CD25^+GFP^+$) compared with endogenous $FOXP3^+$ MFI (mock-treated, gated on $CD4^+CD25^+$). Representative plot highlighted in green in (G). (n = 3 male donors, 2 independent studies). Bar graphs represent mean ± SEM.

rates of NHEJ edits between the two protocols. Upon transplantation of edited cells into humanized mice, a 2-fold higher engraftment of HDR-edited cells was observed with protocol B compared with protocol A edited cells. A similar low-density protocol was previously reported to improve HDR rates within the LT-HSC population by enforcing G2M or S phases of the cell cycle and facilitated higher engraftment of edited cells *in vivo*.[27] We also observed a 2-fold higher percentage of HSC-enriched HSPCs and a 3-fold higher proportion of HDR-edited (GFP^+) cells within the HSC-enriched HSPC gate recovered from mice further corroborating this hypothesis. Normal lineage distribution was observed with cells edited with either protocol compared with mock controls, suggesting that editing did not negatively impact differentiation *in vivo*.

Recently reported methods to improve editing and engraftment of HDR-edited LT-HSCs include co-transfection of select mRNAs during nuclease delivery. Ferrari et al.[33] have shown improved engraftment of gene-edited LT-HSCs by introduction of dominant negative P53 inhibitor along with adenoviral protein Ad5-E4orf6/7. Transient expression of Ad5-E4orf6/7 triggered an E2F-driven pleiotropic response that facilitated cell-cycle progression and expression of genes encoding for the HDR apparatus leading to increased editing within primitive $CD34^+$ cells. In parallel, the dominant active P53 inhibitor (GSE56),[34] helped to preserve HSC survival or engraftment upon transplantation in immune deficient mice. While not tested in human HSC, another possible approach to enhance HDR is fusion of dominant-negative mutant of 53BP1 to Cas9.[35] 53BP1 enhances HDR by limiting DNA end resection and hindering recruitment of *BRCA1* to the DNA cleavage site.[36,37] By fusing Cas9 activity with 53BP1 inhibition, the authors were able to locally retard NHEJ at the site of the introduced DSB without causing global 53BP1 inhibition. In another study, co-delivery of an engineered ubiquitin variant of an inhibitor of 53BP1 (i53)[38] mRNA and GSE56[34] mRNA with editing reagents enhanced long term correction in X-MEN patient BM-derived $CD34^+$ cells.[39] Robust engraftment of HDR-edited patient cells was observed in the BM of intrahepatic-transplanted, irradiated, newborn NSGS mice in a modulator-dependent manner. While effectively demonstrating modulator efficacy, it remains unclear whether this model accurately predicts HDR-edited HSPC engraftment capability in a clinical setting.

To validate expression and assess the levels of the introduced codon optimized transcript, we edited primary T lymphocytes and nT_{reg} cells from healthy donors. Expression of codon optimized transcripts was readily detected in both HDR-edited T cells and T_{reg} cells. Exogenous cDNA expression was highest in HDR edited T_{reg} cells likely due to the open chromatin landscape in the region compared with conventional $CD4^+$ T cells.[40,41] Our HDR editing methodology also performed similarly using $CD4^+$ T lymphocytes derived from two independent IPEX subjects including CB T cells (isolated from a subject with a I363V mutation) and peripheral blood $CD4^+$ T (from a subject with a polyA region mutation). Despite efficient editing in both healthy control and IPEX patient-derived T cells, exogenous cDNA expression levels comprised between 32% and 42% of wildtype FOXP3. Consistent with our findings, in a separate study by Goodwin et al.,[20] introduction of a FOXP3.cDNA.LNGFR cassette into T_{reg} cells, resulted in sub-endogenous levels of FOXP3 protein expression in edited T_{reg} cells compared with control T_{reg} cells. Lower or absent protein expression has been reported when fully spliced cDNA is introduced into the first coding exon of a target gene.[20,42] This approach precludes the splicing process, often required for optimal transcription and translation. Intronic sequences harbor regulatory elements and their interaction with the splicing machinery can play a critical role in modulating initiation/processivity by RNA polymerase II, pre-mRNA processing, and/or mRNA export.[43] We speculate that, by redesigning AAV HDR cDNA donors to include alternate post-transcriptional elements such as the full WPRE element, a stronger polyadenylation signal and/or candidate intronic elements, that codon optimized FOXP3 transcript and protein expression will reach endogenous expression levels. Such modifications in donor design are likely to be required to achieve consistent T_{reg} cell function.

Investigation of the FOXP3 T3 sgRNA cut site using NGS revealed the indel spectrum in edited cells. Larger deletions (>3 nucleotides) were favored over fewer than three nucleotide deletions which accounted for only 6% of the total NHEJ events. The indel signature of sgRNAs has been utilized by Tatiossian et al.[44] to predict the outcome of HDR frequency and further corroborated using donor templates demonstrating that a larger proportion of nucleotide deletions of more than three nucleotides favor HDR upon template introduction, as was seen with this specific sgRNA. Analysis of the top five predicted off-target sites revealed less than 1% off-target DSBs. Moving the FOXP3 T3 sgRNA toward therapeutic application, however, will necessitate additional unbiased off-target assessments such as GUIDE-seq or alternative methodologies.[45]

An HDR editing strategy similar to that described in our study was utilized to target *FOXP3* locus in T cells (from healthy and IPEX donors) and healthy donor CB-derived HSPCs using CRISPR sgRNAs and rAAV6 vectors.[20] Compared with our findings, Goodwin et al.[20] demonstrated a lower level of engraftment of HDR edited CB progenitors. Consistent with this limited engraftment, purified edited vs. unedited T cells derived from engrafted animals failed to demonstrate suppressive activity *in vitro*. In contrast, we focused primarily on editing and transplantation of HSPCs derived from apheresis of G-CSF-mobilized healthy donors—the HSC cell source anticipated to be utilized for clinical application. In parallel, as in Goodwin et al.,[20] we demonstrate successful HDR editing in T cells from both healthy and IPEX donors. Importantly, here we also demonstrate successful editing of $CD34^+$ cells from IPEX patients. As an alternative therapeutic approach, LV-mediated gene delivery of a FOXP3 expression cassette (utilizing the proximal *FOXP3* promoter and conserved non-coding sequences and FOXP3 cDNA) into murine HSCs, followed by transplantation of purified T cells into neonatal *scurfy* mice (the murine equivalent of IPEX) was shown to limit disease.[9] However, heterogeneous expression correlating with viral copy number (VCN) was observed and high VCNs (>3) were required to reach a therapeutic threshold of transgene expression. Further, the critical

conserved non-coding sequence 2 within the LV sequences did not retain endogenous methylation dynamics. Finally, LV gene therapy has other potential disadvantages including inability to control VCN or integration site, position-effect variegation, and the potential of insertional mutagenesis.

In summary, we demonstrate efficient HDR-based editing of the *FOXP3* locus in control and IPEX patient CD34$^+$ HSPCs. Further, we show that control HDR-edited HSPCs are capable of sustained engraftment *in vivo* in humanized mice. This editing methodology sets the foundation for developing a definitive therapy for IPEX patients. Incorporation of recent advances in the field and additional HDR donor design changes will likely assist in improving outcomes and paving the way for clinical translation.

MATERIALS AND METHODS

Reagent source and category numbers listed in Tables S3–S10 and rAAV6 sequences are listed in Table S11.

Experimental model and subject details

Cell lines

OP9-DL1 cells. The OP9-DL1 stromal cells were cultured in Alpha MEM with Nucleosides (STEMCELL Technologies, Vancouver, Canada) supplemented with 20% fetal bovine serum (FBS) (Omega Scientific, Tarzana, CA, USA).

MS5-hDLL4 cells. MS5 murine stromal cells transduced with a lentiviral vector encoding human DLL4 were a provided by Dr. Gay Crooks (UCLA). Stable expression of DLL4 was confirmed by flow cytometry after multiple weeks in culture. MS5-DLL4 cells were cultured in DMEM (Gibco, Thermo Fisher Scientific, Waltham, MA, USA) + 10% FBS (Omega Scientific).

Primary cells

Mobilized peripheral HSPCs and CB CD34$^+$ cells from healthy donors. Human CD34$^+$ HSPCs enriched from mobilized PBMCs were obtained from the Cooperative Centers of Excellence in Hematology, Fred Hutchinson Cancer Research Center (supported by NIDDK Grant DK106829). CB from healthy donors was purchased from Bloodworks Northwest (Seattle, WA); CD34$^+$ cells were isolated from CB using human CD34 MicroBead Kit (Miltenyi Biotec, Bergisch Gladback, Germany).

PBMC-derived CD4$^+$ T lymphocytes and tT$_{reg}$ cells from healthy donors. PBMCs were obtained from the Cooperative Centers of Excellence in Hematology, Fred Hutchinson Cancer Research Center. Human primary CD4$^+$ T cells and tT$_{reg}$ cells were isolated from thawed PBMCs using negative selection for CD4 and positive selection for CD4$^+$CD127^{low}CD25$^+$ enrichment, respectively (both from STEMCELL Technologies). T cells were cultured in T cell media (RPMI 1640; Gibco) with 20% FBS (Omega Scientific), 10 mM HEPES (Gibco), 2 mM Glutamax (Gibco), 55 μM β-mercaptoethanol (Sigma-Aldrich, St. Louis, MO, USA) supplemented with IL-2 (50 ng/mL, Peprotech, Thermo Fisher Scientific).

IPEX patient samples

CB samples from IPEX patient with I363V mutation and PBMCs from IPEX patient with a polyA mutation (AAUAAA>AAUGAA within the endogenous poly A sequence) were obtained after informed consent using protocols approved by the institutional Review Board of Seattle Children's Research Institute.

Mouse strains

NBSGW mice (NOD.Cg-$Kit^{W-41J}Tyr^{+}Prkdc^{scid}Il2rg^{tm1Wjl}$/ThomJ, Stock 026622, Jackson Laboratory, Bar Harbor, ME, USA) used for the experiments were either purchased from Jackson Laboratory or inbred and maintained in the specific pathogen-free animal facility of the Seattle Children's Research Institute according to Institutional Animal Care and Use Committee and approved protocols.

Method details

sgRNA selection

CRISPR gRNAs targeting *FOXP3* exon 1 were identified using CCTop- CRISPR-Cas9 target online predictor (https://crispr.cos.uni-heidelberg.de/). Top cleaving sgRNAs (T3 and T9) identified from an initial screen were selected for testing in HSPC CD34$^+$ cells. The guides were synthesized as chemically modified 2′-O-methyl analogs with 3′ phosphorothioate internucleotide linkages in the first three 5′ and 3′ terminal residues (Synthego, Redwood City, CA, USA).

AAV6 donor templates and vector production

In-Fusion HD cloning kit (Takara, Kusatsu, Japan) was used to insert PCR amplified fragments into pAAV.GFP (a gift from John T. Gray, Addgene plasmid #32395) replacing the GFP and α-globin polyadenylation site in the original vector. T3 and T9 FOXP3.MND.GFP-targeting vectors contain the MND promoter[46] upstream of a GFP cDNA, followed by WPRE3[47] and SV40 polyadenylation signal elements; this expression cassette was flanked 5′ and 3′ by 0.6 or 0.8 kb *FOXP3* homology arms. The FOXP3.cDNA vector contains the codon-optimized FOXP3 cDNA cassette followed by WPRE3 and SV40pA, flanked by 0.8 kb *FOXP3* homology arms on either side. The FOXP3 cDNA.P2A.GFP vector contains the codon-optimized FOXP3 cDNA cassette followed by a P2A ribosomal skip sequence followed by a promoter-less GFP cDNA sequence with WPRE3 and SV40pA elements. The FOXP3 cDNA.P2A.GFP contains the same 0.8-kb homology arms as the FOXP3 cDNA construct.

AAV6 stocks were produced by transient transfection of HgT1-Adeno, Repcap6,[48] and vector plasmid into HEK 293T cells as previously described.[49] Briefly, 48 h after transfection of the vector and helper plasmids, the cells were harvested, pelleted, and frozen thawed three times. The lysate was then treated with benzonase nuclease, loaded onto an iodixanol density gradient and subjected to ultracentrifugation at 67,000 g in Ti70 rotor (Beckman Coulter, Brea, CA, USA). The virus was extracted from the 60%–40% iodixanol interface, aliquoted, and stored at −80°C. The titers of the AAV stocks were determined by qPCR using primers and probes specific for the viral inverted terminal repeats.[50]

$CD34^+$ cell culture and editing

For culturing $CD34^+$ cells using protocol A, cells were seeded into six-well tissue-culture plates at the density of 1×10^6 cells/mL in HSC-6^+ media composed of CellGenix GMP SCGM media (CellGenix, Sartorius, Gottingen, Germany) with 100 ng/mL each of the following recombinant human cytokines: thrombopoietin, stem cell factor, FLT3 ligand, and IL-6 (all from PeproTech), at 37°C, 5% CO_2, and 5% O_2. Forty-eight hours later, 2×10^5 $CD34^+$ cells were electroporated with ribonucleoprotein (RNP) complexes containing 7.5 pmol Cas9 and 13.8 pmol sgRNA, using either a Neon transfection system (Life Technologies, Thermo Fisher Scientific) or Lonza 4-D nucleofector (Lonza, Basel, Switzerland). The cells were transferred to pre-warmed media at the density of 0.8×10^6 cells/mL following electroporation and AAVs added at MOIs ranging from 0.1 to 2K viral genomes (vg)/cell.

For protocol B, cells were seeded at a density of 0.25×10^6 cells/mL in SFEM-6^+ media (SFEMII as basal media supplemented with the same cytokines as protocol A plus 1 μM StemRegenin1 [STEMCELL Technologies] and 35 nM UM171 [ApexBio, Houston, TX, USA]). Forty-eight hours later, RNPs were nucleofected into 2×10^5 cell using Lonza 4-D nucleofector (Lonza) at the same concentration as protocol A. The cells were plated at 1×10^6 cells/mL concentration post nucleofection and transduced with AAV at MOIs ranging from 0.1 to 2K vg/cell. Sixteen hours after transfection, cells were diluted to a density of 2.5×10^5 cells/mL.

The cells were cultured for 5 days after dual delivery of RNPs and AAV. SpyFi Cas9 (Aldevron, Madison, WI, USA) nuclease was used in both protocols. Flow cytometry analysis was performed 1, 2, and 5 days after editing, following which the cells were pelleted and gDNA extraction performed using Qiagen Dneasy Blood and tissue Kit (Qiagen, Hilden, Germany). Editing was performed using the same protocol as above for IPEX I363V cord-derived $CD34^+$ cells.

Primary and patient-derived $CD4^+$ T and nT_{reg} cell culture and editing

Human primary $CD4^+$ T cells and nT_{reg} cells isolated from thawed PBMCs as described above were activated with Dynabeads Human T-expander beads CD3/CD28 (Gibco) at a 3:1 bead to cell ratio for 72 h. After beads were removed, the cells further rested overnight in T cell media followed by nucleofection of 20 pmol Cas9 and 50 pmol sgRNA complexes using Lonza 4D-nucleofector. Donor AAVs were added to the cultures immediately post nucleofection at 15%–20% of the culture volume. After an approximately 24-h incubation at 37°C, fresh media was added to cultures to dilute the AAV to 7.5%–10% of the culture volume. Cells were then split every 2–3 days until day 14 after editing. $CD4^+$ T cells from a FOXP3 poly A mutation and IPEX I363V IPEX patients were also edited as described above for healthy donors.

For nT_{reg} cell phenotyping, cells were surface stained for flow cytometry with the following antibodies: CD4-BV605, CD25-PECy7, and CD127-BV510. Intracellular FOXP3 staining with FOXP3-PE antibody was performed after fixation and permeabilization with True-Nuclear Transcription Factor buffer set (BioLegend, San Diego, CA, USA).

ddPCR analysis for determination of NHEJ rates

PCR amplicons spanning the guide cleavage site were generated with the NHEJ probe binding to the guide cleavage site. A control amplicon of similar size was generated from another region of the *FOXP3* gene. The PCR reactions were partitioned into droplets using a QX200 Droplet Generator (Bio-Rad, Hercules, CA, USA). Amplification was performed using ddPCR Supermix for Probes without UTP (Bio-Rad), 900 nM of primers (IDT, Coralville, IA, USA), 250 nM probe (IDT), and 50 ng genomic DNA. Droplets were analyzed using the QX200 ddPCR System (Bio-Rad) and analyzed using QuantaSoft software (Bio-Rad). The NHEJ rates were calculated using the formula:

$$\Big(\big(\text{signal from NHEJ probe/signal from control probe}\big)_{\text{mock sample}} - \big(\text{signal from NHEJ probe/signal from control probe}\big)_{\text{RNP treated sample}}\Big) * 100.$$

ddPCR analysis for determination of targeted integration

Genomic DNA was extracted from cultured cells and an HDR amplicon was generated by in-out ddPCR using one primer within the AAV construct and another outside the region of homology. An amplicon for either *ActB* (1.3 kb) or *CCR5* (1.5 kb) was generated to serve as the control. Probes for both amplicons were labeled with FAM and the reactions were performed in separate wells. The ddPCR was performed as described in the section above.

RNA extraction and transcript analysis in edited $CD4^+$ T lymphocytes

RNA was extracted using RNeasy mini kit (Qiagen) from cultured T lymphocytes 14 days after editing. Complementary DNA was synthesized using Maxima First Strand cDNA Synthesis Kit (Thermo Fisher Scientific) utilizing 10 ng input RNA. Two microliters cDNA was used in three separate ddPCR reactions to detect codon optimized FOXP3, endogenous FOXP3, and control HPRT transcripts using in-house designed or Taqman gene expression assays (Thermo Fisher Scientific). All reactions were performed in duplicates. Transcript concentration was quantified with the following formula:

$$coFOXP3_{Mock,Treg} = \frac{coFOXP3_{Mock,Treg}}{HPRT_{Mock,Treg}}$$

$$Endogenous\ FOXP3_{Mock,Treg} = \frac{Endogenous\ FOXP3_{Mock,Treg}}{HPRT_{Mock,Treg}}$$

Xenotransplantation of edited $CD34^+$ cells into NBSGW mice

NBSGW mice (NOD.Cg-Kit^{W-41J} Tyr^+ $Prkdc^{scid}$ $Il2rg^{tm1Wjl}$/ThomJ, Stock 026622, Jackson Laboratory) used for the experiments were either purchased from Jackson Laboratory or inbred and maintained in the specific pathogen-free animal facility of Seattle Children's

Research Institute according to Institutional Animal Care and Use Committee and approved protocols. Mock-treated or edited HSPCs treated with either protocol A or B were transplanted to NBSGW recipient mice one day after editing. The recipient mice were treated with 12.5 mg/kg clinical grade Busulfan (Otsuka America Pharmaceutical, Rockville, MD, USA) intraperitoneally 24 h prior to human stem cell transfer followed by retro-orbital injections of 1–2 $\times$ 10^6 $CD34^+$ cells per animal. The transplanted mice were sacrificed 12–16 weeks after transfer, and cells harvested from BM and spleens were analyzed using flow cytometry on LSR II flow cytometer (BD Biosciences, San Jose, CA, USA). To assess engraftment of edited cells in various hematopoietic lineages within the BM and spleen, cells were stained with the following fluorophore-conjugated antibodies: human and mouse CD45, CD33, and CD19. To assess the HSC phenotype, cells were stained with the following fluorophore-conjugated antibodies: CD34, CD38, CD90, and CD133.

In vitro *differentiation of cord-derived $CD34^+$ cells in OP9-DL1 monolayer*

CB $CD34^+$ cells were thawed and edited as described previously for adult mobilized $CD34^+$ cells. One day post-editing, 250,000 $CD34^+$ cells were cocultured on confluent OP9-DL1 stromal cells in Alpha MEM with Nucleosides (STEMCELL Technologies) supplemented with 20% FBS and recombinant human cytokines (Peprotech) IL-7 (10 ng/mL) and FLT3L (10 ng/mL). Input cells were simultaneously analyzed for surface expression of hCD45, CD34, CD14, CD56, CD19, CD1a, CD7, CD3, TCRαß, CD4, and CD8. We kept 150,000 $CD34^+$ in stem cell media and gDNA was extracted 5 days to quantify input cell HDR by ddPCR. $CD34^+$ co-cultures were re-plated onto fresh stromal cells every 3–4 days. After 28 days of co-culture, the cord-derived cells were analyzed for surface expression of hCD45, CD34, CD14, CD56, CD19, CD1a, CD7, CD3, TCRαß, CD4, and CD8. Genomic DNA was extracted from culture and targeted integration of FOXP3 cDNA was quantified by ddPCR.

In vitro *differentiation of cord-derived $CD34^+$ cells in ATO system*

CB $CD34^+$ cells were thawed and edited as described previously for adult mobilized $CD34^+$ cells. One day after editing, 5E4 $CD34^+$ were differentiated in artificial thymic organoid as previously described.[32] Input cells were simultaneously analyzed for surface expression of hCD45, CD34, CD14, CD56, CD19, CD1a, CD7, CD3, TCRαß, CD4, snf CD8. $CD34^+$ cells were also kept in stem cell media and gDNA was extracted 5 days after editing to quantify input cell HDR by ddPCR. After four weeks, ATO cultures were harvested and analyzed by FACs for T lineage differentiation and ddPCR for HDR.

Off- and on-target cleavage validation using Miseq

Off-target cleavage sites for guide T3 were determined using CCTop- CRISPR-Cas9 target online predictor. The top five predicted off target sites (Table S2) along with the target *FOXP3* site were amplified using 200 ng input DNA from two edited donor $CD34^+$ cells using MiSeq oligos and PrimeSTAR GXL DNA polymerase (Clontech, Takara). The above-mentioned amplifications were also performed on donor $CD34^+$ in parallel without delivering any editing reagents to serve as a control. The samples were purified using Agencourt AMPure XP (Beckman Coulter) and analyzed on PAGE gel. The samples were quantified on Qubit (Thermo Fisher Scientific), pooled and analyzed on MiSeq 500 CycleV2 kit (Illumina, San Diego, CA, USA). Data mining was performed with Crispresso2 algorithm.[51]

Quantification and statistical analysis

Statistical analysis was performed using GraphPad Prism software (GraphPad).

DATA AND CODE AVAILABILITY

Materials described here will be provided upon request upon execution of a material transfer agreement with Seattle Children's Research Institute. Human cells will not be provided. Data generated and analyzed in this work are available from the corresponding author on reasonable request.

SUPPLEMENTAL INFORMATION

Supplemental information can be found online at https://doi.org/10.1016/j.omtm.2023.101183.

ACKNOWLEDGMENTS

The authors thank Socheath Khim and Jennifer Haddock for technical and administrative support, respectively. The authors thank Eric Allenspach and Troy Torgerson for collection of cord blood and peripheral blood samples from IPEX subjects. The authors thank Gay Crooks (University of California Los Angeles) for providing MS5-DLL4 cells. This work was supported in part by the Seattle Children's Research Institute (SCRI) Program for Cell and Gene Therapy (PCGT), the Children's Guild Association Endowed Chair in Pediatric Immunology (to D.J.R.), and the Hansen Investigator in Pediatric Innovation Endowment (to D.J.R.).

AUTHOR CONTRIBUTIONS

D.J.R., S.S., and I.F.K. conceptualized the study; S.S., I.F.K., C.P., M.Q.D., Y.H., and E.L. performed experiments and/or analyzed data; I.F.K., K.M.S., C.P., and D.J.R. wrote the manuscript; C.P. and D.J.R. revised the manuscript; I.F.K., C.P., and S.S. prepared the figures; funding was acquired by D.J.R.

DECLARATION OF INTERESTS

S.S., I.F.K, Y.H., K.M.S., and D.J.R hold provisional patent PCT WO2019210042 entitled "Expression of FOXP3 in edited CD34+ cells" related to this work. Another patent, "Expression of Human FOXP3 in Gene Edited T Cells," has also been filed with Y.H., K.M.S., I.F.K, and D.J.R. as authors.

REFERENCES

1. Powell, B.R., Buist, N.R., and Stenzel, P. (1982). An X-linked syndrome of diarrhea, polyendocrinopathy, and fatal infection in infancy. J. Pediatr. *100*, 731–737.

2. Chatila, T.A., Blaeser, F., Ho, N., Lederman, H.M., Voulgaropoulos, C., Helms, C., and Bowcock, A.M. (2000). JM2, encoding a fork head-related protein, is mutated

in X-linked autoimmunity-allergic disregulation syndrome. J. Clin. Invest. *106*, R75–R81.

3. Bennett, C.L., Christie, J., Ramsdell, F., Brunkow, M.E., Ferguson, P.J., Whitesell, L., Kelly, T.E., Saulsbury, F.T., Chance, P.F., and Ochs, H.D. (2001). The immune dysregulation, polyendocrinopathy, enteropathy, X-linked syndrome (IPEX) is caused by mutations of FOXP3. Nat. Genet. *27*, 20–21.
4. Barzaghi, F., Amaya Hernandez, L.C., Neven, B., Ricci, S., Kucuk, Z.Y., Bleesing, J.J., Nademi, Z., Slatter, M.A., Ulloa, E.R., Shcherbina, A., et al. (2018). Long-term follow-up of IPEX syndrome patients after different therapeutic strategies: An international multicenter retrospective study. J. Allergy Clin. Immunol. *141*, 1036–1049.e5.
5. Kucuk, Z.Y., Bleesing, J.J., Marsh, R., Zhang, K., Davies, S., and Filipovich, A.H. (2016). A challenging undertaking: Stem cell transplantation for immune dysregulation, polyendocrinopathy, enteropathy, X-linked (IPEX) syndrome. J. Allergy Clin. Immunol. *137*, 953–955.e4.
6. Passerini, L., Rossi Mel, E., Sartirana, C., Fousteri, G., Bondanza, A., Naldini, L., Roncarolo, M.G., and Bacchetta, R. (2013). CD4(+) T cells from IPEX patients convert into functional and stable regulatory T cells by FOXP3 gene transfer. Sci. Transl. Med. *5*, 215ra174.
7. Bluestone, J.A., Buckner, J.H., Fitch, M., Gitelman, S.E., Gupta, S., Hellerstein, M.K., Herold, K.C., Lares, A., Lee, M.R., Li, K., et al. (2015). Type 1 diabetes immunotherapy using polyclonal regulatory T cells. Sci. Transl. Med. *7*, 315ra189.
8. Brunstein, C.G., Miller, J.S., Cao, Q., McKenna, D.H., Hippen, K.L., Curtsinger, J., Defor, T., Levine, B.L., June, C.H., Rubinstein, P., et al. (2011). Infusion of ex vivo expanded T regulatory cells in adults transplanted with umbilical cord blood: safety profile and detection kinetics. Blood *117*, 1061–1070.
9. Masiuk, K.E., Laborada, J., Roncarolo, M.G., Hollis, R.P., and Kohn, D.B. (2019). Lentiviral Gene Therapy in HSCs Restores Lineage-Specific Foxp3 Expression and Suppresses Autoimmunity in a Mouse Model of IPEX Syndrome. Cell Stem Cell *24*, 309–317.e7.
10. Jinek, M., Chylinski, K., Fonfara, I., Hauer, M., Doudna, J.A., and Charpentier, E. (2012). A programmable dual-RNA-guided DNA endonuclease in adaptive bacterial immunity. Science *337*, 816–821.
11. Cong, L., Ran, F.A., Cox, D., Lin, S., Barretto, R., Habib, N., Hsu, P.D., Wu, X., Jiang, W., Marraffini, L.A., and Zhang, F. (2013). Multiplex genome engineering using CRISPR/Cas systems. Science *339*, 819–823.
12. Zheng, Y., Josefowicz, S., Chaudhry, A., Peng, X.P., Forbush, K., and Rudensky, A.Y. (2010). Role of conserved non-coding DNA elements in the Foxp3 gene in regulatory T-cell fate. Nature *463*, 808–812.
13. Baron, U., Floess, S., Wieczorek, G., Baumann, K., Grützkau, A., Dong, J., Thiel, A., Boeld, T.J., Hoffmann, P., Edinger, M., et al. (2007). DNA demethylation in the human FOXP3 locus discriminates regulatory T cells from activated FOXP3(+) conventional T cells. Eur. J. Immunol. *37*, 2378–2389.
14. Lee, B.C., Lozano, R.J., and Dunbar, C.E. (2021). Understanding and overcoming adverse consequences of genome editing on hematopoietic stem and progenitor cells. Mol. Ther. *29*, 3205–3218.
15. Horino, S., Sasahara, Y., Sato, M., Niizuma, H., Kumaki, S., Abukawa, D., Sato, A., Imaizumi, M., Kanegane, H., Kamachi, Y., et al. (2014). Selective expansion of donor-derived regulatory T cells after allogeneic bone marrow transplantation in a patient with IPEX syndrome. Pediatr. Transplant. *18*, E25–E30.
16. Seidel, M.G., Fritsch, G., Lion, T., Jürgens, B., Heitger, A., Bacchetta, R., Lawitschka, A., Peters, C., Gadner, H., and Matthes-Martin, S. (2009). Selective engraftment of donor CD4+25high FOXP3-positive T cells in IPEX syndrome after nonmyeloablative hematopoietic stem cell transplantation. Blood *113*, 5689–5691.
17. Kasow, K.A., Morales-Tirado, V.M., Wichlan, D., Shurtleff, S.A., Abraham, A., Persons, D.A., and Riberdy, J.M. (2011). Therapeutic *in vivo* selection of thymic-derived natural T regulatory cells following non-myeloablative hematopoietic stem cell transplant for IPEX. Clin. Immunol. *141*, 169–176.
18. Di Nunzio, S., Cecconi, M., Passerini, L., McMurchy, A.N., Baron, U., Turbachova, I., Vignola, S., Valencic, E., Tommasini, A., Junker, A., et al. (2009). Wild-type FOXP3 is selectively active in CD4+CD25(hi) regulatory T cells of healthy female carriers of different FOXP3 mutations. Blood *114*, 4138–4141.
19. Honaker, Y., Hubbard, N., Xiang, Y., Fisher, L., Hagin, D., Sommer, K., Song, Y., Yang, S.J., Lopez, C., Tappen, T., et al. (2020). Gene editing to induce FOXP3 expression in human CD4(+) T cells leads to a stable regulatory phenotype and function. Sci. Transl. Med. *12*, eaay6422.
20. Goodwin, M., Lee, E., Lakshmanan, U., Shipp, S., Froessl, L., Barzaghi, F., Passerini, L., Narula, M., Sheikali, A., Lee, C.M., et al. (2020). CRISPR-based gene editing enables FOXP3 gene repair in IPEX patient cells. Sci. Adv. *6*, eaaz0571.
21. Gambineri, E., Ciullini Mannurita, S., Hagin, D., Vignoli, M., Anover-Sombke, S., DeBoer, S., Segundo, G.R.S., Allenspach, E.J., Favre, C., Ochs, H.D., and Torgerson, T.R. (2018). Clinical, Immunological, and Molecular Heterogeneity of 173 Patients With the Phenotype of Immune Dysregulation, Polyendocrinopathy, Enteropathy, X-Linked (IPEX) Syndrome. Front. Immunol. *9*, 2411.
22. Torgerson, T.R., Linane, A., Moes, N., Anover, S., Mateo, V., Rieux-Laucat, F., Hermine, O., Vijay, S., Gambineri, E., Cerf-Bensussan, N., et al. (2007). Severe food allergy as a variant of IPEX syndrome caused by a deletion in a noncoding region of the FOXP3 gene. Gastroenterology *132*, 1705–1717.
23. Gavin, M.A., Torgerson, T.R., Houston, E., DeRoos, P., Ho, W.Y., Stray-Pedersen, A., Ocheltree, E.L., Greenberg, P.D., Ochs, H.D., and Rudensky, A.Y. (2006). Single-cell analysis of normal and FOXP3-mutant human T cells: FOXP3 expression without regulatory T cell development. Proc. Natl. Acad. Sci. USA *103*, 6659–6664.
24. Vakulskas, C.A., Dever, D.P., Rettig, G.R., Turk, R., Jacobi, A.M., Collingwood, M.A., Bode, N.M., McNeill, M.S., Yan, S., Camarena, J., et al. (2018). A high-fidelity Cas9 mutant delivered as a ribonucleoprotein complex enables efficient gene editing in human hematopoietic stem and progenitor cells. Nat. Med. *24*, 1216–1224.
25. Mohrin, M., Bourke, E., Alexander, D., Warr, M.R., Barry-Holson, K., Le Beau, M.M., Morrison, C.G., and Passegué, E. (2010). Hematopoietic stem cell quiescence promotes error-prone DNA repair and mutagenesis. Cell Stem Cell *7*, 174–185.
26. Pietras, E.M., Warr, M.R., and Passegué, E. (2011). Cell cycle regulation in hematopoietic stem cells. J. Cell Biol. *195*, 709–720.
27. Charlesworth, C.T., Camarena, J., Cromer, M.K., Vaidyanathan, S., Bak, R.O., Carte, J.M., Potter, J., Dever, D.P., and Porteus, M.H. (2018). Priming Human Repopulating Hematopoietic Stem and Progenitor Cells for Cas9/sgRNA Gene Targeting. Mol. Ther. Nucleic Acids *12*, 89–104.
28. Boitano, A.E., Wang, J., Romeo, R., Bouchez, L.C., Parker, A.E., Sutton, S.E., Walker, J.R., Flaveny, C.A., Perdew, G.H., Denison, M.S., et al. (2010). Aryl hydrocarbon receptor antagonists promote the expansion of human hematopoietic stem cells. Science *329*, 1345–1348.
29. Fares, I., Chagraoui, J., Gareau, Y., Gingras, S., Ruel, R., Mayotte, N., Csaszar, E., Knapp, D.J.H.F., Miller, P., Ngom, M., et al. (2014). Cord blood expansion. Pyrimidoindole derivatives are agonists of human hematopoietic stem cell self-renewal. Science *345*, 1509–1512.
30. Orford, K.W., and Scadden, D.T. (2008). Deconstructing stem cell self-renewal: genetic insights into cell-cycle regulation. Nat. Rev. Genet. *9*, 115–128.
31. La Motte-Mohs, R.N., Herer, E., and Zúñiga-Pflücker, J.C. (2005). Induction of T-cell development from human cord blood hematopoietic stem cells by Delta-like 1 *in vitro*. Blood *105*, 1431–1439.
32. Montel-Hagen, A., Tsai, S., Seet, C.S., and Crooks, G.M. (2022). Generation of Artificial Thymic Organoids from Human and Murine Hematopoietic Stem and Progenitor Cells. Curr. Protoc. *2*, e403.
33. Ferrari, S., Jacob, A., Beretta, S., Unali, G., Albano, L., Vavassori, V., Cittaro, D., Lazarevic, D., Brombin, C., Cugnata, F., et al. (2020). Efficient gene editing of human long-term hematopoietic stem cells validated by clonal tracking. Nat. Biotechnol. *38*, 1298–1308.
34. Ossovskaya, V.S., Mazo, I.A., Chernov, M.V., Chernova, O.B., Strezoska, Z., Kondratov, R., Stark, G.R., Chumakov, P.M., and Gudkov, A.V. (1996). Use of genetic suppressor elements to dissect distinct biological effects of separate p53 domains. Proc. Natl. Acad. Sci. USA *93*, 10309–10314.
35. Jayavaradhan, R., Pillis, D.M., Goodman, M., Zhang, F., Zhang, Y., Andreassen, P.R., and Malik, P. (2019). CRISPR-Cas9 fusion to dominant-negative 53BP1 enhances HDR and inhibits NHEJ specifically at Cas9 target sites. Nat. Commun. *10*, 2866.
36. Panier, S., and Boulton, S.J. (2014). Double-strand break repair: 53BP1 comes into focus. Nat. Rev. Mol. Cell Biol. *15*, 7–18.
37. Bothmer, A., Robbiani, D.F., Feldhahn, N., Gazumyan, A., Nussenzweig, A., and Nussenzweig, M.C. (2010). 53BP1 regulates DNA resection and the choice between

classical and alternative end joining during class switch recombination. J. Exp. Med. *207*, 855–865.

38. Canny, M.D., Moatti, N., Wan, L.C.K., Fradet-Turcotte, A., Krasner, D., Mateos-Gomez, P.A., Zimmermann, M., Orthwein, A., Juang, Y.C., Zhang, W., et al. (2018). Inhibition of 53BP1 favors homology-dependent DNA repair and increases CRISPR-Cas9 genome-editing efficiency. Nat. Biotechnol. *36*, 95–102.

39. De Ravin, S.S., Brault, J., Meis, R.J., Liu, S., Li, L., Pavel-Dinu, M., Lazzarotto, C.R., Liu, T., Koontz, S.M., Choi, U., et al. (2021). Enhanced homology-directed repair for highly efficient gene editing in hematopoietic stem/progenitor cells. Blood *137*, 2598–2608.

40. Kim, H.P., and Leonard, W.J. (2007). CREB/ATF-dependent T cell receptor-induced FoxP3 gene expression: a role for DNA methylation. J. Exp. Med. *204*, 1543–1551.

41. Janson, P.C.J., Winerdal, M.E., Marits, P., Thörn, M., Ohlsson, R., and Winqvist, O. (2008). FOXP3 promoter demethylation reveals the committed Treg population in humans. PLoS One *3*, e1612.

42. Sweeney, C.L., Zou, J., Choi, U., Merling, R.K., Liu, A., Bodansky, A., Burkett, S., Kim, J.W., De Ravin, S.S., and Malech, H.L. (2017). Targeted Repair of CYBB in X-CGD iPSCs Requires Retention of Intronic Sequences for Expression and Functional Correction. Mol. Ther. *25*, 321–330.

43. Le Hir, H., Nott, A., and Moore, M.J. (2003). How introns influence and enhance eukaryotic gene expression. Trends Biochem. Sci. *28*, 215–220.

44. Tatiossian, K.J., Clark, R.D.E., Huang, C., Thornton, M.E., Grubbs, B.H., and Cannon, P.M. (2021). Rational Selection of CRISPR-Cas9 Guide RNAs for Homology-Directed Genome Editing. Mol. Ther. *29*, 1057–1069.

45. Tsai, S.Q., Nguyen, N.T., Malagon-Lopez, J., Topkar, V.V., Aryee, M.J., and Joung, J.K. (2017). CIRCLE-seq: a highly sensitive *in vitro* screen for genome-wide CRISPR-Cas9 nuclease off-targets. Nat. Methods *14*, 607–614.

46. Challita, P.M., Skelton, D., el-Khoueiry, A., Yu, X.J., Weinberg, K., and Kohn, D.B. (1995). Multiple modifications in cis elements of the long terminal repeat of retroviral vectors lead to increased expression and decreased DNA methylation in embryonic carcinoma cells. J. Virol. *69*, 748–755.

47. Choi, J.H., Yu, N.K., Baek, G.C., Bakes, J., Seo, D., Nam, H.J., Baek, S.H., Lim, C.S., Lee, Y.S., and Kaang, B.K. (2014). Optimization of AAV expression cassettes to improve packaging capacity and transgene expression in neurons. Mol. Brain *7*, 17.

48. Rutledge, E.A., Halbert, C.L., and Russell, D.W. (1998). Infectious clones and vectors derived from adeno-associated virus (AAV) serotypes other than AAV type 2. J. Virol. *72*, 309–319.

49. Khan, I.F., Hirata, R.K., and Russell, D.W. (2011). AAV-mediated gene targeting methods for human cells. Nat. Protoc. *6*, 482–501.

50. Aurnhammer, C., Haase, M., Muether, N., Hausl, M., Rauschhuber, C., Huber, I., Nitschko, H., Busch, U., Sing, A., Ehrhardt, A., and Baiker, A. (2012). Universal real-time PCR for the detection and quantification of adeno-associated virus serotype 2-derived inverted terminal repeat sequences. Hum. Gene Ther. Methods *23*, 18–28.

51. Pinello, L., Canver, M.C., Hoban, M.D., Orkin, S.H., Kohn, D.B., Bauer, D.E., and Yuan, G.C. (2016). Analyzing CRISPR genome-editing experiments with CRISPResso. Nat. Biotechnol. *34*, 695–697.

OMTM, Volume *32*

Supplemental information

Efficient and sustained *FOXP3* locus editing in hematopoietic stem cells as a therapeutic approach for IPEX syndrome

Swati Singh, Cole M. Pugliano, Yuchi Honaker, Aidan Laird, M. Quinn DeGottardi, Ezra Lopez, Stefan Lachkar, Claire Stoffers, Karen Sommer, Iram F. Khan, and David J. Rawlings

Supplemental Materials

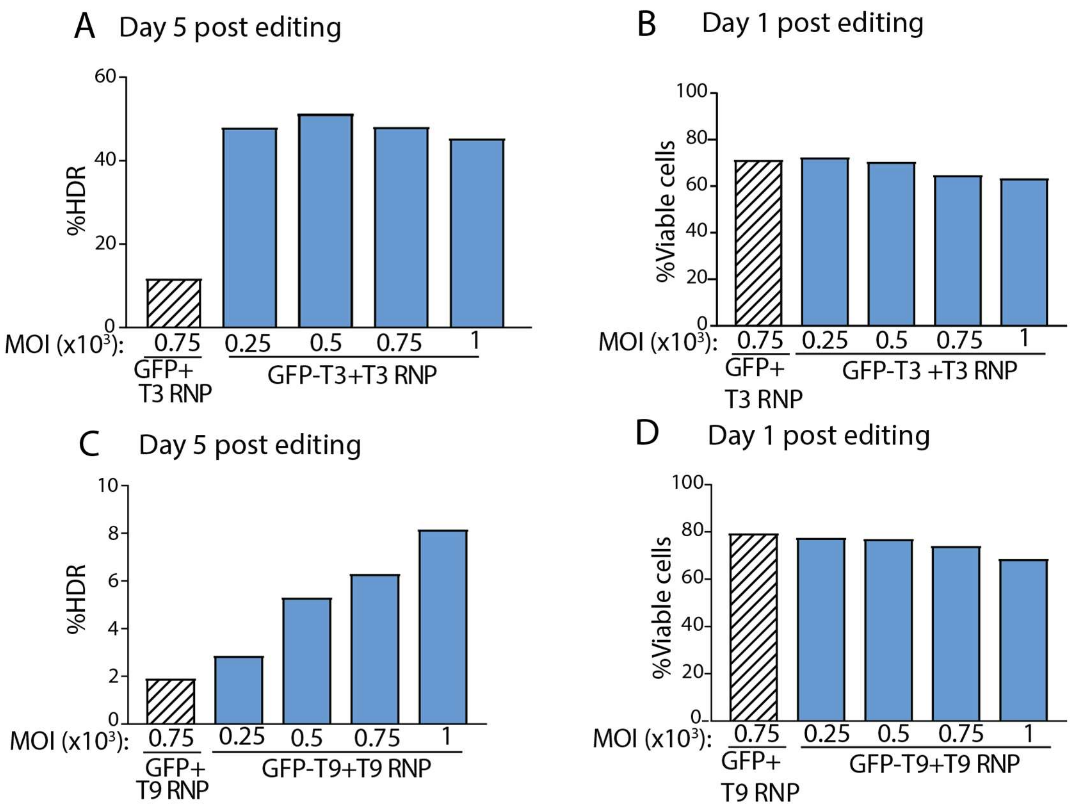

Figure S1. Improved editing rates with gRNA specific AAV donor templates. (A, B) Bar graphs depicting HDR rates 5 days post-editing **(A)** or cell viabilities determined by forward and side scatter one day post editing **(B)** using T3 RNP along with the common AAV.GFP donor (black striped bars) or the T3 specific AAV (GFP-T3; blue bars) at the indicated MOIs. The common AAV.GFP donor harbored a 0.2 kb deletion to disrupt the binding sites for both sgRNAs. **(C, D)** Bar graphs depicting HDR rates five days post-editing **(C)** or cell viabilities determined by forward and side scatter one day post editing **(D)** using T9 RNP along with the common AAV.GFP donor (black striped bars) or the T9 specific AAV (GFP-T9; blue bars) at the indicated MOIs.

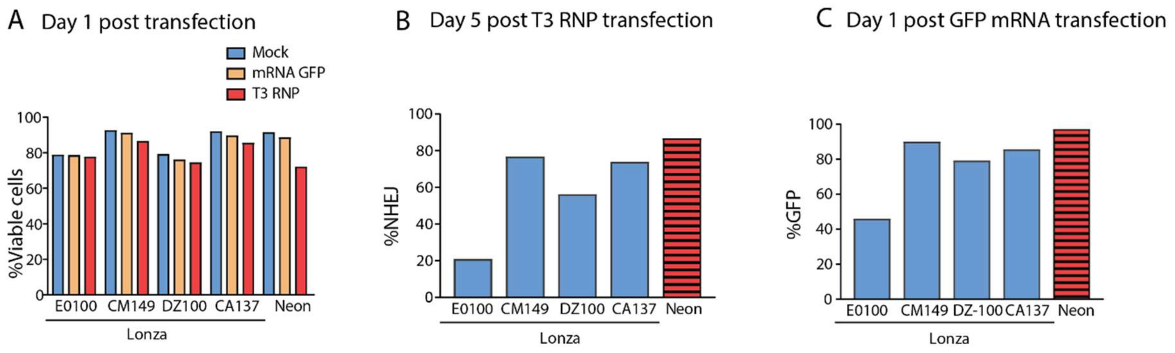

Figure S2. Comparison of cell viabilities and transfection efficiencies when nucleofecting human CD34$^+$ cells with Lonza or electroporating with Neon. Adult CD34$^+$ cells were cultured in HSC media for 48 hours using protocol A, followed by electroporation using Neon or nucleofection with Lonza. The cells were either mock transfected, transfected with 1µg of GFP mRNA or with T3 RNP. Program CM149 allowed highest transfection of GFP mRNA in greater than 90% of the cells without any impact on cell viability and was thus employed for all future nucleofections. **(A)** Bar graphs show cell viability one day post transfection as assessed by flow cytometry forward and side scatter. **(B)** Bar graphs showing % NHEJ assessed by ddPCR of gDNA from CD34$^+$ cells 5 days post transfection with T3 RNP. **(C)** Bar graphs showing % transfection assessed by flow cytometry one day post-delivery of GFP mRNA.

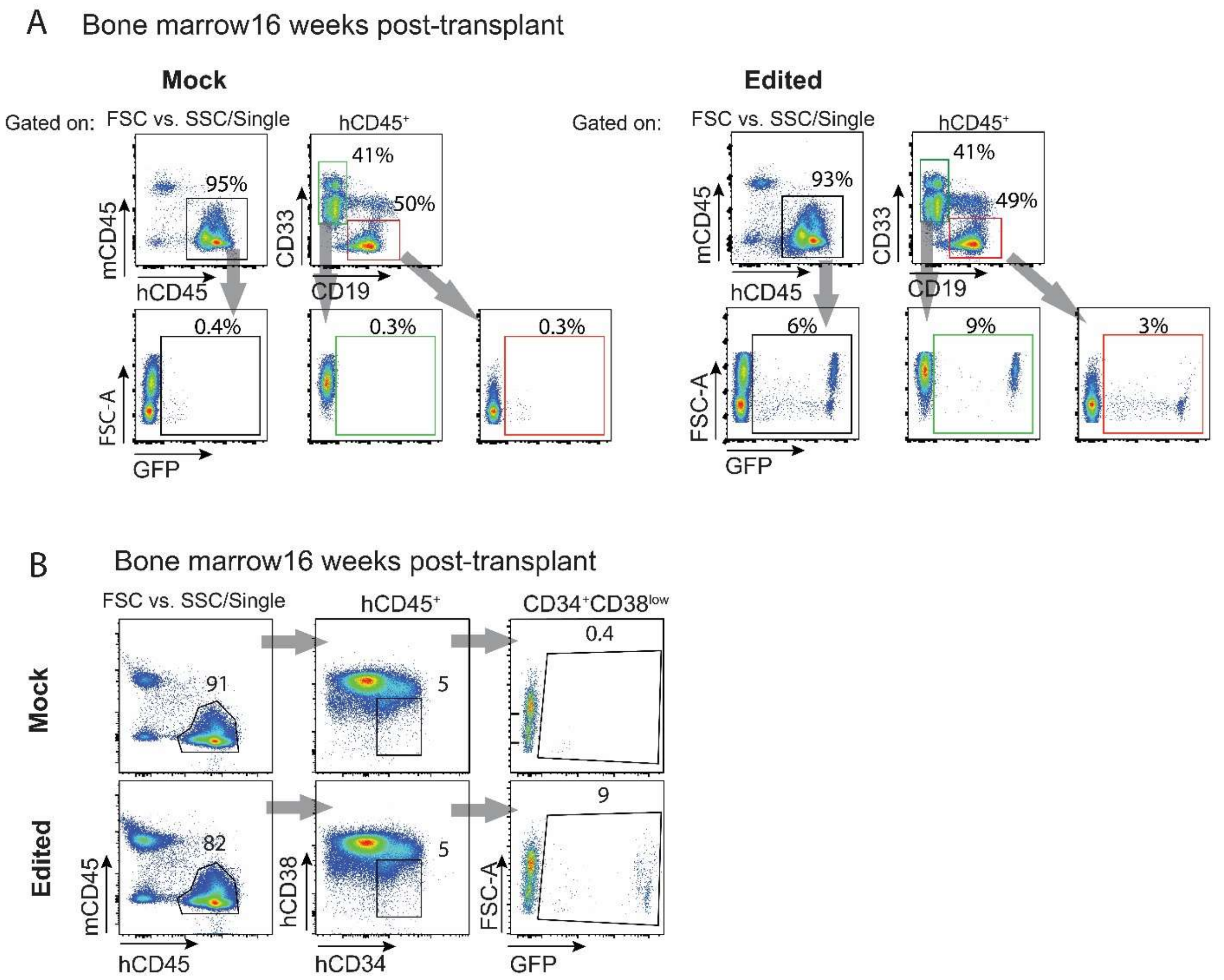

Figure S3. Gating strategy for analysis of bone marrow in GFP-T3 in vivo studies (Figure 2). **(A)** Gating strategy in mock-treated (left) and edited (right) animals to quantify human chimerism (Figure 2B) and B cell/myeloid compartments (Figure 2D). HDR frequency (GFP+) in bulk hCD45+ (Figure 2C), B cell (Figure 2E), and myeloid (Figure 2F) compartments shown with arrows drawn from parental gate. hCD45 vs. mCD45 was first gated on FSC vs. SSC and SSC-A vs. SSC-W, CD19 vs. CD33 was gated from hCD45+. **(B)** Gating strategy in mock-treated (top) and edited (bottom) animals to quantify engraftment of HSPCs (CD34+CD38low) (Figure 2G) and HDR frequency in HSPCs (Figure 2E). hCD45 vs. mCD45 was first gated on FSC vs. SSC and SSC-A vs. SSC-W, CD34 vs. CD38 was gated on CD45+, and GFP+ was gated on CD34+CD38low.

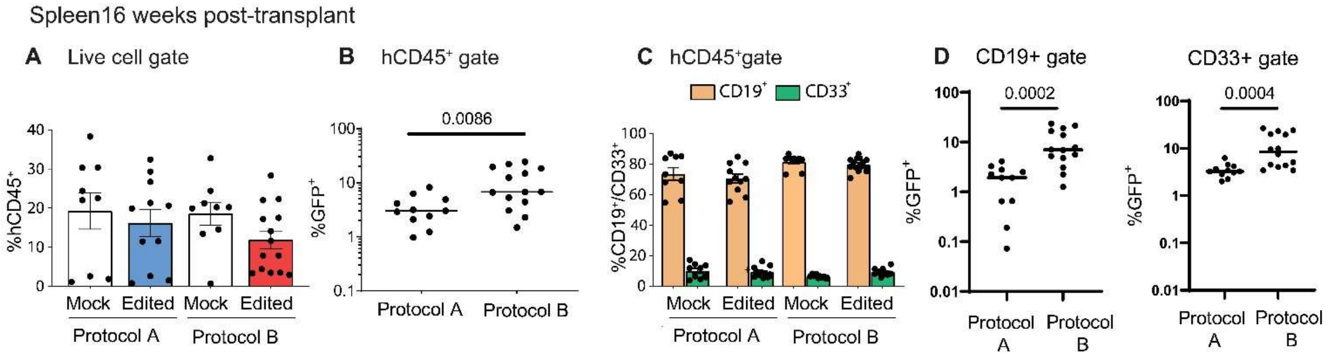

Figure S4. Long-term engraftment of GFP-T3 edited HSPCs in the spleen of NBSGW mice. **(A)** Bar graphs showing hCD45+ engraftment in spleens of NBSGW mice, 12-16 weeks post adoptive transfer of mock or edited CD34+ cells cultured using protocol A or B. Mean +/- SEM. **(B)** HDR frequency in bulk (hCD45+) cells recovered from spleens of NBSGW mice. Mann-Whitney U-test Median. **(C)** Bar graphs depicting distribution of B (CD19+) and myeloid (CD33+) cells within human CD45+ cells recovered from mice. Mean +/- SEM. **(D)** HDR frequency in B cell (left) or myeloid (right) compartments recovered from spleens of NBSGW mice. Mann-Whitney U-test. Median.

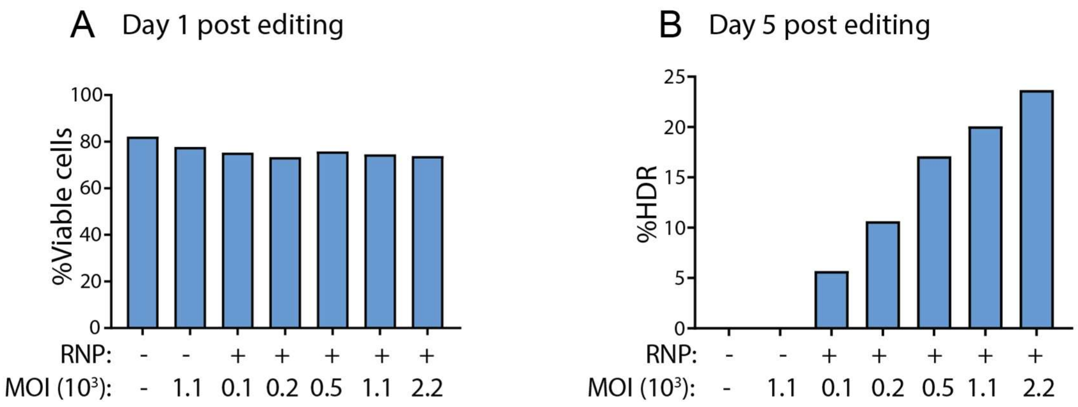

Figure S5. Optimization of AAV.FOXP3. cDNA MOI for achieving optimal cell viability and targeting efficiency. (A) Bar graphs presenting proportion of viable cells one day post-editing with T3 RNP and AAV.FOXP3.cDNA vector at the indicated MOIs. **(B)** HDR editing frequency determined by ddPCR of gDNA collected 5 days post-editing.

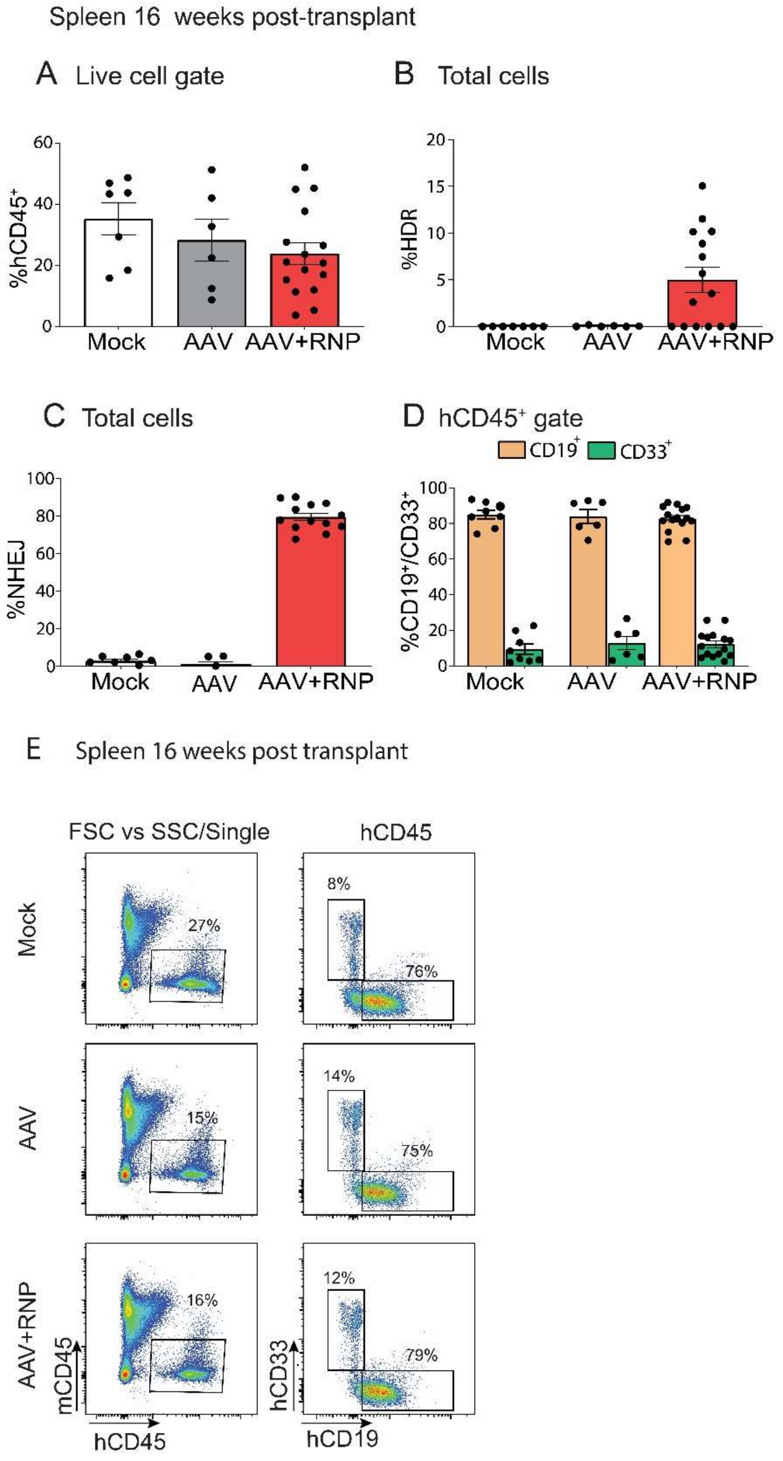
Spleen 16 weeks post-transplant
A Live cell gate
%hCD45+
0
20
40
60
Mock
AAV
AAV+RNP
B Total cells
%HDR
0
5
10
15
20
Mock
AAV
AAV+RNP
C Total cells
%NHEJ
0
20
40
60
80
100
Mock
AAV
AAV+RNP
D hCD45+ gate
CD19+
CD33+
%CD19+/CD33+
0
20
40
60
80
100
Mock
AAV
AAV+RNP
E Spleen 16 weeks post transplant
FSC vs SSC/Single
hCD45
Mock
27%
8%
76%
AAV
15%
14%
75%
AAV+RNP
16%
12%
79%
mCD45
hCD45
hCD33
hCD19

Figure S6. Long term engraftment of cDNA-edited HSPCs in the spleens of NBSGW mice. (A) Bar graphs depicting percent engraftment of human CD45+ cells in the spleens of NBSGW mice 16 weeks post transplantation. **(B-C)** Bars graphs showing percentage of bone marrow cells containing alleles with HDR- **(B)** and NHEJ-edits **(C)** as determined by ddPCR of splenic gDNA. **(D)** Bar graphs depict distribution of CD19+ B cells and CD33+ cells within the human $CD45^+$ splenic fraction. **(E)** Representative flow cytometry plots of splenocytes from NBSGW mice transplanted with $CD34^+$ cells receiving the indicated edits. All data are presented as mean +/- SEM.

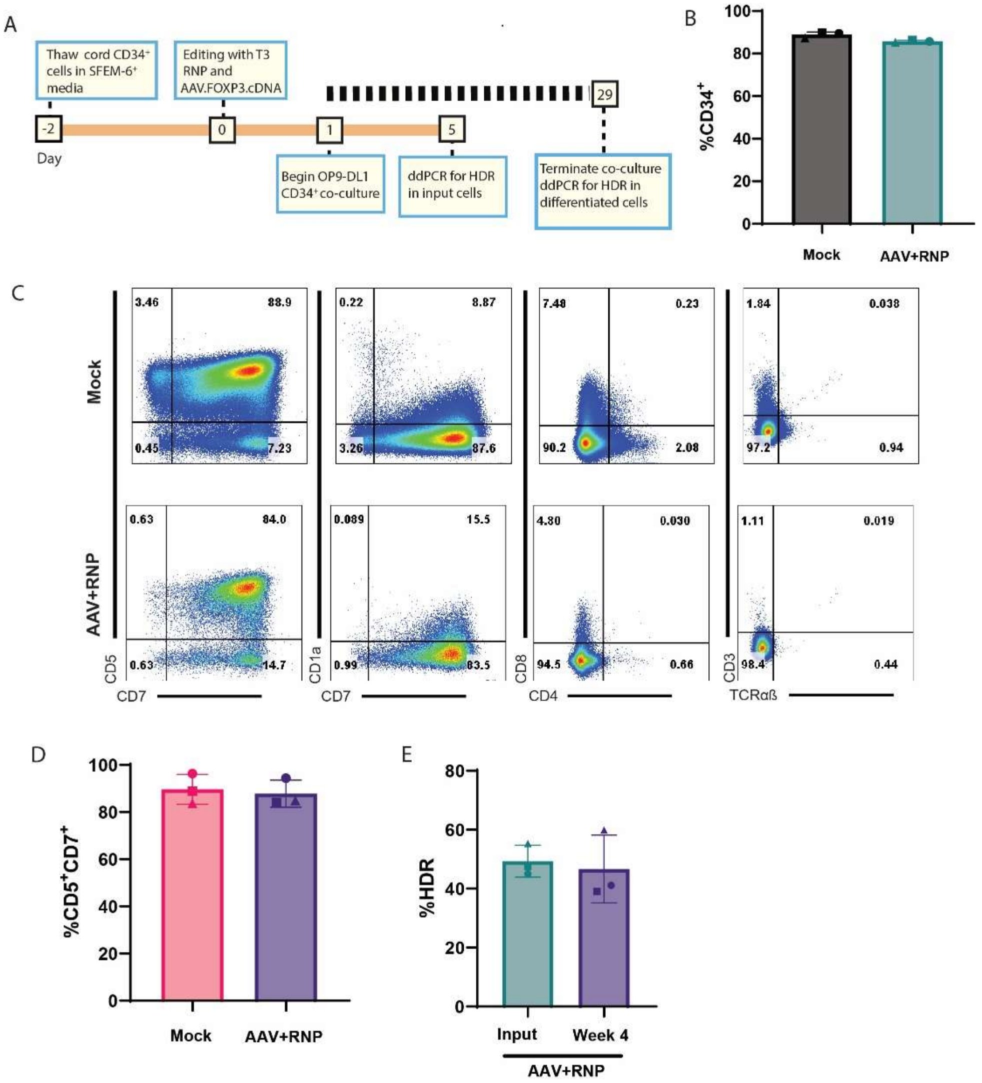

Figure S7. OP9-DL1 differentiation of AAV.FOXP3.cDNA edited CB-CD34^{+}. **(A)** Timeline of CB CD34^{+} HSPC differentiation towards T lymphocyte lineage in OP9-DL1 in vitro differentiation system. **(B)** Proportion of HSPCs (CD34^{+}) at the initiation of OP9-DL1 differentiation. **(C)** Phenotype of differentiated mock and edited CB-CD34^{+} cells after 28 days of differentiation in the ATO system. CD5 vs. CD7, CD1a vs. CD7, CD4 vs. CD8, and CD3 vs. TCRαß plots are gated by FSC vs SSC, SSC-A vs. SSC-W, hCD45+ CD34- and CD14-CD56-CD19- to exclude monocytes, NK cells, and B cells. **(D)** Proportion of pre T-1 cells (CD5+CD7+) at the termination of OP9-DL1 differentiation (gated on hCD45+CD34- and CD14-CD56-CD19-). **(E)** Proportion of HDR-edited cells at the initiation and termination of OP9-DL1 differentiation quantified by ddPCR Data represents three CD34^{+} donors in two independent studies. Bar graphs represent mean +/- SEM.

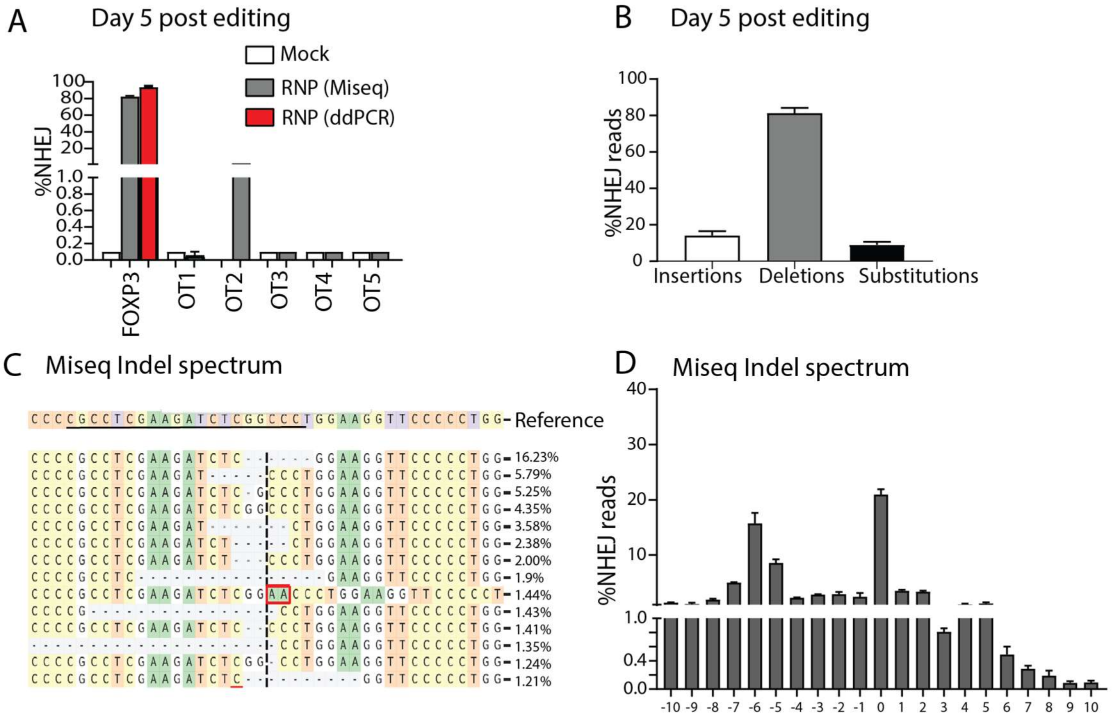

Figure S8. Analysis of on and off-target cleavage of sgRNA T3 in HSPCs using Miseq. (A) Graphs showing on and off target cleavage at *FOXP3* and the top 5 top off target sites for sgRNA T3. NHEJ rates determined by ddPCR at *FOXP3* are shown for comparison. N=3 independent donors for FOXP3 and N=2 for OT1 through 5. Genomic DNA collected 5 days post nucleofection was used for the on and off target analysis. **(B)** Graphs showing distribution of indels at *FOXP3* locus using Miseq platform. N=3 independent $CD34^+$ donors. **(C)** Summary of frequently observed indels in $CD34^+$ cells nucleofected by T3 RNP as determined by Miseq. The sequence of T3 sgRNA is underlined. **(D)** Bar graphs showing frequencies of indels in RNP nucleofected $CD34^+$ cells. N=3 independent $CD34^+$ donors.

Table S1: Summary of CD34⁺ HSPCs editing protocols

Parameter	Protocol A	Protocol B
Culture media	SCGM with TPO, SCF, IL-6, Flt-3L (100ng/ml)	SFEMII with TPO, SCF, IL-6, Flt-3L (100ng/ml), SR1(1uM), UM171(35nM)
Pre-stimulation time	48 hr	48 hr
Cell density at the time of plating	1 x 10^6 cells/ml	2.5 x 10^5 cells/ml
Method of transfection	Electroporation or nucleofection	Nucleofection
Time of AAV addition	Immediately post electroporation	Immediately post electroporation
Plating density of cells post transfection	0.8 ×10^6 cells/ml	1×10^6 cells/ml

Table S2: Off target sites predicted for sgRNA T3 using CCtop

Coordinates	Target sequence	PAM	Region	Gene name	Distance from nearest gene
chr6:168307902-168307924	CACCGTGG[AGATCTCGGCCC]	GGG	Exon	DACT2	0
chr9:133485387-133485409	CCCCTCGG[AGACCTCGGCCC]	TGG	-	SLC2A6	6250
chr14:37584848-37584870	CGCGGCGG[AGCTCTCGGCCC]	GGG	-	FOXA1	5114
chr1:26022747-26022769	TGCCTCCC[AGACCTCGGCCC]	GGG	Exon	EXTL1	0
chr4:2439391-2439413	GACCTCGA[AGATCTGGGCCC]	AGG	Intron	CFAP99	1227

Table S3 – Antibodies

**Reagent or resource**	**Source**	**Identifier**
Human CD45 eFluor450, clone HI30	ThermoFisher Scientific	48045941
Mouse CD45 APC, clone 30-F11	ThermoFisher Scientific	17-0451-82
Human CD33 PE, clone WM53	BD Biosciences	555450
Human CD19 PE Cy7, clone HIB19	ThermoFisher Scientific	25019942
Human CD34 APCCy7, clone 581	BioLegend	343514
Human CD38 PerCPCy5.5, clone HIT2	BD Biosciences	551400
Human CD90 APC, clone 5E10	BD Biosciences	561971
Human CD133 PE, clone AC133	Miltenyi Biotec	130-113-108
Human CD4 eFluor450, clone OKT4	ThermoFisher Scientific	48004842
Human CD8 PerCPCy5.5, clone RPA-T8	BD Biosciences	560662
Human CD25 PECy7, clone 2A3	BD-Biosciences	335789
Human CD127 BV510, HIL-7R-M21	BD-Biosciences	563086
Human FoxP3 PE, clone 254D	Biolegend	320208
Human CD4 Alexa700, clone OKT4	ThermoFisher Scientific	50-168-56
Human CD3 BV786, clone SK7	BioLegend	344842
Human CD4 BV605, clone RPA-T4	BioLegend	300556

Human CD8 Alexa700, clone SK1	BioLegend	344724
Human CD19 PECy7, clone HIB19	BioLegend	302215
Human CD56 PECy7, clone HCD56	BioLegend	318318
Human CD14 PECy7, clone 61D3	ThermoFisher Scientific	56-0149-42
Human TCRab, PE, clone IP26	BioLegend	306708
Human CD5 APC, clone L17F12	BioLegend	364016
Human CD7 FITC, clone CD7-6B7	BioLegend	343104
Human CD1a PerCPCy5.5, clone HI149	BioLegend	300130
Human CD8 Alexa700, clone RPA-T8	BD Biosciences	557945

Table S4 - Experimental model/strains

**Resource or reagent**	**Source**	**Identifier**
NBSGW<br><br>NOD.Cg-KitW-41J Tyr +<br><br>Prkdcscid Il2rgtm1Wjl/ThomJ	Jackson Laboratory	Stock 026622

Table S5– Biological samples - cell lines

**Resource or reagent**	**Source**	**Identifier**

OP9-DL1 cells	Dr. Irwin Bernstein (University of Washington)	N/A
MS5-DLL4 cells	Dr. Gay Crooks (University of California Los Angeles)	N/A

Table S6 – Biological samples – primary cells

**Resource or reagent**	**Source**	**Identifier**
Mobilized peripheral blood $CD34^+$ HSPCs	Fred Hutch Co-operative Center for Excellence in Hematology	N/A
Cord blood $CD34^+$ HSPCs	STEMCELL Technologies	70008.5
Cord blood $CD34^+$ HSPCs from IPEX patient	Seattle Children's Hospital	N/A
Peripheral blood mononuclear cells	Fred Hutch Co-operative Center for Excellence in Hematology	N/A
Peripheral blood mononuclear cells from IPEX patient	Seattle Children's Hospital	N/A

Table S7– Oligonucleotides and probes

**Reagent or resource**	**Source**	**Identifier**

NHEJ forward CACGTGTGACTCCTTTCCC	IDT	N/A
NHEJ reverse CACGTGTGACTCCTTTCCC	IDT	N/A
T3_NHEJ probe AGCTGGGCGAGGCTCCT	IDT	N/A
T9_NHEJ probe AGCTGGGCGAGGCTCCT	IDT	N/A
NHEJ Control forward CGACACTTCACCCCTTTTCT	IDT	N/A
NHEJ Control reverse CTCCCCAATGTGCCTATGAG	IDT	N/A
NHEJ Control Probe GTGGCGGTGACTGGGATGGC	IDT	N/A
HDR forward GAGCAAAGACCCCAACGAGA	IDT	N/A
HDR reverse GACTTGGGGGTTCTGTGAAG	IDT	N/A
HDR GFP probe GCATGGACGAGCTGTACAAG	IDT	N/A
HDR cDNA forward GAACCCTGAACGAGATCTACC	IDT	N/A
HDR cDNA reverse CTGTGGTTCAGCCTGACTCG	IDT	N/A
HDR cDNA probe TCCTGGTTAGTTCTTGCCAC	IDT	N/A
HDR control *ActB* forward ACTCTGCAGGTTCTATTTGC	IDT	N/A
HDR control *ActB* reverse AATGATCTGAGGAGGGAAGG	IDT	N/A
HDR probe *ActB* ATCAAGGTGGGTGTCTTTCC	IDT	N/A
HDR control *CCR5* forward AAAGATTTGCAGAGAGATGAGT	IDT	N/A
HDR control *CCR5* reverse GCCAAGCAATGAAGTTTTGT	IDT	N/A
HDR probe *CCR5* CCTGGGCAACATAGTGTGATC	IDT	N/A

FOXP3co transcript forward<br><br>CAGCTGGTCCTGGAAAAAGA	IDT	N/A
FOXP3co transcript reverse<br><br>AACATCCGGGTAAACCAGTG	IDT	N/A
FOXP3co transcript probe TCCTGCACAACATGGACTAC	IDT	N/A
TaqMan Gene Expression Assay (FOXP3 endogenous)	ThermoFisher Scientific	Hs01085831_g1
TaqMan Gene Expression Assay (HPRT)	ThermoFisher Scientific	Hs99999909_m1

Table S8 – Reagent list

**Reagent**	**Source**	**Identifier**
Recombinant human TPO	Peprotech	300-18
Recombinant human SCF	Peprotech	300-07
Recombinant Human Flt3-Ligand	Peprotech	300-19
Recombinant human IL-6	Peprotech	200-06D
Recombinant human IL-7	Peprotech	200-07
StemRegenin1	STEMCELL Technologies	72344
UM171	ApexBio	NC0806207
HEPES	Gibco	15630080
Glutamax	Gibco	35050061
B-mercaptoethanol	Sigma-Aldrich	M6250
DNeasy Blood and tissue Kit	Qiagen	69506
CellGenix® GMP SCGM	CellGenix	20802-0500

Stem Cell Growth Medium		
Stemspan SFEMII	STEMCELL Technologies	09655
RPMI-1640	Gibco	11875093
Alpha MEM with Nucleosides	STEMCELL Technologies	36450
DMEM	Gibco	11965092
Fetal bovine serum	Omega Scientific	FB-11
Busulfan (Busulfan injections)	Otsuka America Pharmaceutical	59148-070-90
CRISPRevolution sgRNA EZ Kit	Synthego	N/A
SpyFi Cas9 Nuclease	Aldevron	N/A
Agencourt AMPure XP	Beckman Coulter	A63881
MiSeq 500 CycleV2 kit	Illumina	MS-102-2003
PrimeSTAR GXL DNA polymerase	Clontech	R050B
ddPCR Supermix for Probes without UTP	Bio-Rad	1863025
Droplet Generation Oil for Probes	Bio-Rad	1863005
Droplet reader oil	Bio-Rad	1863004
RNeasy mini kit	Qiagen	74106
Maxima First Strand cDNA Synthesis Kit for qRT-PCR	Thermofisher Scientific	FERK1672
CD34 MicroBead Kit, human	Miltenyi Biotech	130-046-702
EasySep™ Human $CD4^+$ T cell enrichment kit	STEMCELL Technologies	19052

EasySep™ Human CD4+CD127lowCD25⁺ Regulatory T Cell Isolation Kit	STEMCELL Technologies	18063
True-Nuclear Transcription Factor buffer set	BioLegend	424401
Dynabeads Human T-Expander CD3/CD28	Gibco	11141D

Table S9 – Software and algorithm

Reagent or resource	Source	Identifier
QuantaSoft Analysis Software	Bio-Rad	1864003
Prism 7.03 or later software package	Graph Pad Software	N/A
CRISPResso	[51]	N/A

Table S10 – Other materials

Reagent or resource	Source	Identifier
QX200 Droplet reader	Bio-Rad	1864001
QX200 Droplet Generator	Bio-Rad	N/A
LSRII	BD Biosciences	N/A
BD FACSAria II cell sorter	BD Biosciences	N/A
